# Low latency global carbon budget reveals land sink decline during 2023 to 2024 El Niño

**DOI:** 10.1016/j.isci.2026.117053

**Published:** 2026-08-10

**Authors:** Piyu Ke, Philippe Ciais, Yitong Yao, Stephen Sitch, Wei Li, Yidi Xu, Xiaomeng Du, Xiaofan Gui, Ana Bastos, Sönke Zaehle, Ben Poulter, Thomas Colligan, Auke M. van der Woude, Wouter Peters, Zhu Liu, Zhe Jin, Xiangjun Tian, Yilong Wang, Junjie Liu, Sudhanshu Pandey, Chris O’Dell, Jiang Bian, Chuanlong Zhou, John B. Miller, Xin Lan, Jefferson Goncalves De Souza, Michael O’Sullivan, Pierre Friedlingstein, Guido R. van der Werf, Glen P. Peters, Shilong Piao, Frédéric Chevallier

**Affiliations:** 1 Laboratoire des Sciences du Climat et de l'Environnement, LSCE/IPSL, CEA-CNRS-UVSQ, Université Paris-Saclay, Gif-sur-Yvette 91191, France; 2 Department of Earth System Science, Tsinghua University, Beijing 100084, China; 3 Faculty of Environment, Science and Economy, University of Exeter, Exeter EX4 4QF, UK; 4 Department of Earth and Environmental Engineering, Columbia University, New York, NY 10027, USA; 5 Machine Learning Group, Microsoft Research, Beijing 100080, China; 6 Institute for Earth System Science and Remote Sensing, Leipzig University, Leipzig 04103, Germany; 7 Biogeochemical Signals Department, Max Planck Institute for Biogeochemistry, Jena 07745, Germany; 8 Earth Sciences Division, NASA Goddard Space Flight Center, Greenbelt, MD 20771, USA; 9 Earth System Science Interdisciplinary Center, University of Maryland, College Park, MD 20740, USA; 10 Environmental Sciences Group, Department of Meteorology and Air Quality, Wageningen University, Wageningen 6708 PB, the Netherlands; 11 Institute of Carbon Neutrality, Sino-French Institute for Earth System Science, College of Urban and Environmental Sciences, Peking University, Beijing 100871, China; 12 State Key Laboratory of Tibetan Plateau Earth System, Environment and Resources (TPESER), Institute of Tibetan Plateau Research, Chinese Academy of Sciences, Beijing 100101, China; 13 Jet Propulsion Laboratory, California Institute of Technology, Pasadena, CA 91011, USA; 14 Cooperative Institute for Research in the Atmosphere, Colorado State University, Fort Collins, CO 80523, USA; 15 National Oceanic and Atmospheric Administration Global Monitoring Laboratory, Boulder, CO 80303, USA; 16 Cooperative Institute for Research in Environmental Sciences, University of Colorado Boulder, Boulder, CO 80303, USA; 17 Laboratoire de Météorologie Dynamique, IPSL, CNRS, ENS, Université PSL, Sorbonne Université, École Polytechnique, Paris 75005, France; 18 CICERO Center for International Climate Research, Oslo 0349, Norway; 19 Spark Climate Solutions, Covina, CA 91723, USA

**Keywords:** global CO2 budget, tropical flux changes during El Niño 2023/24, machine-learning emulators of carbon models

## Abstract

El Niño droughts can weaken tropical land carbon uptake and accelerate atmospheric CO_2_ growth. We updated the low-latency global CO_2_ budget through June 2024 using near-real-time fossil emissions, four dynamic global vegetation models (DGVMs), ocean carbon emulators, and three OCO-2 atmospheric inversions. From July 2023 to June 2024, atmospheric CO_2_ grew by 3.66 ± 0.09 ppm yr^−1^, the highest July-to-July rate since 1979; after detrending, the anomaly was 1.1 ppm yr^−1^, comparable to previous major El Niño events. The anomaly was mainly driven by a 2.24 GtC yr^−1^ weakening of the net land sink, partly offset by 0.38 GtC yr^−1^ stronger ocean uptake. The tropics accounted for 97.5% of the land anomaly, led by the Amazon, central Africa, and Southeast Asia. This update shows how recent El Niño droughts drove the high CO_2_ growth rate through mid-2024 and how low-latency budgets can track carbon cycle extremes.

## Highlights

• CO_2_ growth reached a record high during the 2023 to 2024 El Niño
• Tropical drought drove most of the weakened land sink
• OCO-2 inversions and DGVMs agree on tropical land sink weakening
• Low-latency budgets track carbon cycle responses to climate extremes

## Teaser

Environmental science; Global change

## Introduction

**Figure 1:**
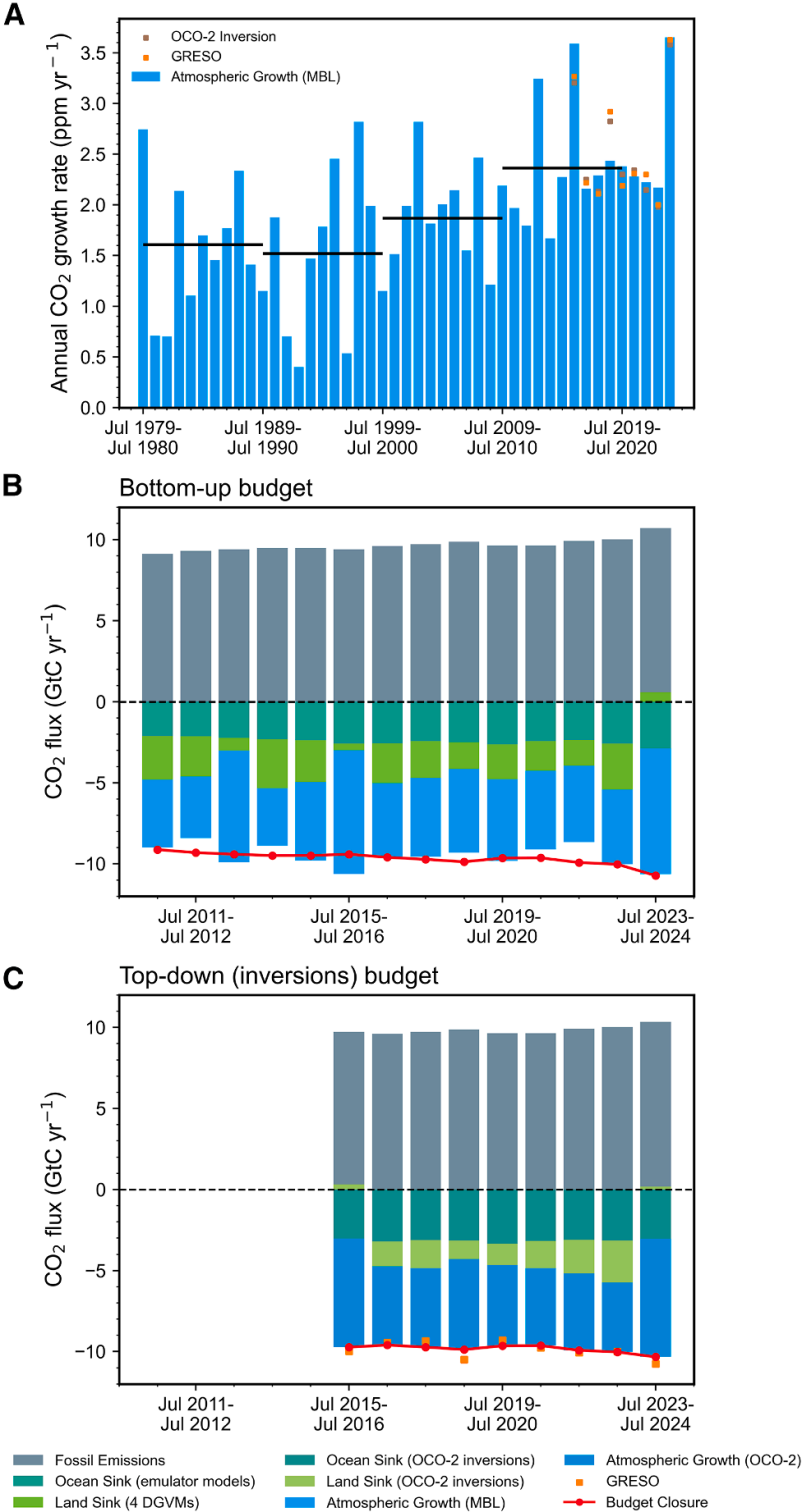
Atmospheric CO_2_ growth rate (1979–2024) and carbon budget (2010–2024), calculated for July-July annual periods (A) Annual June-July to June-July growth rate from marine boundary layer surface stations (MBL, blue bars), OCO-2 (dark brown squares), and the growth rates using satellite observations approach from Pandey et al.^12^ (GRESO, orange squares). Our analysis is based on MBL. MBL is the average of many surface locations, which gives a better estimate of the global increase in atmospheric CO_2_ compared to the single high-altitude MLO estimate. (B) Global CO_2_ bottom-up budget obtained with our estimates of fossil CO_2_ emissions, DGVM-based net land CO_2_ flux, net ocean sink from ocean model emulators, and annual CO_2_ growth rates from MBL. The red curve is −1 ∗ fossil emissions, and the difference between the bars and this curve is the imbalance of the bottom-up budget. (C) Global CO_2_ top-down budget obtained with our estimates of fossil CO_2_ emissions and inversions assimilating OCO-2 atmospheric CO_2_ mixing ratios. By design, inversions match OCO-2 data, and their growth rate is the blue bar. The orange squares are the CO_2_ growth rate obtained directly from OCO-2 data using the GRESO model.

El Niño events strongly influence global carbon cycle dynamics by inducing severe drought conditions, particularly in tropical terrestrial ecosystems.^1^^,^^2^ Such droughts decrease plant productivity and terrestrial carbon uptake, consequently elevating atmospheric CO_2_ concentrations.^3^^,^^4^ Historical El Niño events, notably the strong events of 1997/98 and 2015/16, led to significant anomalies in global atmospheric CO_2_ growth rates.^5^^,^^6^ The recent 2023/24 El Niño event, which began in June 2023 and ended in March 2024, was of moderate intensity (Multivariate El Niño-Southern Oscillation (ENSO) Index (MEI) = 0.71).^7^ However, despite its moderate strength compared to previous major events, the 2023/24 El Niño notably increased atmospheric CO_2_ concentrations due to extensive drought-induced declines in the tropical land carbon sink, underscoring the sensitivity of terrestrial ecosystems to even moderate climate extremes.^8^^,^^9^ The global CO_2_ atmospheric growth rate in the decade 2013–2022 averaged 2.43 ± 0.01 ppm yr^−1^. We previously reported a 2.82 ± 0.08 ppm yr^−1^ growth rate in 2023 based on the global average of marine boundary layer sites (MBLs) from the National Oceanic and Atmospheric Administration (NOAA) network, which has been updated by NOAA to 2.74 ± 0.08 ppm yr^-1.^^5^^,^^6^^,^^9^ The development of an El Niño in the second half of the year 2023 explained part of this high growth rate anomaly, notably with an extreme drought in the Amazon.^9^ However, that 2023-only assessment left open whether the land-sink decline was a short-lived response to the late-2023 Amazon drought or a persistent anomaly spanning the decay phase of the 2023/24 El Niño. Recent related studies have also examined the 2024 land-carbon anomaly from complementary perspectives.^10^^,^^11^ Here, we extend the low-latency budget to the first six months of 2024, when the El Niño continued until March 7, and evaluate the full July 2023-to-June 2024 carbon budget using both bottom-up estimates and Orbiting Carbon Observatory-2 (OCO-2)-constrained atmospheric inversions. The monthly annual CO_2_ growth rate calculated as the difference between the monthly smoothed CO_2_ mole fraction at the MBL stations and that of twelve months before, following the NOAA method, reached a peak of 3.67 ± 0.13 ppm yr^−1^ in June 2024 (Figure S1A). The annual CO_2_ growth rate from June-July 2023 to June-July 2024 averaged 3.66 ± 0.09 ppm yr^−1^ at the MBL stations (Figure 1A). For comparison, the June-July annual averages from 2013 to 2022 were 2.46 ± 0.01 ppm yr^-1.^^5^^,^^6^ The growth rate derived from independent OCO-2 satellite observations for June–July 2023 to June–July 2024 was 3.63 ± 0.10 ppm yr^−1^ using the growth rates using satellite observations data-driven approach (GRESOs) from Pandey et al.^12^ The growth rate derived from our flux inversions of OCO-2 observations from July 2023 to July 2024 was 3.65 ± 0.12 ppm yr^−1^, thus almost equal to GRESO (Section S1). Overall, the MBL and the OCO-2-derived growth rates show excellent agreement on annual scales, as shown in Figures 1A and S1.

In this study, we provide an updated global and regional CO_2_ budget covering the period from July 1^st^, 2023 to July 1^st^, 2024. To rapidly capture recent changes in the carbon cycle associated with the 2023/24 El Niño, we combined low-latency (available within one to six months after observations) surface CO_2_ flux datasets derived from both top-down (atmospheric inversions) and bottom-up (model-based) approaches. Fluxes for this period were compared to those from previous years calculated using the same methodology (Figure 1B).To update global fossil fuel and cement CO_2_ emissions for the first half of 2024 (H1-2024) from July 1^st^ 2023 and July 1^st^ 2024, we used two complementary data sources. The first was daily data from the Carbon Monitor project.^13^^,^^14^^,^^15^ The second was emissions estimates from the Global Carbon Project.^16^ Detailed monthly emission allocations and uncertainty estimates are described in the STAR Methods.

For the bottom-up global land carbon flux, we assessed the net land carbon flux which corresponds to the sum of anthropogenic land-use change emissions (E_LUC_) and carbon uptake on other lands not affected by land-use change (S_LAND_) in the Global Carbon Budget (see STAR Methods). We used four dynamic global vegetation models (DGVMs),^17^^,^^18^^,^^19^^,^^20^^,^^21^^,^^22^^,^^23^^,^^24^ joint UK land environment simulator (JULES), OCN and ORCHIDEE-MICT previously used by Ke et al.^9^ and Lund-Potsdam-Jena Earth Observation SIMulator (LPJ-EOSIM).^21^ Net land CO_2_ fluxes from the four DGVMs were driven by ERA5 atmospheric reanalysis forcing data until July 1^st^ 2024 at 0.5 × 0.5° resolution with the same protocol as in Ke et al.^9^ (see STAR Methods). Although we use four DGVMs, their flux anomalies for previous years closely matched the multi-mean of all the models used in previous carbon budget assessments (Figure S8).^9^^,^^16^^,^^25^ This gives us confidence that our small sample of low-latency DGVMs can describe the net land carbon flux anomaly for the period from July 2023 to July 2024.

Three of our four DGVMs (OCN, ORCHIDEE-MICT and LPJ-EOSIM) simulate fire emissions as part of the net land CO_2_ flux, in the range of 1.96–6.33 GtC yr^−1^ from July 2023 to July 2024. This min-max range of model-based fire emissions is much larger than that from the observation-based products global fire emissions database version 4.1s (GFED4.1s)^26^ and global fire assimilation system (GFAS) (https://atmosphere.copernicus.eu/global-fire-monitoring), which are based on satellite observations of burned area and combustion energy, respectively. These three DGVMs simulate fires prognostically using population density, lightning ignitions, climate, and simulated fuel moisture, but have weaknesses in capturing extreme forest fires and tropical forest degradation fires. We therefore corrected the net land CO_2_ flux of OCN, ORCHIDEE-MICT and LPJ-EOSIM by subtracting their original fire emissions and adding the mean of GFED4.1s and GFAS (see STAR Methods). JULES does not simulate fire in the configuration used here, and we therefore directly added the same observation-based fire emissions to its net land CO_2_ flux. The GFED4.1s and GFAS fire emissions from July 2023 to July 2024 are 2.15 GtC yr^−1^ and 1.88 GtC yr^−1^, respectively, compared to 4.31 ± 2.20 GtC yr^−1^ in the three fire-enabled DGVMs during the same period.

In addition to the four low-latency DGVMs listed above, we estimated the land CO_2_ flux using the SiB4 land flux model constrained for the global net CO_2_ flux by carbon tracker Europe high-resolution (CTE-HR) system^27^ (0.1° × 0.1° globally) with a spatially upscaled version of the SiB4 land CO_2_ flux model forced with ERA5 atmospheric reanalysis.^28^ The long-term mean total ecosystem respiration is scaled so that the net ecosystem exchange (NEE) is equal to the long-term mean NEE of the carbon tracker Europe inversion, while gross primary production (GPP) and respiration vary from hour-to-hour and across 10 plant-functional types. We also estimated the natural land carbon flux (S_LAND_) using machine learning (ML) emulators of 19 DGVMs contributing to the global carbon budget 2024.^16^ Each emulator was trained using as input features monthly gridded climate data, CO_2_ fluxes, and annual vegetation maps of each native model until Dec. 2023 (Section S3). These emulators successfully reproduce the monthly CO_2_ fluxes and the flux anomalies of the native DGVMs at 0.5 × 0.5° globally for test years that were not included in the training set (Figure S3). In the analyses later in discussion, the four ERA5-driven DGVMs provide the primary bottom-up estimate for the July-to-July global land budget and for the H1-2024 land-flux anomalies, while the mean of the three OCO-2 inversions provides the primary top-down constraint. The SiB4 product and the 19 DGVM emulators are used as independent cross-checks rather than to define the central budget values. This distinction is important because the emulators reproduce the behavior of the GCB2024 DGVM family, which was run with Climatic Research Unit-Japanese Reanalysis (CRU-JRA) forcing, and therefore provide a partially independent benchmark against the ERA5-driven low-latency DGVMs, especially for tropical anomalies and regional attribution (Figures S4 and S7).

For the bottom-up ocean carbon sink, we used ML emulators of each ocean biogeochemical and data-driven model as in Ke et al.^29^ used for previous years in the global carbon budget assessments.^30^ These ocean CO_2_ flux emulators are trained by temporal trends and patterns of 16 original ocean models used by global carbon budget 2022^30^ with input data being atmospheric CO_2_ mixing ratio, sea surface temperature, ice cover, sea surface height, sea level pressure, sea surface salinity, mixed layer depth, wind speed and chlorophyll (see STAR Methods and Section S2). After being trained until Dec. 2021, the emulators were run forward to make a projection of the monthly ocean sink at 1° spatial resolution for H1-2024, since only one ocean data-driven model provides low-latency fluxes covering this period.^31^

For the top-down budget, we used inversion models assimilating column-averaged CO_2_ dry air mole fraction retrievals from the OCO-2 satellite (Level 4 ACOS V11^32^), which cover H1-2024 while only a limited set of *in situ* surface measurements is yet available for that period. Satellite observations from OCO-2 provide similar skill in estimating surface CO_2_ flux as inversions that assimilate the more accurate but sparse surface station measurements.^33^ Moreover, the OCO-2 data have better coverage than the surface network across the tropics, which is an important advantage for separating CO_2_ fluxes between the northern hemisphere and the tropics, and investigating flux anomalies between tropical continents, in particular between Amazon and South-east Asia affected by drought from July 2023 to July 2024. We used here three inversions: Copernicus atmosphere monitoring service (CAMS), Carbon Monitoring System Flux (CMS-Flux), and Global ObservatioN-based system for monitoring Greenhouse GAses (GONGGA),^33^^,^^34^^,^^35^^,^^36^ assimilating the same OCO-2 column CO_2_ mixing ratio data product.^37^^,^^38^ The native spatial resolution of the CAMS monthly CO_2_ fluxes is 90 km, which better matches that of our DGVMs (0.5°) and allows us to gain more insights into regional details of CO_2_ fluxes without the usual smoothing effect of inversions. The spatial resolution of GONGGA is 2° by 2.5° (latitude × longitude), and that of CMS-Flux is 4° by 5°. The inversion CO_2_ fluxes were corrected for background natural fluxes related to the river loop of the carbon cycle as in Regnier et al.^39^ to provide anthropogenic carbon fluxes comparable to those simulated by bottom-up models.

The higher CO_2_ growth rate from July 2023 to July 2024 echoes the impact of extreme warming, with each month warmer than the corresponding month in any previous year. The global temperature in the year 2024 was 0.72°C above the 1991–2020 average and 1.6°C warmer than the 1850–1900 pre-industrial level,^40^ with extreme heat waves in the northern mid-latitudes and tropics, and drought in Central and South America, Central and southern Africa.^41^^,^^42^ The period of H1-2024 marked the continuation of the El Niño that started in June 2023 and continued until March 2024 according to the MEI from NOAA Physical Sciences Laboratory, with MEI >0.5 indicating El Niño conditions.^7^ Overall, the mean MEI of the 2023/24 El Niño was 0.71, that is a moderate El Niño compared to 2015/16 (MEI = 1.73) and to 1997/98 (MEI = 2.16). In the second half of 2023 (H2-2023) the Amazon experienced an extreme drought while Africa was on average wetter than usual.^9^

## Results and discussion

### The global carbon budget from July 1^st^ 2023 to June 30^th^ 2024

We assessed the global yearly CO_2_ budget for the period July 1^st^ 2023 to June 30^th^ 2024 (hereafter July-to-July) as it is not meaningful to provide a budget only during the first six months of the year 2024 when the seasonal uptake by the land is only half finished. The budget is usually based on calendar years, but here we assess the July-to-July period to reduce the latency and span the typical El Nino anomalies that are the main driver of year-to-year changes in global CO_2_ fluxes. Compared with our 2023 low-latency assessment,^9^ the key new test here is whether the land-sink weakening persisted through H1-2024 and how the resulting July-to-June budget compares with previous El Niño events. The results later in discussion show that the anomaly did persist beyond the late-2023 Amazon drought, with additional regional source anomalies emerging in tropical Africa and Southeast Asia during the decay phase of the event.

We first present the atmospheric CO_2_ growth rate for the period July 2023 to July 2024 to characterize the global carbon cycle response associated with the ongoing 2023/24 El Niño event. Figure 1A shows the June-July-to-June-July CO_2_ growth rates (hereafter July-July) from MBL atmospheric stations. The CO_2_ growth rate of 3.66 ± 0.09 ppm yr^−1^ (mean ±1 standard deviation) is a record-high value since 1979. Yet, the CO_2_ growth rate anomaly obtained after removing the long-term trend is of 1.1 ppm yr^−1^, marginally lower than the July-July anomalies of the two previous El Niño events in 1997/98 and 2015/16. Comparing July-July growth rates between MBL stations and the longest single record of Mauna Loa (MLO) shows differences ranging between 0.02 and 0.93 ppm yr^−1^ among years since 1979, with a positive difference between MBL and MLO of 0.37 ppm yr^−1^ between July 2023 and July 2024 (Figure S1B). Nevertheless, the cumulative increase of atmospheric CO_2_ from July 1979 to July 2024 is similar within 1.85 ppm between MLO and MBL, indicating that the interannual differences between the two records balance over extended time periods.

Figure 1B shows the bottom-up carbon budget, obtained from the mean of our two estimates of fossil emissions^13^^,^^14^^,^^15^^,^^16^ combined with bottom-up net land CO_2_ flux and ocean carbon fluxes. Figure 1C shows the top-down carbon budget based on the mean of the three OCO-2 inversions. Global fossil fuel and cement CO_2_ emissions were 10.14 GtC yr^−1^ (with a range of 10.14–10.15 GtC yr^−1^ from our two estimates). In the bottom-up budget, the DGVMs estimated a net land source of 0.59 ± 0.60 GtC yr^−1^ and in the top-down budget, the inversions gave a net land source of 0.09 ± 0.26 GtC yr^−1^. The mean net land CO_2_ flux of the DGVMs and inversions was a source of 0.34 ± 0.33 GtC yr^−1^. The ocean CO_2_ uptake from July 2023 to July 2024 was of 2.56 ± 0.33 GtC yr^−1^ (ocean emulators: 2.87 ± 0.56 GtC yr^−1^, inversions: 2.25 ± 0.33 GtC yr^−1^) similar to the mean of the year 2023. The bottom-up and top-down budgets both indicate a weakened land sink and an ocean sink close to its 2023 mean, but they differ in the magnitude of the land source, which is larger in the DGVMs than in the inversions. The main July-to-July budget components are summarized in Table 1.

**Table 1:** Global carbon budget for July 2023–June 2024

Diagnostic	Bottom-up	Top-down
Fossil fuel and cement CO_2_ emissions	10.14 (10.14–10.15)	9.91
Atmospheric CO_2_ growth	3.66 ± 0.09	3.65 ± 0.12
Net land CO_2_ flux	−0.59 ± 0.60	−0.09 ± 0.26
Net ocean CO_2_ flux	2.87 ± 0.56	2.25 ± 0.33
Budget residual	0.09	<0.05

Positive land and ocean fluxes denote uptake from the atmosphere; negative values denote net release to the atmosphere. Positive budget residual denotes excess net input to the atmosphere. Atmospheric CO_2_ growth is given in ppm yr^−1^; all other quantities are in GtC yr^−1^.

In Figures 1B and 1C, the red curve is a graphical budget-closure reference obtained by plotting fossil fuel and cement CO_2_ emissions with opposite signs. The budget residual (or imbalance) is the signed vertical distance between this red curve and the stacked negative terms, that is, land and ocean CO_2_ fluxes together with the atmospheric CO_2_ growth term; equivalently, it can be written as fossil fuel and cement emissions plus land and ocean CO_2_ fluxes minus atmospheric CO_2_ growth, with sinks negative and atmospheric growth positive. For July 2023 to July 2024, the bottom-up budget residual is 0.09 GtC yr^−1^ using the growth rate of MBL stations and a conversion factor of 2.124 GtC per ppm. This small positive residual indicates that the bottom-up budget slightly underestimates the net global CO_2_ sink, or equivalently slightly overestimates the net input of carbon to the atmosphere, during that period. In the top-down budget, the same graphical definition applies. The corresponding residual has an absolute magnitude smaller than 0.05 GtC yr^−1^, indicating a tighter closure of the inversion-based budget. This is expected because the inversion fluxes are constrained by atmospheric CO_2_ observations, although exact zero is not required because Figure 1C combines the ensemble mean of three inversions with an externally prescribed fossil-fuel term rather than a single internally closed inversion budget (Figure S1C).

### Carbon fluxes from January 1^st^ to June 30^th^ 2024

From January 1^st^ to June 30^th^ 2024 (H1-2024), which covers the second year of the 2023/24 El Niño, global fossil emissions reached 5.12 GtC, an increase of 0.04 GtC (0.8%) compared to the same period of 2023. Although this increase is relatively modest, atmospheric CO_2_ concentrations continued to rise at a fast rate, indicating a significant reduction in carbon sinks during this period. The main H1-2024 global fluxes and anomalies are summarized in Table 2.

**Table 2:** Global fossil fuel and global/regional land carbon fluxes or anomalies for H1-2024

Diagnostic	DGVMs	Inversions
Fossil fuel and cement CO_2_ emissions (H1-2024)	5.12	5.12
Global net land CO_2_ flux (H1-2024)	1.37 ± 2.06	0.71 ± 0.42
Global net land CO_2_ flux anomaly (H1-2024)	−0.99 ± 0.56	−0.29 ± 0.05
Northern land CO_2_ flux (>30°N; H1-2024)	2.01 ± 1.92	1.18 ± 0.40
Tropical land CO_2_ flux (H1-2024)	−0.39 ± 0.31	−0.49 ± 0.04
Northern land CO_2_ flux anomaly (>30°N; JFM-2024)	−0.15 ± 0.25	0.07 ± 0.06
Northern land CO_2_ flux anomaly (>30°N; AMJ-2024)	0.34 ± 0.07	0.01 ± 0.09
Tropical South America anomaly (north of 30°S; JFM-2024)	−0.28 ± 0.15	−0.03 ± 0.04
Tropical Africa anomaly (JFM-2024)	−0.03 ± 0.14	−0.09 ± 0.01

Positive fluxes denote uptake from the atmosphere; negative values denote net release to the atmosphere. Positive anomalies denote a stronger sink or a weaker source relative to the 2015–2022 mean of the same period; negative anomalies denote a weaker sink or a stronger source. All quantities are in GtC. H1 values are integrated over January–June 2024. JFM and AMJ anomalies are three-month anomalies.

The net land CO_2_ flux during H1-2024 was a net uptake of 1.37 ± 2.06 GtC in the four DGVMs (note the large uncertainty) compared to 0.71 ± 0.42 GtC in the three inversions. We have a significant uptake as H1-2024 covers roughly half of the growing season in the northern hemisphere, causing a strong periodic land uptake of CO_2_, evidenced by the drawdown of the CO_2_ atmospheric mixing ratio from April to mid-July at northern stations.^43^ The Northern land regions, north of 30°N, were a net carbon sink of 2.01 ± 1.92 GtC in the DGVMs and 1.18 ± 0.40 GtC in the inversions. On the other hand, the Tropical lands were a net source of 0.39 ± 0.31 GtC in the DGVMs and 0.49 ± 0.04 GtC in the inversions during H1-2024. Fire emissions were 0.68 GtC and 0.61 GtC during H1-2024, compared to 0.86 GtC and 0.70 GtC during H1-2023, respectively, from GFED and GFAS.

The net land CO_2_ flux anomaly during H1-2024, defined as the difference from previous H1 periods between 2015 and 2022 used as a reference, was a source of 0.99 ± 0.56 GtC in the four DGVMs. In comparison, the net land CO_2_ flux anomaly was a small uptake of 0.29 ± 0.34 GtC during H1-2023 by the end of a wetter La Niña period, and a source of 1.46 ± 0.17 GtC during H2-2023 at the start of the El Niño period.^9^ The land CO_2_ flux anomaly was 0.29 ± 0.05 GtC in the OCO-2 inversions, thus smaller than the DGVM-based anomaly. Both approaches indicate a weaker-than-normal land sink in H1-2024, but the bottom-up estimate yields a substantially larger anomaly.

The ocean sink was 1.29 ± 0.13 GtC during H1-2024, in close agreement with the bottom-up models (1.51 ± 0.13 GtC). The ocean sink anomaly during H1-2024 is a sink of 0.20 ± 0.07 GtC in our bottom-up approach. Compared to the mean of the year 2023, the ocean sink during H1-2024 was 0.11 ± 0.03 GtC higher, mainly due to the continuing El Niño, which decreased CO_2_ sources in the Equatorial Pacific.^31^

### Regional flux anomalies during the first half of 2024

**Figure 2:**
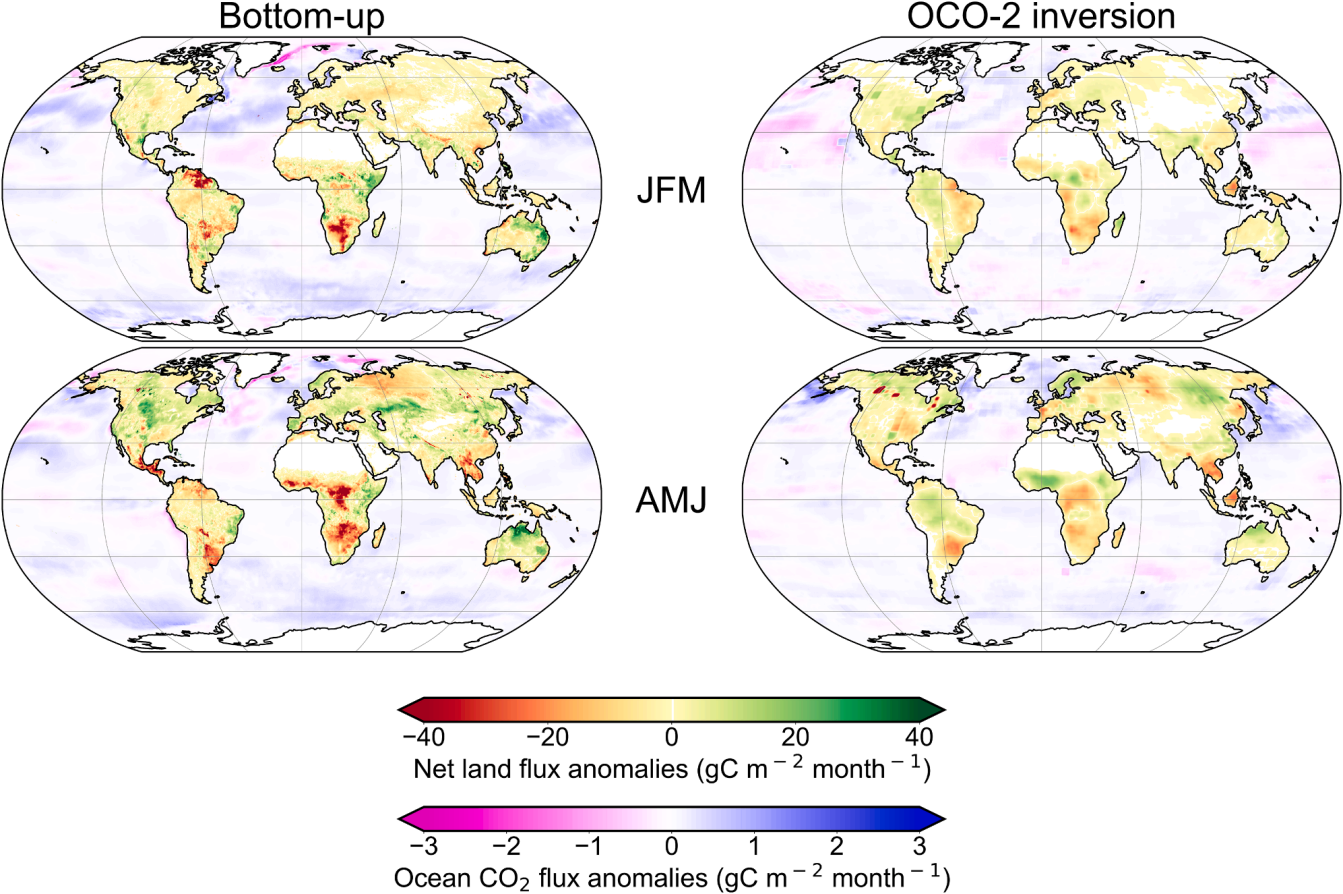
Net land and ocean CO_2_ flux anomalies for January to March (JFM) and April to June (AMJ) 2024 Anomalies are calculated relative to the 2015–2022 average of the DGVM models (left column, mean of four models) and the OCO-2 inversions (right column, mean of three models). Positive values represent anomalous CO_2_ uptake from the atmosphere by the land (green) or ocean (blue).

To gain insights on which regions caused the large global CO_2_ source anomaly in H1-2024, we analyzed the spatial patterns of flux anomalies for Jan-Mar (JFM) and Apr-Jun (AMJ) in the DGVM models and the inversions. The results are displayed in Figure 2, in Figure S4 with SiB4 and the DGVM emulators, and in Figures S5 and S6 for the JFM and AMJ mean fluxes over the RECCAP2 regions along with their distributions relative to the same quarters in previous years. The H1-2024 global land fluxes and key regional anomalies discussed in the following are summarized in Table 2, whereas the complete RECCAP2 land and ocean matrices are provided in Tables S1 and S2. A cross-dataset comparison of H1-2024 land CO_2_ flux anomalies is provided in Table S3 to clarify the roles of the primary and cross-check land-flux products. Table S3 shows that, in the tropics, the ERA5-driven DGVMs estimate a stronger H1-2024 source anomaly (−0.92 ± 0.17 GtC) than the OCO-2 inversions (−0.26 ± 0.04 GtC), whereas the 19-DGVM emulator ensemble (−0.34 ± 0.25 GtC) and SiB4 (−0.38 GtC) are closer to the inversion estimate. This supports the use of the emulators and SiB4 as independent cross-checks rather than central budget estimates, and indicates that the amplitude of the tropical source anomaly is more method-dependent than its sign.

Over the ocean, the most notable increases in carbon uptake were observed in the Pacific Ocean, consistent between the ocean models emulators and inversions (Figure 2). Particularly, increased ocean CO_2_ uptake was most pronounced in the eastern equatorial Pacific, consistent with suppressed upwelling of carbon rich waters during the developing El Niño.^37^ The carbon sinks in the Arctic Ocean, the Atlantic Ocean, the Indian Ocean, and coastal oceans remained relatively unchanged. There is a divergence in the Southern Ocean, where ocean model emulators suggest an increase, but the OCO-2 inversion indicates a decrease (Figures S6C–6E and Table S2). This modest ocean-sink strengthening should be interpreted in the context of recent studies showing that the ocean response to the 2023/24 warming was process- and region-dependent. Zhang et al.^44^ reported a weak decline in marine net primary production over 2003–2021 and emphasized the sensitivity of ocean photosynthesis to warming and ENSO variability; this result concerns biological production rather than the air-sea CO2 flux diagnosed in our budget. Müller et al.^45^ found that the 2023 non-polar ocean sink was weaker than expected from the historical relationship with global sea surface temperature (SST) anomalies, mainly because anomalous outgassing in subtropical and subpolar regions offset the El Niño-related reduction in tropical Pacific outgassing. Our result is therefore not directly inconsistent with these studies. The increased uptake diagnosed here is concentrated in the eastern equatorial Pacific and is evaluated for H1-2024 and the July-2023-to-June-2024 budget relative to the 2015–2022 same-calendar baseline, whereas Müller et al. assessed the calendar-year 2023 non-polar ocean sink relative to an SST-based expectation. Thus, our budget captures the regional El Niño reduction in tropical Pacific CO2 outgassing, while acknowledging that biological production changes and extratropical SST-driven outgassing can weaken the broader ocean sink response.

Over land, the regional net land CO_2_ flux anomalies in Figure 2 show their clearest dipole over the northern extratropics in AMJ-2024, with anomalous uptake over Eastern Europe, Central Asia and eastern Siberia opposed by anomalous sources over European Russia and Western Siberia, North America, and China. Both the DGVMs and the inversions capture this broad source-sink structure, but the inversions, especially CAMS, show sharper and larger regional contrasts (Figure S7).

Over the northern lands (north of 30°N), the net land CO_2_ flux anomaly was a source of 0.15 ± 0.25 GtC in the DGVMs and an uptake of 0.07 ± 0.06 GtC in the inversions during JFM-2024. The northern net land flux anomaly in JFM is within 1-sigma of the variation of previous JFM periods during 2015–2022. During AMJ-2024, the northern land net CO_2_ flux anomaly was 0.34 ± 0.07 GtC in the DGVMs (within 1-sigma of anomalies during 2015–2022) and 0.01 ± 0.09 GtC in the inversions (within 1-sigma of anomalies during 2015–2022). Such a large difference between DGVMs and inversions reflects differences for the spring and early summer northern CO_2_ uptake: unlike inversions, the DGVMs are not constrained by the observed northern CO_2_ atmospheric mixing ratio drawdown in AMJ. The strongest bottom-up/top-down divergence therefore occurs over the northern extratropics, especially in AMJ-2024, when the DGVMs, emulators and SiB4 indicate enhanced spring and early-summer uptake, whereas the inversions indicate nearly neutral anomalies. The inversions indicate an anomalous CO_2_ uptake in AMJ-2024 in Eastern Europe, Central Asia, eastern Siberia, and an anomalous CO_2_ source in European Russia and Western Siberia, North America, and China (Figure 2). The DGVMs flux anomalies are consistent with inversions over boreal North America, European Russia, and eastern Siberia.

In the second half of 2023 (H2-2023), we previously found anomalous sources of CO_2_ of 0.14 ± 0.05 GtC in the Amazon, 0.21 ± 0.07 GtC in central Africa, and 0.06 ± 0.03 GtC in tropical Asia, respectively, using as reference period the mean of all H2 periods during the period 2015–2022 covered by OCO-2 data. Updates of those regional CO_2_ flux anomalies are provided in this study for the period H1-2024. Over the Tropics, both DGVMs and inversions diagnose net land CO2 source anomalies during JFM and AMJ-2024, indicating agreement in sign and broad spatial organization; the main difference is in their amplitude (Figure 2). Over tropical South America, anomalous CO_2_ sources persisted during JFM-2024, but had a weaker magnitude than in late 2023 (see Figure 2 of Ke et al.^9^). The DGVMs and the inversions locate anomalous sources over eastern Brazil, and the DGVMs further show anomalous sources over Southern Brazil. The magnitude of the CO_2_ source anomaly over tropical South America north of 30°S is of 0.28 ± 0.15 GtC in the DGVMs and 0.03 ± 0.04 GtC in inversions during JFM-2024, smaller than the source anomalies during the preceding period of October-December 2023 analyzed in Ke et al.^9^ This smaller net land CO_2_ source anomaly is consistent with the fact that the drought in the Amazon peaked in late 2023 but diminished in JFM-2024 with only 7.5% of the region being under extreme drought (Figure 5). In AMJ-2024, the terminating El Niño conditions coincided with a return to net land CO_2_ sink anomalies, but a source anomaly developed in Southern Brazil, associated with low rainfall. In tropical Africa, we found a large source anomaly in the DGVMs and a small source anomaly in the inversions in AMJ-2024, following a source anomaly of 0.03 ± 0.14 GtC in the DGVMs and 0.09 ± 0.01 GtC in inversions during JFM-2024. This transition to anomalous CO_2_ sources in this region contrasts with the anomalous CO_2_ sink that prevailed during December 2022-February 2023 when conditions were wetter than normal.^9^ In Southern Africa, both JFM and AMJ-2024 were net land CO2 source anomalies, while the Horn of Africa remained wetter and was a CO_2_ sink anomaly. In tropical Asia and Oceania, both DGVMs and inversions diagnosed a net land CO_2_ source anomaly in AMJ-2024 mainly over Southern China, continental Southeast Asia, and Indonesia. In Northern Australia, despite continuing El Niño conditions usually associated with drought during the Australian summer and autumn, both DGVMs and inversions located an anomalous CO_2_ uptake during AMJ-2024. A similar pattern emerges across the rest of the tropics: the two approaches broadly agree on source anomalies in central and southern Africa and over southern China, continental Southeast Asia and Indonesia, and on anomalous uptake in northern Australia, while differing mainly in the magnitude of the tropical source anomalies.

**Figure 3:**
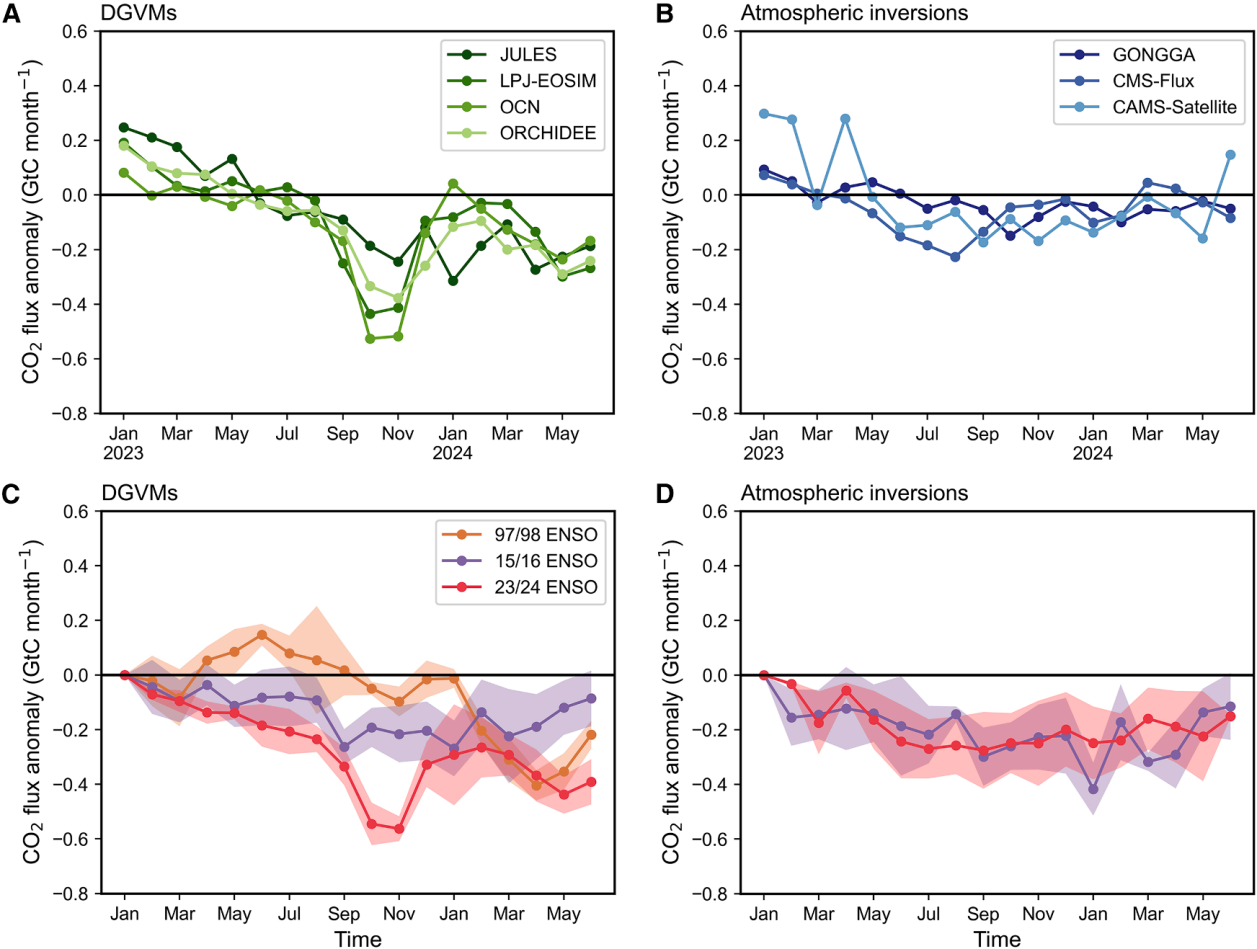
Evolution of monthly tropical CO_2_ flux anomalies (A) Anomalies from the four DGVMs used in this study, starting from January 2023, showing a switch to CO_2_ source anomalies denoted by negative values starting in September 2023 and lasting until at least July 2024. (B) Same for the three inversions. (C) Comparison of mean CO_2_ flux anomalies and 1-sigma standard deviation between models (shaded areas) of the four DGVMs for the last three El Niño events (1997/98, 2015/16, and 2023/24). (D) Same for the three inversions which start in 2015 and therefore do not cover the 1997/98 event.

The monthly evolution of net land CO_2_ flux anomalies in the tropics is displayed in Figure 3A for the DGVMs and in Figure 3B for the inversions. Figure 3 confirms that both approaches capture persistent tropical source anomalies from mid-2023 onward, but the DGVMs simulate a sharper onset and a larger cumulative anomaly than the inversions. The results indicate that the DGVMs show a sharp transition to a CO_2_ source anomaly in August 2023 with a maximum loss anomaly in November 2023, mainly from the Amazon, a return to weaker anomalies in JFM-2024, and an emerging CO_2_ source anomaly thereafter. JULES, ORCHIDEE-MICT, LPJ-EOSIM and OCN show similar timing of the tropical source anomaly, with the strongest negative anomalies during late 2023 (Figure 3A). The inversions show earlier but smaller monthly net land CO_2_ source anomalies than the DGVMs, starting in June 2023 and persisting until July 2024. The cumulative CO_2_ source anomaly from June 1^st^ 2023 to July 1^st^ 2024 is 1.55 ± 0.13 GtC yr^−1^ in the inversions compared to 2.45 ± 0.50 GtC yr^−1^ in the DGVMs, suggesting that the DGVMs overestimated CO_2_ losses during that period, possibly because they overestimate the response of photosynthesis to drought or because they ignore the transient storage in deadwood pools after drought induced mortality,^46^ as discussed in the section on model limitations later in discussion.

### Regional flux anomalies in relation to land water storage

**Figure 4:**
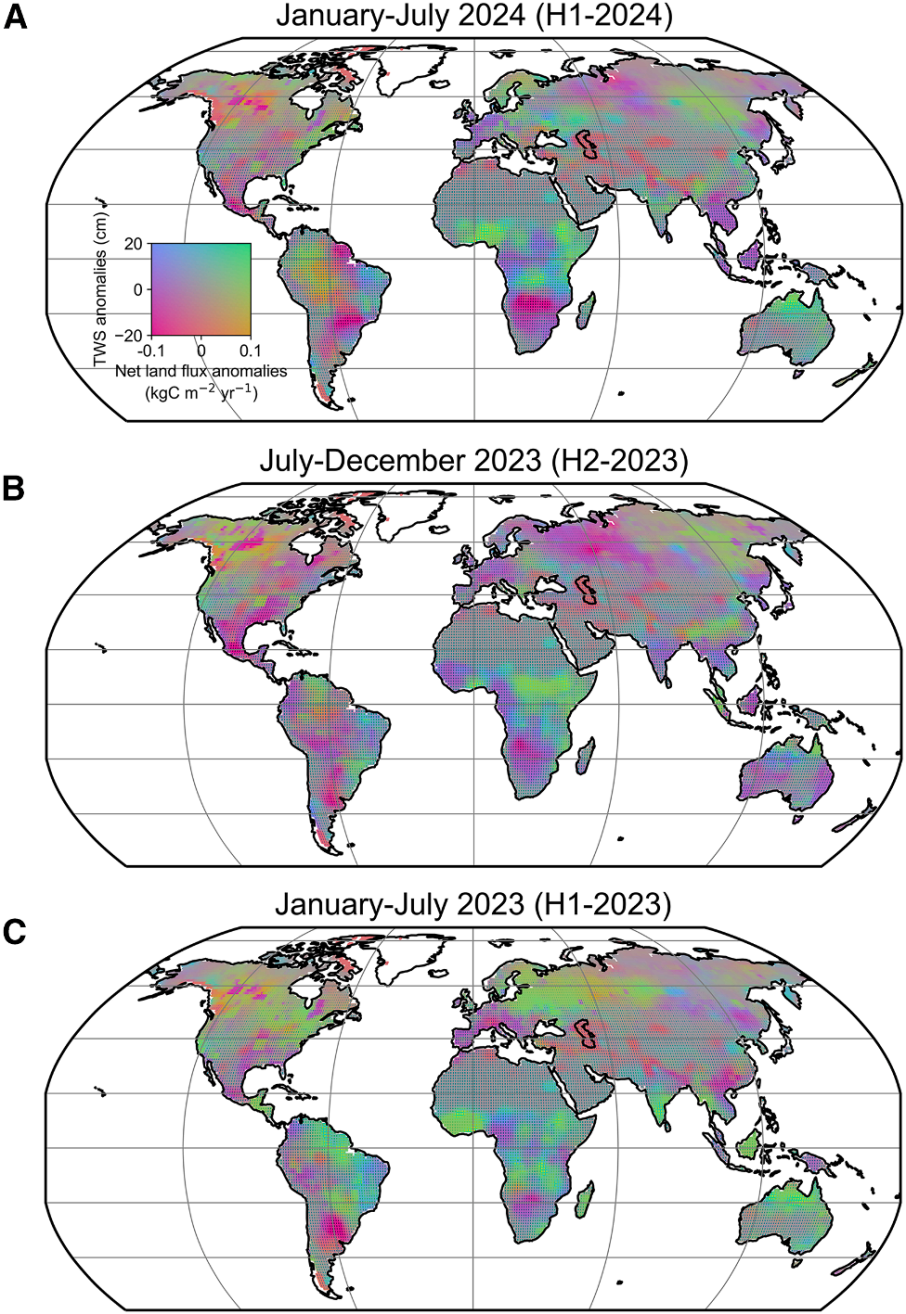
Net land flux anomalies covary with total water storage anomalies (A) Bivariate plot showing covariations between net land flux anomalies from inversions and total water storage anomalies from the GRACE satellites for H1-2024. (B) Bivariate plot showing covariations between net land flux anomalies from inversions and total water storage anomalies from the GRACE satellites for H2-2023. (C) Bivariate plot showing covariations between net land flux anomalies from inversions and total water storage anomalies from the GRACE satellites for H1-2023. Green areas show wetter anomalies coincident with more CO2 uptake, and magenta areas show drier anomalies coincident with reduced CO2 uptake.

The bivariate anomalies of net land CO_2_ fluxes and total land water storage from the global gridded daily CO2 emission (GRACE) satellites, assumed to represent soil water availability for plants, during each half-year period since Jan 1^st^ 2023 are shown in Figures 4 and S10. There is a shift between H1-2023 and H2-2023 toward drier anomalies associated with less CO_2_ uptake or increased CO_2_ sources (magenta areas in Figures 4B and 4C). From H1-2023 to H2-2023, we found a large increase in the extent of those types of dryer/CO_2_ anomalous source anomalies from 23.0% to 37.2%, and then a decrease to 28.8% of the global land area from H2-2023 to H1-2024. In the northern extratropics, drier conditions associated with decreased CO_2_ uptake prevailed in North America, Central Asia, and Western Siberia. In the Tropics, we observe a reduction of dryer/decreased CO_2_ uptake anomalies over the Amazon in H1-2024 during the termination of the El Niño. However, the decrease in CO_2_ uptake in Southern Brazil, which has been affected by a long-term drought, persists. We also observe a shift toward dryer/decreased CO_2_ uptake in central Africa during H1-2024, as well as a persistence of drought in the Southern part of Africa. Eastern Africa remained under a regime of wetter/more CO_2_ uptake between H2-2023 and H1-2024. In Asia and Oceania, the data in Figures 4A and 4B indicate CO_2_ losses from inversions, yet associated with wetter conditions from GRACE. Northern Australia remained characterized by wetter/higher CO_2_ uptake conditions.

### Comparison of the 2023/24 El Niño with the two previous episodes

**Figure 5:**
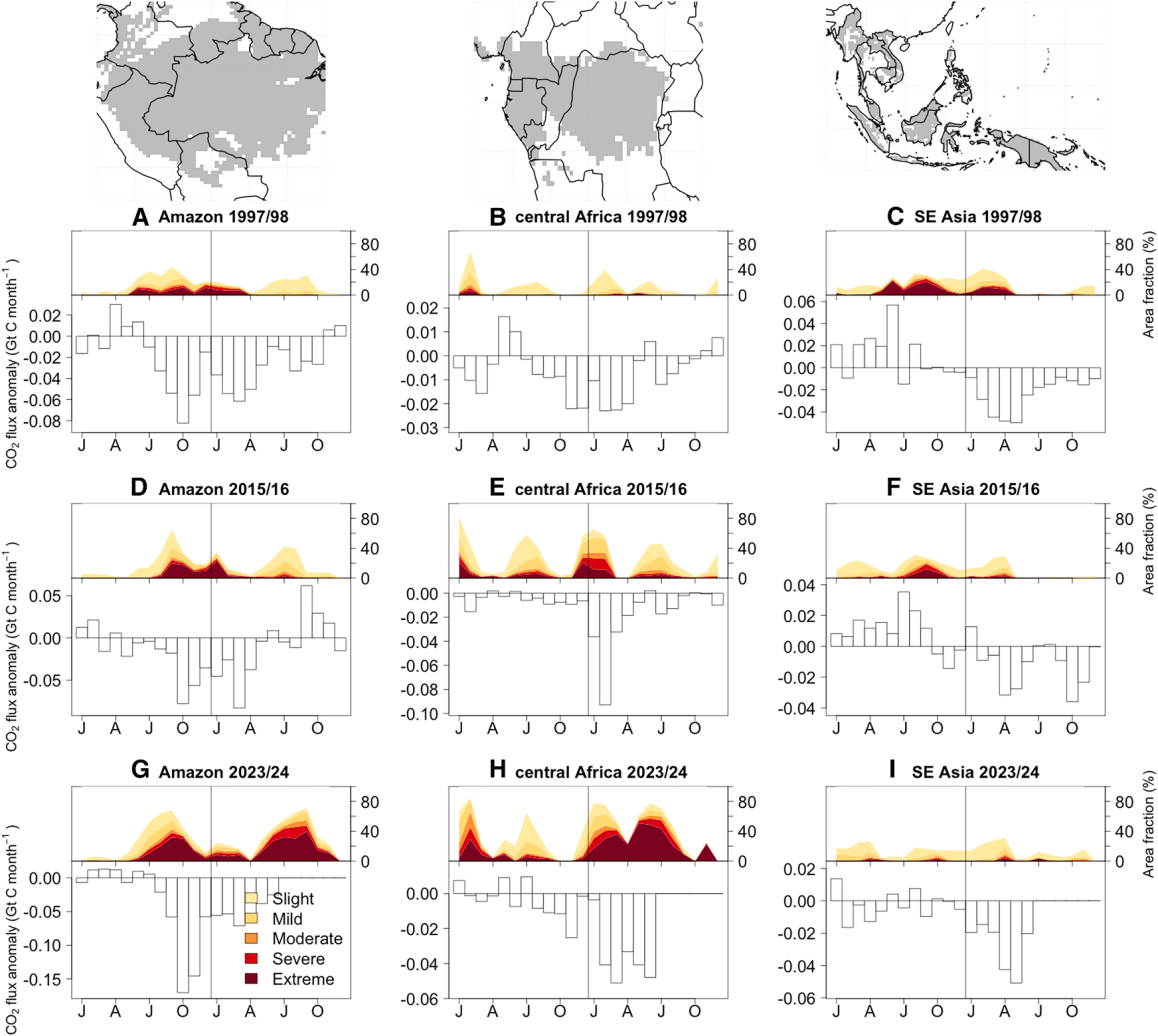
Drought timing and extent of the three El Niño events with net CO_2_ flux anomalies The upper part of each subplot displays the fraction of each tropical continental area in gray under drought for different intensities. Fractional area under each drought severity category is calculated for different severities based on *Z* score transformed monthly cumulative water deficits. The lower part displays monthly net land CO_2_ flux anomalies from the mean of four DGVMs (ORCHIDEE-MICT, OCN, LPJ-EOSIM and JULES) calculated relative to the 5-year pre-event baseline for each El Niño. (A–C) For the 1997/98 El Niño in the Amazon, central Africa and tropical Southeast Asia. (D–F) Same for the 2015/16 El Niño. (G–I) Same for the 2023/24 El Niño. The anomalies for this El Niño are only simulated until July 2024.

We further analyzed the development of monthly water deficit anomalies and their impact on net land CO_2_ fluxes for each tropical continent during the first and second years of the last three El Niño events in 1997/98, 2015/16 and 2023/24. The results displayed in Figure 5 show that in the Amazon, water deficits during El Niño years usually develop during the dry season of the first El Nîño year, peak around January, and decrease in the second El Niño year. From MEI index values, El Niño conditions typically terminate by April or May of the second year.^7^ In Southeast Asia, El Niño drought is synchronous with the Amazon. In central Africa, there is not necessarily a strong immediate teleconnection between El Niño and rainfall deficits, although El Niño years tend to be followed by drier conditions. The delayed or weak response of African rainfall during El Niño years is due to interactions with Indian Ocean warming, seasonal effects, and atmospheric wave propagation.^47^

During the 1997/98 El Niño, water deficits started in June 1997 and ended in April 1998, with a peak in September 1997 corresponding to a maximum of 43% of the Amazon region being under drought and 12% under extreme drought (see STAR Methods for drought severity definitions based on water deficits). In Central Africa, there are two periodical dry seasons in Dec-Feb and Jun-Oct, respectively (Figure 5). In 1997/98, Central African forests were only moderately affected by drought, with only 7.6% of their area under mild drought at peak and no severe drought conditions. The 2015/2016 El Niño was more severe, and the area under drought in the Amazon was higher than in 1997/98, with 24% of the area being under extreme drought in Jan 2016, although the total duration of the water deficit period was shorter than in 1997/98 by 3 months. In Central Africa, the two dry seasons had clearly more severe droughts than in 1997/98, with up to 21.6% of the area being under extreme drought in the dry season of DJF 2016. In 2023/24, the El Niño event of interest in this study, which has never been analyzed before, can be see in Figure 5; the drought was much more severe than the two previous El Niño episodes. In the Amazon, there was a peak of areas under water deficit of 68.2% during the record drought of Sep-Dec 2023.^8^^,^^48^^,^^49^^,^^50^ The 2023/24 El Niño drought was severe during the second El Niño year in 2024 compared to previous second years in 1998 and 2016, and drought conditions restarted in June 2024 after the end of the El Niño (Figure 5).

Figure 5 also shows anomalies of the net land CO_2_ fluxes from the mean of the DGVMs during the last three El Niños. In general, the models simulated negative flux anomalies (anomalous losses, or reduced sinks) both during the onset of El Niño in the first year and its decay in the second year. Anomalous CO_2_ losses are systematically more severe in the first year than in the second year over the Amazon, with a peak source anomaly during October when the usual dry season coincides with the developing water deficit from El Niño. Generally, anomalous CO_2_ losses are terminated in the Amazon by April of the second El Niño year. During the full El Niño periods of 1997/98, the DGVMs give an anomaly loss of CO_2_ of 0.56 ± 0.42 GtC yr^−1^ across the pan-tropics, an anomalous loss of 0.56 ± 0.20 GtC yr^−1^ in the Amazon, an anomalous uptake of 0.26 ± 0.06 GtC yr^−1^ in Central Africa and an anomalous loss of 0.28 ± 0.14 GtC yr^−1^ in South-east Asia (Figure 5). In 2015/16, the anomalous pan-tropical loss was larger than in 1997/98, with a value of 1.58 ± 0.03 GtC yr^−1^, including anomalous losses of 0.55 ± 0.06 GtC yr^−1^ in the Amazon, 0.41 ± 0.03 GtC yr^−1^ in Central Africa and 0.29 ± 0.06 GtC yr^−1^ in Southeast Asia, respectively. In 2023/24, the pan-tropical loss reached up to 2.31 ± 0.14 GtC yr^−1^, with a loss of 1.51 ± 0.13 GtC yr^−1^ in the Amazon, 0.55 ± 0.12 GtC yr^−1^ in Central Africa, and 0.11 ± 0.03 GtC yr^−1^ in Southeast Asia, respectively. This result from DGVMs, suggesting a more severe CO_2_ loss anomaly during the 2023/24 event compared to the two previous events in 1997/98 and 2015/16 is not corroborated by inversion results in Figures 3C and 3D, which tend to show a similar CO_2_ loss anomaly between the 2023/24 event and the 2015/16 El Niños. Note that the OCO-2 inversions starting in 2015 do not cover the 1997/98 El Niño.

This comparison should therefore be interpreted cautiously. In the ERA5-driven DGVMs, the 2023/24 event appears stronger than the 1997/98 and 2015/16 events, whereas the OCO-2 inversions indicate a 2023/24 tropical source anomaly more comparable to 2015/16. Thus, the ranking of the 2023/24 event relative to previous El Niño events is not identical across approaches. Fire emissions amplified tropical source anomalies during drought-affected periods, but the larger 2023/24 DGVM anomaly should not be interpreted as a fire-only signal. A diagnostic separation between fire and non-fire anomalies for the two events with comparable pre-event fire baselines, 2015/16 and 2023/24, shows that fire anomalies account for about 4.8% of the pan-tropical source anomaly in 2023/24 and about 13.9% in 2015/16. Regionally, fire contributions are small in Latin America north of 30°S and tropical Africa, whereas Southeast Asia shows a stronger fire contribution in 2015/16 than in 2023/24. The remaining source anomaly is therefore mainly associated with non-fire ecosystem fluxes. This interpretation is consistent with the component analysis in Figure S12, which shows that the 2023/24 weakening of net land uptake was primarily associated with a negative GPP anomaly, while Ra and Rh anomalies were smaller.

Fire emissions anomalies were particularly severe in the south-central Africa and Amazon region, closely aligned with periods of intense drought described above (Figure S11). During H2-2023, extensive drought conditions in the Amazon led to significant wildfire emissions, with unprecedented carbon emissions particularly noted in Brazil and Venezuela. Fire emissions further increased into H1-2024, resulting in record-breaking wildfire emission anomalies (0.06 GtC relative to 2015–2022 average) across northern South America, notably Bolivia, Guyana, and Suriname, with Bolivia recording its highest fire emissions since 2003. In contrast, Southeast Asia and Central Africa exhibited relatively modest fire emission anomalies during the same period. Southeast Asia, in particular, saw fire emissions remaining stable or even below average levels in H1-2024, suggesting effective regional fire management or milder climatic conditions. Central Africa similarly displayed relatively stable fire emission patterns throughout the 2023/24 El Niño event, consistent with the less pronounced drought anomalies discussed previously.^51^

### Agreement between models and model limitations

The DGVMs and inversions show limited grid-cell-scale agreement in the spatial pattern of net land CO_2_ flux anomalies during JFM (R^2^ = 0.034 at 1 ° × 1 ° resolution), but their agreement improves in AMJ (R^2^ = 0.20), when both approaches capture a clearer northern extratropical source-sink contrast (Figure 2). Here, the inversion fluxes for GONGGA and CMS-flux have been re-interpolated to 1° × 1° from their coarser native resolution. We also analyzed the level of agreement between the different models used in this study (Figure S7). The level of agreement between the DGVMs in each 1° grid cell is defined as good if the four models have a JFM or AMJ flux anomaly higher than 1-sigma from the 2015–2022 period in this grid cell and its magnitude differs by less than 30%. We found that 80.24% and 80.71% of the northern lands and 68.84% and 88.94% of the tropics have a good agreement for the JFM and AMJ of 2024. At a larger scale of continents, the agreement of flux anomalies was also good between the models. The level of agreement between the three inversions was considered good if 2 models out of three have a flux anomaly that differs by less than 20%. At the scale of the coarsest inversion model, CMS-Flux (with a resolution of 4 ° × 5 °), the agreement of inversions over the northern lands was 90.44% and 83.97% for JFM and AMJ of 2024, respectively. Similarly, over the tropical region, inversion agreement reached 86.54% in JFM and 83.64% in AMJ, indicating consistently high levels of agreement across different latitude bands.

A main limitation of the DGVM models used in this study for studying the response of CO_2_ fluxes to drought is that they have no drought induced tree mortality parameterization, a limitation common to most other DGVMs. DGVMs simulate a CO_2_ loss from a deficit in gross primary productivity caused by water stress, and generally smaller changes in plant and soil CO_2_ respiration since warmer conditions increase respiration rates, while dryer conditions and coincident drought-induced decreases of GPP reduce them.^52^ Therefore, even though the net land CO_2_ flux anomalies during 2023/24 simulated by our DGVMs are qualitatively consistent with the independent anomalies from inversions, DGVM models do not simulate lagged mortality processes as observed in the Amazon and in other tropical wet forest biomes.^53^^,^^54^^,^^55^ Increased mortality also causes a transient increase of coarse woody debris on the forest floor, which will not be decomposed immediately and will cause a temporary additional carbon storage, typically during 20 years,^46^^,^^56^ with a lagged release of CO_2_ to the atmosphere, contributing to a higher atmospheric CO_2_ growth rate in the following decades. Since DGVMs do not simulate increased tree mortality during drought followed by transient storage in deadwood, this important process controlling CO_2_ fluxes is absent from our budget assessment, which may explain why the DGVMs' CO_2_ source anomaly in 2023/24 is larger than that of inversions, as noted by Yang et al.^46^ These missing processes also affect the interpretation of differences among individual El Niño events. The DGVMs mainly represent the immediate response of photosynthesis, respiration, and fire emissions to drought, whereas drought-induced mortality and the subsequent decomposition of coarse woody debris can redistribute part of the carbon loss over years to decades. As a result, the DGVM-based event-year anomalies should be interpreted as estimates of the immediate ecosystem-exchange response to drought and fire, rather than as a complete representation of all delayed carbon losses triggered by each El Niño event. Last, our DGVMs were corrected for fire emissions using data-driven emission products based on moderate-resolution sensors that underestimate small degradation fires in wet tropical forests.^57^ It is expected that new burned area products with more small fires^58^ will increase emissions when emissions datasets are available from those products.

Despite these limitations related to mortality representation and fire emissions uncertainties, we further investigated the robustness and ecological plausibility of our DGVM-derived net land CO_2_ flux anomalies NEE by explicitly examining the half-year anomalies of the individual terrestrial carbon flux components—GPP, autotrophic respiration (Ra), and heterotrophic respiration (Rh) (Figure S12). The analysis reveals that the pronounced reduction in global terrestrial carbon uptake during the 2023/24 El Niño event was predominantly driven by a large negative anomaly in GPP, reflecting reduced vegetation productivity under drought stress conditions. By comparison, the anomalies observed in Ra and Rh were significantly smaller, indicating that the simulated weakening of the land carbon sink primarily originates from reduced photosynthesis rather than increased ecosystem respiration. This result strengthens our confidence in the modeled NEE anomalies, despite the acknowledged limitations regarding drought-induced tree mortality and transient storage effects discussed above.

The choice of meteorological forcing datasets also contributes to the spread in modeled terrestrial carbon fluxes, especially through differences in tropical precipitation during the 2023/24 El Niño event. To provide a quantitative diagnostic, we compared ERA5 and CRU-JRA precipitation anomalies over the main tropical source regions (Table S4). ERA5 precipitation anomalies are more negative than CRU-JRA over several key regions, including Latin America north of 30°S during JFM-2024 (−23.3 mm month^−1^ in ERA5 versus −2.5 mm month^−1^ in CRU-JRA) and tropical Africa during AMJ-2024 (−11.9 versus −0.6 mm month^−1^). At the pan-tropical scale, ERA5 also gives more negative precipitation anomalies than CRU-JRA during both JFM and AMJ 2024. In Southeast Asia, however, ERA5 is wetter than CRU-JRA, especially during AMJ-2024, showing that the forcing differences are region-dependent rather than systematic everywhere. Together with the cross-dataset flux comparison in Table S3, these precipitation diagnostics support the interpretation that part of the stronger tropical source anomaly in the ERA5-driven DGVMs may be related to meteorological forcing differences. They do not isolate the full forcing uncertainty, and structural differences in model drought sensitivity and carbon-cycle processes remain additional contributors to the DGVM-inversion discrepancy.

The three inversions used different priors and transport models, and different aggregations of OCO-2 observations into atmospheric model grid boxes. The spread of the inversions is of 0.51 GtC yr^−1^ for global CO_2_ fluxes during H1-2024, indicating that the budget over only six months is relatively well constrained by OCO-2 observations. The spread of inversions was 0.28 GtC for the ocean sink and 0.43 GtC for the land sink during H1-2024, which is larger than the spread of their annual land sinks from July 2023 to July 2024, but smaller than the spread of their annual ocean sinks. This indicates that inversions have a higher uncertainty when land budgets are analyzed on a semester basis. The spread of our inversions is also lower than that of the four DGVM models. We found that the three inversions agree about the sign of tropical flux anomalies in each continent, and on the spatial patterns of flux anomalies during H1-2024 (spatial R^2^ = 0.05 for JFM and 0.01 for AMJ between the three models at 4° × 5° resolution, the resolution of the coarsest inversion—CMS-Flux). However, the magnitude of regional anomalies in CAMS is twice as large as in CMS-Flux and GONGGA, which reflects its higher spatial resolution. This difference needs to be investigated in further studies.

### An audit of our latency models against multi-model ensembles from the global carbon budget in 2023

In this study, to be consistent with GCB2024, we only use the growth rate from MBL stations. In our previous low-latency budget,^9^ we used the growth rate from MBL stations as in the previous global carbon budget assessment (GCB2024) but also from the MLO station. The growth rate at MLO was 0.37 ppm yr^−1^ higher than the MBL stations from June-July 2023 to June-July 2024 according to NOAA Global Monitoring Laboratory data (Figure S1B). If the budget imbalance from July 2023 to July 2024 were calculated using the MLO station, the imbalance would be −0.70 GtC yr^−1^ instead of 0.09 GtC yr^−1^ with MBL data, implying a missing source in our bottom-up budget instead of a small missing sink with MBL data (Figure S1C). The monthly MBL minus MLO growth rate difference took its largest negative value of 1.14 ppm yr^−1^ in February 2024 (Figure S1A). This persistent and intriguing positive difference between MLO and MBL growth rates could be due to changes in emissions and regional atmospheric transport impacting MLO, or possibly due to volcanic activity at MLO between Dec. 2022 and July 2023 that resulted in moving the instrument to nearby Mauna Kea. Cumulatively, over a long enough time, the difference of atmospheric CO_2_ increase eventually cancels out between MLO and MBL, as shown above.

For this second edition of the low latency global and regional CO_2_ budget, we benchmarked our previous 2023 results, reported in Ke et al.^9^ and based on the three low-latency DGVMs (JULES, OCN, ORCHIDEE-MICT) together with the CAMS inversion, against the mean of all the GCB2024 DGVMs and inversions in Friedlingstein et al.^16^ Note that the net land CO_2_ flux of each DGVM was simulated from climate forcing only but adjusted to the mean of the TRENDY models S3 simulation in the period 2019–2022 which includes land use change. Therefore, our models include the average flux from land use change implicitly but ignore recent variations of land use change in 2023 and 2024. The results of the comparison are shown in Figures S9A and S9B for the DGVMs and inversion estimates of the net land CO_2_ flux, and in Figures S9C and S9D for ocean model emulators and inversions over the ocean. The CAMS inversion that was used in Ke et al.^9^ is very close to the median of the 8 satellite and 6 *in situ* inversions used by GCB2024. On the other hand, the mean of the three DGVMs used by Ke et al.^9^ (JULES, ORCHIDEE-MICT, OCN) shows a lower net land CO_2_ flux than the mean of the 20 DGVMs from GCB2024, by 1.07 GtC yr^−1^. Our lower DGVM net land flux is still in the range of GCB2024, with 6 models producing sinks of the same magnitude as the three DGVMs of Ke et al.^9^ Values of global and regional anomalies of land CO_2_ fluxes are shown in Table S1. For the ocean sink, which has less variability from one year to another than the land CO_2_ sink, the results of our 16 emulators are extremely close to those of ocean biogeochemistry models and data-driven models used by GCB2024. Similarly, the results of the CAMS inversion for the ocean sink in 2023 are very close to the mean of all the inversions used by GCB2024.

### DGVM emulators to predict land CO_2_ fluxes in the first half of 2024

**Figure 6:**
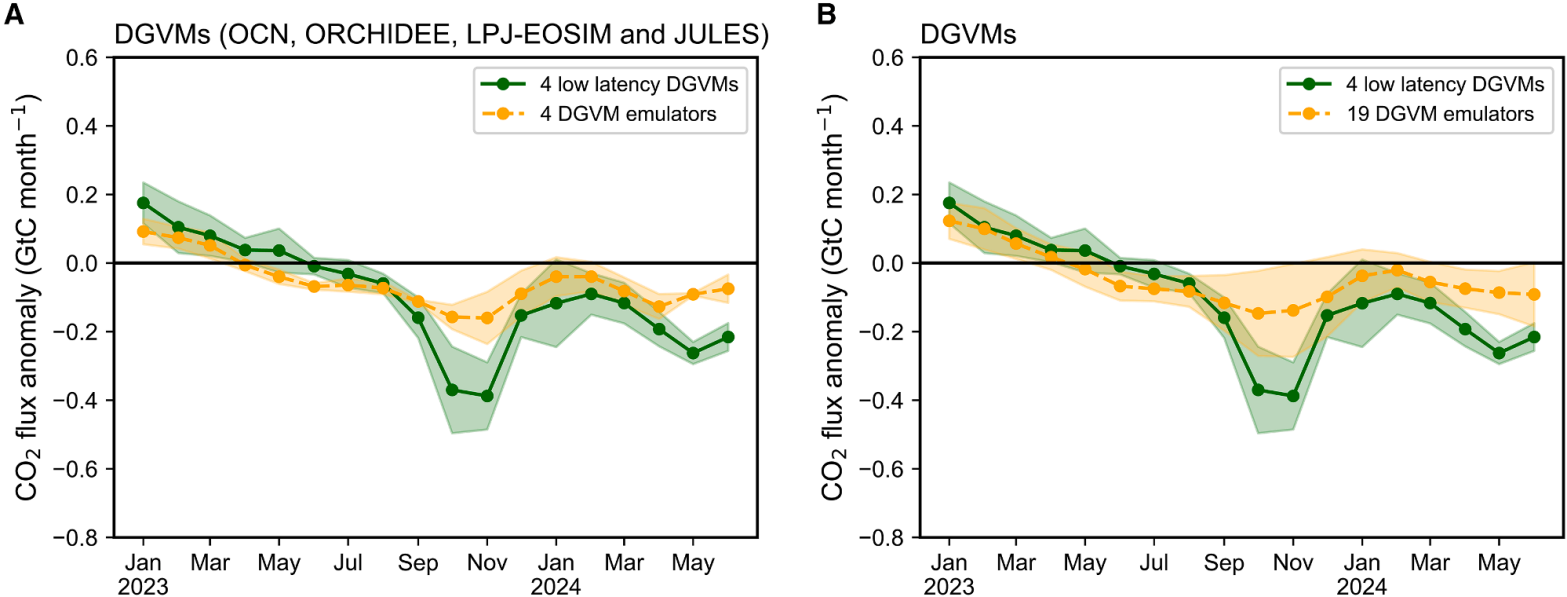
Comparison of monthly tropical CO_2_ flux anomalies from January 2023 to July 2024 between ML emulators of DGVMs and the four DGVMs used in this study (A) Comparison of ML emulators (yellow) and original simulations (green) for the same four DGVMs used in this study (OCN, ORCHIDEE, LPJ-EOSIM and JULES). The emulators are trained using runs of each DGVM until 2023 provided for the global carbon budget (GCB2024).^16^ The ORCHIDEE and LPJ-wsl models used in GCB2024 are different from the ORCHIDEE-MICT and LPJ-EOSIM versions used in this study. (B) Comparison of the four low-latency DGVMs (green) used in this study with the emulators (yellow) of 19 DGVMs from GCB2024 with monthly outputs. Shaded areas indicate one standard deviation above and below the ensemble mean in both panels.

In this study, for the first time, we developed 19 ML emulators of DGVMs used in GCB2024. These emulators are deep learning models trained to simulate the monthly net land sink from simulation 2 of each original GCB2024 DGVM at 0.5 × 0.5° resolution. The emulators predicted fluxes and flux anomalies at 0.5° resolution that closely match the original models during years not included in the training set. At this fine spatial resolution, grid-cell-level performance shows relatively modest spatial R^2^ values ranging from 0.08 to 0.35 across different quarters and models. However, despite these modest R^2^ values at individual grid cells, the emulators effectively capture over 90% of the variance in carbon flux anomalies at larger regional-to-continental scales (see flux anomaly maps for four GCB2024 models during test years in Figure S3). In their prediction for the H1-2024 period, the mean of the emulators gives a global sink of 2.05 ± 0.37 GtC and a source anomaly of 0.21 ± 0.33 GtC. The monthly anomalies of tropical net land CO_2_ fluxes from the mean of the 19 emulators during H1-2024 are given in Figure 6. The result shown in Figure 6A indicates that the emulators of the four DGVM models used in this study, ORCHIDEE, OCN, JULES and LPJ-wsl, give a similar temporal evolution of tropical CO_2_ flux anomalies, but smaller anomalous CO_2_ losses than the low-latency native models. This difference is possibly due to the fact that the GCB2024 models used to train emulators were run with the CRU JRA atmospheric forcing, whereas our four low-latency DGVMs shown in Figure 6 were run using the ERA5 forcing. Figure 6B displays the monthly net land CO_2_ flux anomalies from the 19 emulators (mean and standard deviation). The results show that the mean of the 19 emulators has a smaller loss anomaly than our four low-latency DGVMs, and that the mean of the emulators is better aligned with inversions (Figure 3B). The performance of the emulators will continue to be assessed at least for one year against the results of each native DGVM in the next edition of the global budget. Then, emulators will be used as part of the next edition of our low-latency assessment of the CO_2_ budget.

Overall, we found a record-high CO_2_ growth rate from June-July 2023 to June-July 2024 associated with a slight increase of the ocean sink and a strong reduction of the net land CO_2_ flux to a value close to no net land CO_2_ source (0.09 ± 0.26 GtC yr^−1^) in the inversions, which provide fluxes that match the observed CO_2_ growth rate. Our four low-latency DGVMs give a larger net land CO_2_ source of 0.59 ± 0.60 GtC yr^−1^. In the inversions, the net tropical land CO_2_ source anomaly from July 2023 to July 2024 was 0.85 ± 0.08 GtC yr^−1^, compared to 2.15 ± 0.14 GtC yr^−1^ in DGVMs. In the inversions, the 2023/24 net land CO_2_ source anomaly was similar in magnitude to the one diagnosed for the previous El Niño of 2015/16, while the 2023/24 source anomaly was more severe in the DGVMs. Our DGVM results indicate that the Amazon experienced a less severe drought-induced CO_2_ loss in H1-2024 compared to the previous half year of H2-2023, while central Africa had a very severe drought after April 2024 during the second dry season in this region, causing new CO_2_ losses. Continental and maritime Southeast Asia also showed a CO_2_ source anomaly in the DGVMs despite a relatively moderate drought in H1-2024. We tested for the first time ML emulators of the 19 DGVMs used in the global carbon budget trained to reproduce the fluxes of the native models until December 2023 and used for predicting the land CO2 fluxes during H1-2024. The mean of the emulators gives a net tropical land CO_2_ source anomaly close to the one simulated by inversions, which gives us confidence to use emulators in subsequent assessments. It is interesting to see that the Amazon experienced a second extreme drought during the dry season in H2-2024 while central Africa continued to be under drought, an important event that will be the focus of the following low-latency budget assessment.

### Limitations of the study

This low-latency budget provides an early assessment of carbon cycle anomalies and should not be interpreted as a replacement for consolidated annual global carbon budget products. The magnitude of the land flux anomaly remains method dependent, with ERA5-driven DGVMs producing stronger tropical source anomalies than OCO-2 inversions, SiB4, and the DGVM emulator ensemble. DGVMs do not explicitly represent drought-induced tree mortality, delayed decomposition of coarse woody debris, or all degradation fires, which can redistribute carbon losses over years to decades. Fire emission products may also underestimate small fires in tropical forests. Atmospheric inversions are affected by transport errors, prior flux assumptions, spatial resolution, and shorter-term uncertainty, especially for regional half-year budgets. These limitations mainly affect the magnitude and ranking of regional anomalies, whereas the sign of the tropical land-sink weakening is more consistent across approaches.

## Resource availability

### Lead contact

Requests for further information and resources should be directed to and will be fulfilled by the lead contact, Piyu Ke (kepiyu@gmail.com).

### Materials availability

This study did not generate new unique materials.

### Data and code availability

#### Data

The datasets used in this study are publicly available from the following sources: Global Carbon Budget 2024 at www.icos-cp.eu/science-and-impact/global-carbon-budget/2024; OCO-2 retrievals at disc.gsfc.nasa.gov/datasets?page=1&keywords=OCO-2; NOAA/GML atmospheric CO_2_ data at https://gml.noaa.gov/ccgg/trends/; Carbon Monitor fossil-fuel emissions data at carbonmonitor.org; GFED4.1s fire-emissions data at geo.vu.nl/∼gwerf/GFED/GFED4/; GFAS fire-emissions data at atmosphere.copernicus.eu/global-fire-monitoring/; ERA5 monthly averaged data at cds.climate.copernicus.eu/cdsapp#!/dataset/reanalysis-era5-single-levels-monthly-means?tab=overview; the Multivariate ENSO Index at www.psl.noaa.gov/enso/mei; and GRACE/GRACE-FO terrestrial water-storage data at www2.csr.utexas.edu/grace/RL06_mascons.html.

#### Code

The analysis code used in this study is available from the lead contact upon reasonable request.

#### Additional information

Any additional information required to reanalyze the data reported in this paper is available from the lead contact upon reasonable request.

## Acknowledgments

We acknowledge the Global Carbon Project, which is responsible for the Global Carbon Budget. We thank the ocean modeling and fCO_2_-mapping groups for producing and making available their model and fCO_2_-product outputs, and the land modeling groups for producing and making available their model outputs. We also thank the ICOS Carbon Portal (ICOS ERIC) for providing access to global carbon budget datasets. P.C., F.C., A.B., S.S., and P.F. acknowledge support from the European Space Agency Climate Space RECCAP2-CS project (ESA ESRIN/4000144908), ESA Carbon-RO (4000140982/23/I-EF) and the CALIPSO (Carbon Loss in Plant Soils and Oceans) project funded by the generosity of Schmidt Science. P.C. and G.P.P. received funding from the European Union’s Horizon Europe Research and Innovation program under (grant no. 101081395) (EYE-CLIMA). The CAMS simulations were granted access to the HPC resources of TGCC under the allocation (grant no. A0170102201) made by GENCI. A.B. thanks Evgenii Churiulin for support in setting up the OCN model simulations. Work of J.L. and S.P. was conducted at the Jet Propulsion Laboratory, California Institute of Technology, under a contract with the National Aeronautics and Space Administration (grant no. 80NM0018D0004). Additionally, J.L. acknowledges the funding support from the NASA Orbiting Carbon Observatory Science Team program. AvdW acknowledges Remco de Kok, Ingrid Luijkx, and Joram Hooghiem for their contributions to CTE-HR and the ICOS-CP for hosting CTE-HR data. This work was also supported by a computing grant from the Dutch national e-infrastructure with the support of the SURF Cooperative (grant no. NWO-2023.003). This research was supported in part by the NOAA cooperative agreement NA22OAR4320151. The statements, findings, conclusions, and recommendations are those of the author(s) and do not necessarily reflect the views of NOAA or the U.S. Department of Commerce.

## Author contributions

P.K. and P.C.: designed the research; P.K. and P.C.: performed the analysis; P.K., P.C., and Y.Y.: collected and analyzed the research data; P.C. and P.K.: created the first draft of the paper; all authors contributed to the interpretation of the results and to the text.

## Declaration of interests

The authors declare no competing interests.

## STAR★Methods

### Key resources table

**Table:** 

REAGENT or RESOURCE	SOURCE	IDENTIFIER
**Deposited data**		

Global Carbon Budget 2024 data supplement	Global Carbon Project; Friedlingstein et al.^16^	Version 1.0 https://doi.org/10.18160/GCP-2024
Global Carbon Budget 2023 data supplement and TRENDY simulation outputs used for calibration	Global Carbon Project; Friedlingstein et al.^25^	https://doi.org/10.18160/GCP-2023
Global Carbon Budget 2022 ocean biogeochemical-model and data-product outputs used to train the ocean emulators	Global Carbon Project; Friedlingstein et al.^30^	https://doi.org/10.18160/GCP-2022
OCO-2 Level 2 Lite bias-corrected XCO_2_ retrievals	NASA/JPL and NASA GES DISC; Payne et al.^38^	OCO2_L2_Lite_FP, retrospective processing V11.2r https://doi.org/10.5067/70K2B2W8MNGY
NOAA globally averaged marine boundary layer atmospheric CO_2_ record	NOAA Global Monitoring Laboratory; Lan et al.^6^	https://doi.org/10.15138/9N0H-ZH07 https://gml.noaa.gov/ccgg/trends/gl_data.html
NOAA Mauna Loa atmospheric CO_2_ record	NOAA Global Monitoring Laboratory; Lan and Keeling^5^	https://gml.noaa.gov/ccgg/trends/data.html
Carbon Monitor near-real-time daily fossil-fuel and cement CO_2_ emissions	Liu et al.^13^^,^^14^	https://carbonmonitor.org/ https://doi.org/10.1038/s41597-020-00708-7
GCP-GridFED gridded fossil-fuel and cement CO_2_ emissions	Global Carbon Project; Jones et al.^59^	Version 2024.0 Zenodo: https://doi.org/10.5281/zenodo.13909046
Global gRidded dAily CO_2_ Emissions Dataset (GRACED)	Dou et al.^60^	https://carbonmonitor-graced.com/ https://doi.org/10.1038/s41597-023-01963-0
Global Fire Emissions Database version 4.1s (GFED4.1s)	van der Werf et al.^26^	https://www.globalfiredata.org/data.html https://doi.org/10.5194/essd-9-697-2017
Global Fire Assimilation System (GFAS) fire emissions	Copernicus Atmosphere Monitoring Service	https://atmosphere.copernicus.eu/global-fire-monitoring
ERA5 monthly averaged data on single levels	Copernicus Climate Change Service/ECMWF; Bell et al.^28^	https://doi.org/10.24381/cds.f17050d7
ERA5-Land monthly averaged data	Copernicus Climate Change Service/ECMWF	https://doi.org/10.24381/cds.68d2bb30
CRU JRA meteorological forcing data	University of East Anglia Climatic Research Unit/NERC EDS Center for Environmental Data Analysis	CRU JRA v2.5 https://catalogue.ceda.ac.uk/uuid/43ce517d74624a5ebf6eec5330cd18d5/
Multivariate ENSO Index version 2 (MEI.v2)	NOAA Physical Sciences Laboratory	https://psl.noaa.gov/enso/mei/
GRACE/GRACE-FO terrestrial water-storage data	Center for Space Research, The University of Texas at Austin	CSR RL06 Mascon Solutions https://doi.org/10.15781/cgq9-nh24 CSR data portal
IEA-EDGAR monthly CO_2_ emissions data used for monthly allocation of annual emissions	Crippa et al.^61^	https://doi.org/10.2760/4002897
CAMS OCO-2 atmospheric inversion flux output used in this study	This paper; Chevallier et al.^33^^,^^34^	Version FT24r2; available from the lead contact upon reasonable request
GONGGA OCO-2 atmospheric inversion flux output used in this study	This paper; Jin et al.^35^	Method/data description https://doi.org/10.5194/essd-16-2857-2024 Updated 2015–2024 output available from the lead contact upon reasonable request
CMS-Flux OCO-2 atmospheric inversion flux output used in this study	This paper; Liu et al.^36^	Method/data description https://doi.org/10.5194/essd-13-299-2021 Updated 2010–July 2024 output available from the lead contact upon reasonable request
SiB4/CarbonTracker Europe High-Resolution land-flux output used as an independent cross-check	This paper; van der Woude et al.^23^	Method/data description https://doi.org/10.5194/essd-15-579-2023 Output used in this paper available from the lead contact upon reasonable request
Processed data underlying the figures and tables and derived low-latency carbon-budget outputs	This paper	Available from the lead contact, Piyu Ke (kepiyu@gmail.com), upon reasonable request

**Software and algorithms**		

Custom data-processing, carbon-budget calculation, statistical-analysis, and visualization code	This paper	Available from the lead contact, Piyu Ke (kepiyu@gmail.com), upon reasonable request
Custom deep-learning emulator framework for the 19 DGVMs	This paper	Available from the lead contact, Piyu Ke (kepiyu@gmail.com), upon reasonable request
Custom near-real-time ocean CO_2_-flux emulator framework	Ke et al.^29^	arXiv: https://doi.org/10.48550/arXiv.2312.01637 Code available from the lead contact upon reasonable request
Growth Rates Using Satellite Observations (GRESO)	Pandey et al.^12^	https://doi.org/10.1029/2023AV001145
JULES dynamic global vegetation model	Wiltshire et al.^22^; Burton et al.^24^	https://doi.org/10.5194/gmd-14-2161-2021 https://doi.org/10.5194/gmd-12-179-2019
OCN dynamic global vegetation model	Zaehle and Friend^17^; Zaehle et al.^18^	https://doi.org/10.1029/2009GB003521 https://doi.org/10.1038/ngeo1207
ORCHIDEE-MICT dynamic global vegetation model	Krinner et al.,^19^ Vuichard et al.^20^	https://doi.org/10.1029/2003GB002199 https://doi.org/10.5194/gmd-12-4751-2019
LPJ-EOSIM dynamic global vegetation model	Fischer-Femal et al.^21^	Model implementation used in this paper; available from the model developers or lead contact upon reasonable request
SiB4/CarbonTracker Europe High-Resolution system	van der Woude et al.^27^	https://doi.org/10.5194/essd-15-579-2023
CAMS atmospheric CO_2_ inversion system	Chevallier et al.^33^^,^^34^	Version FT24r2 https://doi.org/10.5194/acp-19-14233-2019 https://doi.org/10.1029/2020GL090244
GONGGA atmospheric CO_2_ inversion system	Jin et al.^35^	https://doi.org/10.5194/essd-16-2857-2024
CMS-Flux atmospheric CO_2_ inversion system	Liu et al.^36^	https://doi.org/10.5194/essd-13-299-2021

### Method details

#### Atmospheric CO_2_ growth rate (CGR)

We used the monthly time series of globally averaged marine surface (MBL) atmospheric CO_2_ dry air mole fraction covering the period from January 1979 to November 2024, and the MLO station data from March 1958 to February 2025, both provided by NOAA’s Global Monitoring Laboratory (NOAA/GML).^5^^,^^6^ The July-July annual MBL growth rate is determined by averaging the most recent June and July months, corrected for the average seasonal cycle, and subtracting the average of the same period in the previous year (https://gml.noaa.gov/ccgg/trends/gl_gr.html). For the MLO data, the July-July annual mean growth rate is estimated using the average of the most recent May-August months, corrected for the average seasonal cycle, and subtracting the same four-month average of the previous year centered on July 1st (https://gml.noaa.gov/ccgg/trends/gr.html).

### Global fire CO_2_ emissions

Both the Global Fire Emissions Database version 4.1 including small fire burned area (GFED4.1s)^26^ and the Global Fire Assimilation System (GFAS, https://atmosphere.copernicus.eu/global-fire-monitoring) from Copernicus Atmosphere Monitoring Service (CAMS) are used to derive monthly global fire CO_2_ emissions. GFED4.1s combines satellite information on fire activity and vegetation productivity to estimate the gridded monthly burned area and fire emissions, and has a spatial resolution of 0.25 ° × 0.25 °.^26^ Note that GFED4.1s fire emissions for the 2017 and onwards period are from the beta version. The GFAS assimilates fire radiative power (FRP) observations from satellites to produce daily estimates of biomass burning emissions. We aggregated the GFAS daily fire emissions into monthly emissions.

#### Terrestrial CO_2_ fluxes

Terrestrial carbon fluxes are derived from the mean of four Dynamic Global Vegetation Models (DGVMs), specifically JULES, ORCHIDEE-MICT, OCN and LPJ-EOSIM. The methodology for estimating terrestrial carbon fluxes for the period 2010–2023 aligns with the TRENDY protocol, used in Global Carbon Budget,^62^ albeit with modifications due to the use of ERA5 climate forcing, and the fact that land-use forcing is not updated with such short latency. ERA5 forcing has been available since 1940,^28^ but preliminary simulations with DGVMs showed some issues with precipitation forcing before 1960. The simulations performed here correspond to the S2 experiment, starting from steady-state in 1960, with time varying climate and CO_2_ forcing, and land-use fixed at 2010, as described in Bastos et al.^63^

Since the model simulations start from a steady-state in the 1960s, they cannot account for the carbon balance before the industrial revolution, which leads to an underestimation of the mean trend compared to standard DGVMs. Therefore, we calibrated them using the 2019–2022 TRENDY DGVMs simulation 3 from the Global Carbon Budget 2023.^25^ This was done by calculating for each grid cell the median of the carbon flux from the TRENDY models during 2019–2022 and the mean of each of the DGVMs used in this study, then subtracting from each DGVM on each grid the difference so that they match the median flux of the TRENDY models. In other words, our DGVMs are used for predicting interannual anomalies of CO_2_ fluxes.

ERA5 meteorological forcing data can differ from CRU-JRA during extreme climatic conditions such as the 2023/24 El Niño event, especially in tropical precipitation.^64^ To evaluate whether the stronger tropical source anomalies in the ERA5-driven low-latency DGVMs could be partly associated with precipitation forcing, we calculated ERA5 and CRU-JRA precipitation anomalies for the same tropical regions and seasons used in the land-flux comparison. ERA5 total precipitation was converted to monthly totals following its monthly averaged daily accumulation convention, whereas CRU-JRA precipitation was summed from 6-hourly accumulated precipitation fields with units of mm per 6 h. Regional precipitation anomalies were calculated as area-weighted means relative to the 2015–2022 mean for the same calendar period, separately for JFM and AMJ 2024 (Table S4). This comparison is used as a forcing diagnostic rather than a full uncertainty propagation experiment.

#### Machine-learning emulators of DGVMs

To provide low-latency predictions of global land carbon fluxes for January–June 2024 (H1-2024), we also developed deep-learning-based emulators of 19 Dynamic Global Vegetation Models (DGVMs) participating in the Global Carbon Budget 2024. Each emulator was trained on monthly data from 2000 to 2023 at a 0.5 ° × 0.5 ° spatial resolution. Input features included atmospheric CO_2_ mole fraction, vegetation maps, lagged carbon fluxes from the previous 12 months and eight meteorological variables from the ERA5: surface pressure, surface downward short-wave radiation, surface downward long-wave radiation, 2-meter temperature, total precipitation, 10-meter wind u- and v-components, specific humidity. The emulators effectively capture the interannual variance in carbon flux anomalies at regional to continental scales (see Section S3 and Figure S3 for further details and performance evaluation). These emulators thus serve as both a robust method for nowcasting H1-2024 fluxes and as independent benchmarks against traditional DGVM estimates.

#### Ocean CO_2_ fluxes

Ocean carbon fluxes are derived from a suite of emulators based on both biogeochemical models and data-driven models. We updated estimates from 8 Global Ocean Biogeochemical Models and 8 data products included in the Global Carbon Budget 2022 to create a near-real-time framework.^29^ This update employs Convolutional Neural Networks (CNNs) and semi-supervised learning techniques to capture the non-linear relationships between model or product estimates and observed predictors. As a result, we obtain a near-real-time, monthly grid-based dataset of global surface ocean fugacity of CO_2_ and ocean-atmosphere CO_2_ flux data, extending to December 2023. Additional implementation and validation details are given in Ke et al.^29^ and in Section S2.

#### Anthropogenic CO_2_ emissions

For the period from 2010 to 2023, we used global fossil fuel and cement CO_2_ emissions estimates from the latest edition of the global carbon budget.^16^ For 2024, we used the average of emissions from the Carbon Monitor project based on near-real-time activity data from 6 sectors^13^^,^^14^^,^^15^ and from an updated estimate using the same methodology that the global Carbon Budget,^16^ i.e., based on energy data available by the time of the publication and projections for countries with no available data. The Carbon Monitor data give a global emission of +0.88% compared to 2023 while the Global Carbon Budget approach gives a global emission of +0.8% compared to 2023. We then applied monthly emissions proportions derived from the IEA-EDGAR monthly CO_2_ emissions dataset from Emissions Database for Global Atmospheric Research (EDGAR)^61^ to partition annual emissions into the first and second halves of each year. The same monthly distribution observed in 2023 was assumed for 2024 to estimate emissions specifically for H1-2024.

#### Atmospheric inversions

We used three atmospheric inversion driven by the OCO-2 satellite atmospheric CO_2_ column-average dry air mole fraction data, which all contributed to the annual CO_2_ budget assessment. CAMS has a net land CO_2_ flux over the period 2015–2022 being a sink of 1.93 GtC yr^−1^, CMS-Flux has a smaller mean land CO_2_ flux of 0.94 GtC yr^−1^ and GONGGA of 1.24 GtC yr^−1^. CAMS has a smaller mean net land CO_2_ flux being a sink in the northern extratropics (>30°N) of 1.51 GtC yr^−1^, CMS-Flux of 1.88 GtC yr^−1^, and GONGGA of 1.56 GtC yr^−1^. All inversions have very similar year-on-year anomalies of land CO_2_ fluxes since 2015.

#### CAMS inversion

This system provides global estimates of weekly greenhouse gas fluxes with a typical 4-month latency, now at a resolution of about 90 km based on a hexagonal-pentagonal mesh of the globe. The product used here is version FT24r2. It followed the usual production and quality control process of the CAMS products. It covers the OCO-2 period from October 2014 to September 2024, and its mean fluxes and anomalies are close to the median of inversions used in previous assessments^16^^,^^25^ (Figures S9C and S9D). The underlying transport model was nudged toward horizontal winds from the ERA5 reanalysis. The inferred fluxes were estimated in each horizontal grid point of the transport model with a temporal resolution of 8 days, separately for day-time and night-time. The prior values of the fluxes combine estimates of (i) gridded monthly fossil fuel and cement emissions (GCP-GridFED version 2024.0^59^) extended to year 2024 following Chevallier et al. (2020)^34^ using the emission changes reported by https://carbonmonitor.org/, together with anomalies in retrievals of NO_2_ columns from the Tropospheric Monitoring Instrument (TROPOMI, offline and processing and RPRO when available, van Geffen et al., 2019^65^), (ii) monthly ocean fluxes (Chau et al. 2024a,^31^ 2024b^66^), 3-hourly (when available) or monthly biomass burning emissions (GFAS) and climatological 3-hourly biosphere-atmosphere fluxes taken as the 1981–2020 mean of a simulation of the ORganizing Carbon and Hydrology In Dynamic EcosystEms model, version 2.2, revision 7262 (ORCHIDEE, Krinner et al. 2005^19^). The variational inversion accounts for spatial and temporal correlations of the prior errors, resulting in a total 1-sigma uncertainty for the prior fluxes over a full year of 3.0 GtC⋅yr^−1^ for the land pixels and of 0.2 GtC⋅yr^−1^ for the marine pixels.

### GONGGA inversion

This system provides global estimates of CO_2_ fluxes over the period of 2015–2024 at a spatial resolution of 2° by 2.5° (latitude×longitude) with 47 layers in the vertical direction from the surface to the top of the atmosphere. It follows the production and quality control process as described in Jin et al.^35^ The model is driven by Modern-Era Retrospective analysis for Research and Applications 2 (MERRA-2) meteorological data. The 3-h fluxes were optimized in each horizontal grid point of the transport model in each 14-day temporal window. The prior values of the fluxes combine estimates of (i) gridded monthly fossil fuel and cement emissions (GCP-GridFED version 2024.0^59^) extended to year 2024 using the Global gRidded dAily CO_2_ Emission Dataset-GRACED (https://carbonmonitor-graced.com/)^60^; (ii) gridded monthly ocean flux from CarbonTracker 2022 *p*CO_2_-Clim prior data, which were derived from the Takahashi et al. (2009) climatology of seawater *p*CO_2_; (iii) gridded monthly biosphere-atmosphere CO_2_ exchange fluxes simulated by ORCHIDEE-MICT model extended to year 2024 by repeating the fluxes in 2023; (iv) gridded monthly biomass burning emissions from the Global Fire Emissions Database (GFED; version 4.1s). The prior errors account for spatial and temporal correlations, resulting in a total 1-sigma uncertainty for the prior global fluxes over a full year of 4.7 GtC⋅yr^−1^ for land fluxes and of 0.3 GtC⋅yr^−1^ for ocean fluxes.

#### CMS-Flux inversion

This system provides global estimates of CO_2_ fluxes over the period of Jan 2010- July 2024 at a spatial resolution of 4° by 5° (latitude×longitude) with 47 layers in the vertical direction from the surface to the top of the atmosphere. The inversion setups follow Liu et al.^36^ The model is driven by Modern-Era Retrospective analysis for Research and Applications 2 (MERRA-2) meteorological data. CMS-Flux optimizes ocean and land fluxes at monthly temporal resolution, assuming known diurnal cycle from and the prior land and ocean carbon fluxes. The prior fluxes include (i) gridded monthly fossil fuel and cement emissions (GCP-GridFED version 2024.0^59^) extended to year 2024 using the Global gRidded dAily CO_2_ Emission Dataset-GRACED (https://carbonmonitor-graced.com/)^60^; (ii) 3-hourly land carbon fluxes including fire emissions from CARDAMOM; (iii) 3-hourly ocean fluxes from either ECCO-Darwin or daily ocean fluxes from MOM-6. We carried out two sets of top-down flux inversions with two different ocean prior fluxes, and the final results are the mean of the two flux inversions. CMS-Flux assumes no spatial and temporal correlations in the prior flux uncertainties. The 1-sigma uncertainties for land and ocean fluxes are 1.3 GtC⋅yr^−1^ and 0.3 GtC⋅yr^−1^ respectively.

#### Drought characteristics

The characteristics of meteorological drought in the tropics is described by the Cumulative Water Deficit (CWD). Based on or precipitation data from ERA5-Land, we calculated monthly CWD for each pixel, as:

(Equation 1) $${\text{CWD}}_{m}=\min\left(0,{\text{CWD}}_{m-1}{+P}_{m}-100\right)\;\text{if}\;P_{m}{\;<\;100,\text{else}\;\text{CWD}}_{m}=0$$

with *m* being the month within the hydrological year spanning from October of the previous year to September of the current year. CWD_m_ is the CWD of the current month, which is equal to the CWD from previous months (CWD_m-1_) plus the difference between the precipitation of the current month (P_m_) and ET (assumed to be 100 mm). The water deficit accumulates over the entire hydrological year. If monthly rainfall falls below 100 mm, the forest is considered to be experiencing a water deficit. For each grid cell, the *Z* score of CWD at the i^th^ grid for the m^th^ month is calculated as:

(Equation 2) $$Z_{CWD,m.i}=\frac{{CWD}_{m,i}-\mu_{CWD,m,i}}{\sigma_{CWD,m,i}}$$

where *μ*_*CWD*,*m*,*i*_ and *σ*_*CWD*,*m*,*i*_ are the mean value and standard deviation of CWD at the i^th^ grid for the m^th^ month during the reference period. We used the monthly Z_CWD_ to identify the spatial extent, duration, severity, onset, and end of the El Nino related drought events in the tropical forests. Drought intensity was classified into five categories, slight (Z ∈ [−1, 0)), mild (Z ∈ [−1.645, −1)), moderate (Z ∈ [−1.96, −1.645)), severe (Z ∈ [−2.576, −1.96)), and extreme (Z < −2.576).

### Quantification and statistical analysis

#### Definitions of trends, anomalies, and uncertainty metrics

Throughout this study, the July-to-July atmospheric CO2 growth-rate anomaly is defined differently from land and ocean flux anomalies because these quantities address different sources of variability. The July-to-July atmospheric CO2 growth-rate anomaly is computed as the residual from the long-term linear trend of the July-centered MBL growth-rate series shown in Figure 1A, with the trend estimated by ordinary least squares over the full July-to-July record available in this study (July 1979–July 2024). This detrending removes the secular rise in atmospheric CO2 growth rate associated with the long-term increase in atmospheric CO2 and isolates the interannual anomaly relevant to El Niño variability. By contrast, land and ocean CO2 flux anomalies are defined, unless otherwise stated, as departures from the 2015–2022 mean of the same calendar window (JFM, AMJ, H1, H2, or July-to-July). We use 2015–2022 as the reference period because it is the common pre-2023 interval available across the near-real-time top-down and bottom-up flux products used here, thereby allowing directly comparable flux anomalies across methods. The only exception is the inter-event comparison in Figure 5, for which anomalies are referenced to the 5-year pre-event mean of each El Niño episode, in order to place the 1997/98, 2015/16, and 2023/24 events on internally consistent event-specific baselines. This choice avoids imposing the shorter OCO-2 reference period on earlier events, but the resulting absolute magnitudes and rankings should be interpreted as conditional on the event-specific baseline definition.

Reported ±1σ values also have dataset-specific meanings. For DGVMs, inversions, emulators, GOBMs, and other multi-product estimates, values given as mean ±1σ denote the ensemble mean and the standard deviation across the contributing models or products; this quantity is used here as a measure of ensemble spread, rather than as a formal observational error or confidence interval. When we state that a flux anomaly is within 1σ of the 2015–2022 reference period, σ denotes the interannual standard deviation across the corresponding reference years for the same calendar window within the dataset considered. For atmospheric growth rates derived from a single time series, any accompanying ±1σ describes the variability of the monthly annual-growth-rate estimates entering the July-centered diagnostic and should not be interpreted as a formal measurement uncertainty. For fossil fuel and cement emissions, uncertainty is reported as the range across the two near-real-time estimates rather than as ±1σ.

## References

[1] Kim J.-S., Kug J.-S., Jeong S.-J. (2017). Intensification of terrestrial carbon cycle related to El Niño–Southern Oscillation under greenhouse warming. Nat. Commun..

[2] Liu J., Bowman K.W., Schimel D.S., Parazoo N.C., Jiang Z., Lee M., Bloom A.A., Wunch D., Frankenberg C., Sun Y. (2017). Contrasting carbon cycle responses of the tropical continents to the 2015–2016 El Niño. Science.

[3] Zhao M., Running S.W. (2010). Drought-Induced Reduction in Global Terrestrial Net Primary Production from 2000 Through 2009. Science.

[4] Humphrey V., Berg A., Ciais P., Gentine P., Jung M., Reichstein M., Seneviratne S.I., Frankenberg C. (2021). Soil moisture–atmosphere feedback dominates land carbon uptake variability. Nature.

[5] Lan X., Keeling R. Trends in Mauna Loa CO2 Determined from NOAA Global Monitoring Laboratory measurements. https://gml.noaa.gov/ccgg/trends/data.html.

[6] Lan X., Tans P., Thoning K.W. Trends in Globally-Averaged CO2 Determined from NOAA Global Monitoring Laboratory Measurements.

[7] NOAA Physical Sciences Laboratory (2025). Multivariate ENSO Index Version 2 (MEI.v2).

[8] Espinoza J.-C., Jimenez J.C., Marengo J.A., Schongart J., Ronchail J., Lavado-Casimiro W., Ribeiro J.V.M. (2024). The new record of drought and warmth in the Amazon in 2023 related to regional and global climatic features. Sci. Rep..

[9] Ke P., Ciais P., Sitch S., Li W., Bastos A., Liu Z., Xu Y., Gui X., Bian J., Goll D.S. (2024). Low latency carbon budget analysis reveals a large decline of the land carbon sink in 2023. Natl. Sci. Rev..

[10] Du X., Ciais P., Sitch S., Chevallier F., O’Sullivan M., Bastos A., Zaehle S., Ke P., Zhu L., Guo Z. (2025). Record-breaking high temperature amplifies the negative anomaly of tropical net land carbon sinks in the 2023-2024 El Niño. Agric. For. Meteorol..

[11] Wang H., Wang K., Wang T., Jin Z., Deng H., Gui Y., Chen W., Wang Y., Tian X., Wang T. (2026). AI-tracked halving of global land carbon sink in 2024. Sci. Bull..

[12] Pandey S., Miller J.B., Basu S., Liu J., Weir B., Byrne B., Chevallier F., Bowman K.W., Liu Z., Deng F. (2024). Toward Low-Latency Estimation of Atmospheric CO2 Growth Rates Using Satellite Observations: Evaluating Sampling Errors of Satellite and In Situ Observing Approaches. AGU Adv..

[13] Liu Z., Ciais P., Deng Z., Lei R., Davis S.J., Feng S., Zheng B., Cui D., Dou X., Zhu B. (2020). Near-real-time monitoring of global CO2 emissions reveals the effects of the COVID-19 pandemic. Nat. Commun..

[14] Liu Z., Ciais P., Deng Z., Davis S.J., Zheng B., Wang Y., Cui D., Zhu B., Dou X., Ke P. (2020). Carbon Monitor, a near-real-time daily dataset of global CO2 emission from fossil fuel and cement production. Sci. Data.

[15] Deng Z., Zhu B., Davis S.J., Ciais P., Guan D., Gong P., Liu Z. (2025). Global carbon emissions and decarbonization in 2024. Nat. Rev. Earth Environ..

[16] Friedlingstein P., O’Sullivan M., Jones M.W., Andrew R.M., Hauck J., Landschützer P., Le Quéré C., Li H., Luijkx I.T., Olsen A. (2025). Global Carbon Budget 2024. Earth Syst. Sci. Data.

[17] Zaehle S., Friend A.D. (2010). Carbon and nitrogen cycle dynamics in the O-CN land surface model: 1. Model description, site-scale evaluation, and sensitivity to parameter estimates. Glob. Biogeochem. Cycles.

[18] Zaehle S., Ciais P., Friend A.D., Prieur V. (2011). Carbon benefits of anthropogenic reactive nitrogen offset by nitrous oxide emissions. Nat. Geosci..

[19] Krinner G., Viovy N., de Noblet-Ducoudré N., Ogée J., Polcher J., Friedlingstein P., Ciais P., Sitch S., Prentice I.C. (2005). A dynamic global vegetation model for studies of the coupled atmosphere-biosphere system. Glob. Biogeochem. Cycles.

[20] Vuichard N., Messina P., Luyssaert S., Guenet B., Zaehle S., Ghattas J., Bastrikov V., Peylin P. (2019). Accounting for carbon and nitrogen interactions in the global terrestrial ecosystem model ORCHIDEE (trunk version, rev 4999): multi-scale evaluation of gross primary production. Geosci. Model Dev..

[21] Fischer-Femal B.J., Poulter B., Basu S., Miller J.B., Lehman S.J., Kaushik A., Colligan T., Michel S.E. (2025). Global Terrestrial C, 13C, and 14C Budgets from LPJ-iso, a New Isotope-Enabled Dynamic Global Vegetation Model. Authorea Prepr.

[22] Wiltshire A.J., Burke E.J., Chadburn S.E., Jones C.D., Cox P.M., Davies-Barnard T., Friedlingstein P., Harper A.B., Liddicoat S., Sitch S., Zaehle S. (2021). JULES-CN: a coupled terrestrial carbon–nitrogen scheme (JULES vn5.1). Geosci. Model Dev..

[23] Sellar A.A., Jones C.G., Mulcahy J.P., Tang Y., Yool A., Wiltshire A., O’Connor F.M., Stringer M., Hill R., Palmieri J. (2019). UKESM1: Description and Evaluation of the U.K. Earth System Model. J. Adv. Model. Earth Syst..

[24] Burton C., Betts R., Cardoso M., Feldpausch T.R., Harper A., Jones C.D., Kelley D.I., Robertson E., Wiltshire A. (2019). Representation of fire, land-use change and vegetation dynamics in the Joint UK Land Environment Simulator vn4.9 (JULES). Geosci. Model Dev..

[25] Friedlingstein P., O’Sullivan M., Jones M.W., Andrew R.M., Bakker D.C.E., Hauck J., Landschützer P., Le Quéré C., Luijkx I.T., Peters G.P. (2023). Global Carbon Budget 2023. Earth Syst. Sci. Data.

[26] van der Werf G.R., Randerson J.T., Giglio L., van Leeuwen T.T., Chen Y., Rogers B.M., Mu M., van Marle M.J.E., Morton D.C., Collatz G.J. (2017). Global fire emissions estimates during 1997–2016. Earth Syst. Sci. Data.

[27] van der Woude A.M., de Kok R., Smith N., Luijkx I.T., Botía S., Karstens U., Kooijmans L.M.J., Koren G., Meijer H.A.J., Steeneveld G.-J. (2023). Near-real-time CO_2_ fluxes from CarbonTracker Europe for high-resolution atmospheric modeling. Earth Syst. Sci. Data.

[28] Bell B., Hersbach H., Simmons A., Berrisford P., Dahlgren P., Horányi A., Muñoz-Sabater J., Nicolas J., Radu R., Schepers D. (2021). The ERA5 global reanalysis: Preliminary extension to 1950. Q. J. R. Meteorol. Soc..

[29] Ke P., Gui X., Cao W., Wang D., Hou C., Wang L., Song X., Li Y., Zhu B., Bian J. (2023). Near-real-time monitoring of global ocean carbon sink. arXiv.

[30] Friedlingstein P., O’Sullivan M., Jones M.W., Andrew R.M., Gregor L., Hauck J., Le Quéré C., Luijkx I.T., Olsen A., Peters G.P. (2022). Global Carbon Budget 2022. Earth Syst. Sci. Data.

[31] Chau T.-T.-T., Chevallier F., Gehlen M. (2024). Global Analysis of Surface Ocean CO2 Fugacity and Air-Sea Fluxes With Low Latency. Geophys. Res. Lett..

[32] O’Dell C.W., Eldering A., Wennberg P.O., Crisp D., Gunson M.R., Fisher B., Frankenberg C., Kiel M., Lindqvist H., Mandrake L. (2018). Improved retrievals of carbon dioxide from Orbiting Carbon Observatory-2 with the version 8 ACOS algorithm. Atmos. Meas. Tech..

[33] Chevallier F., Remaud M., O’Dell C.W., Baker D., Peylin P., Cozic A. (2019). Objective evaluation of surface- and satellite-driven carbon dioxide atmospheric inversions. Atmos. Chem. Phys..

[34] Chevallier F., Zheng B., Broquet G., Ciais P., Liu Z., Davis S.J., Deng Z., Wang Y., Bréon F.-M., O’Dell C.W. (2020). Local Anomalies in the Column-Averaged Dry Air Mole Fractions of Carbon Dioxide Across the Globe During the First Months of the Coronavirus Recession. Geophys. Res. Lett..

[35] Jin Z., Tian X., Wang Y., Zhang H., Zhao M., Wang T., Ding J., Piao S. (2024). A global surface CO_2_ flux dataset (2015–2022) inferred from OCO-2 retrievals using the GONGGA inversion system. Earth Syst. Sci. Data.

[36] Liu J., Baskaran L., Bowman K., Schimel D., Bloom A.A., Parazoo N.C., Oda T., Carroll D., Menemenlis D., Joiner J. (2021). Carbon Monitoring System Flux Net Biosphere Exchange 2020 (CMS-Flux NBE 2020). Earth Syst. Sci. Data.

[37] Chatterjee A., Gierach M.M., Sutton A.J., Feely R.A., Crisp D., Eldering A., Gunson M.R., O’Dell C.W., Stephens B.B., Schimel D.S. (2017). Influence of El Niño on atmospheric CO2 over the tropical Pacific Ocean: Findings from NASA’s OCO-2 mission. Science.

[38] Payne V., Chatterjee A., Kiel M., O’Del C., Fisher B., Das S., Kuai L., Osterman G. (2024).

[39] Regnier P., Resplandy L., Najjar R.G., Ciais P. (2022). The land-to-ocean loops of the global carbon cycle. Nature.

[40] Copernicus Climate Change Service (2025). Copernicus: 2024 is the First Year to Exceed 1.5°C above Pre-industrial Level.

[41] Copernicus Climate Change Service (2025). Global Climate Highlights 2024.

[42] Jha R., Perkins-Kirkpatrick S.E., Singh D., Kimutai J., Libonati R., Mondal A. (2025). Extreme terrestrial heat in 2024. Nat. Rev. Earth Environ..

[43] Wang K., Wang Y., Wang X., He Y., Li X., Keeling R.F., Ciais P., Heimann M., Peng S., Chevallier F. (2020). Causes of slowing-down seasonal CO2 amplitude at Mauna Loa. Glob. Chang. Biol..

[44] Zhang Y., Li W., Sun G., Mao J., Dannenberg M., Xiao J., Li Z., Zhao H., Zhang Q., Hu S. (2025). Contrasting biological production trends over land and ocean. Nat. Clim. Chang..

[45] Müller J.D., Gruber N., Schneuwly A., Bakker D.C.E., Gehlen M., Gregor L., Hauck J., Landschützer P., McKinley G.A. (2025). Unexpected decline in the ocean carbon sink under record-high sea surface temperatures in 2023. Nat. Clim. Chang..

[46] Yang H., Ciais P., Chave J., Huang Y., Ballantyne A.P., Yu K., Berzaghi F., Wigneron J.-P. (2021). Coarse woody debris are buffering mortality-induced carbon losses to the atmosphere in tropical forests. Environ. Res. Lett..

[47] Jury M.R., Enfield D.B., Mélice J.-L. (2002). Tropical monsoons around Africa: Stability of El Niño–Southern Oscillation associations and links with continental climate. J. Geophys. Res..

[48] Marengo J.A., Cunha A.P., Espinoza J.-C., Fu R., Schöngart J., Jimenez J.C., Costa M.C., Ribeiro J.M., Wongchuig S., Zhao S. (2024). The drought of Amazonia in 2023-2024. Am. J. Clim. Change.

[49] Moreira D.M., Papa F., Fassoni-Andrade A., Fleischmann A.S., Wongchuig S., Paiva R., Paris A., Frappart F., Melo J.S., Crétaux J.-F. (2025). Widespread and exceptional reduction in river water levels across the Amazon Basin during the 2023 extreme drought revealed by satellite altimetry and SWOT. Geophys. Res. Lett..

[50] Souza C.M., Marengo J., Ferreira B., Ribeiro J., Schirmbeck L.W., Schirmbeck J., Hirye M., Cunha A., Wiederhecker H.C., Latuf M.O. (2024). Amazon severe drought in 2023 triggered surface water loss. Environ. Res. Clim..

[51] Kolden C.A., Abatzoglou J.T., Jones M.W., Jain P. (2025). Wildfires in 2024. Nat. Rev. Earth Environ..

[52] Jung M., Reichstein M., Schwalm C.R., Huntingford C., Sitch S., Ahlström A., Arneth A., Camps-Valls G., Ciais P., Friedlingstein P. (2017). Compensatory water effects link yearly global land CO2 sink changes to temperature. Nature.

[53] Brienen R.J.W., Phillips O.L., Feldpausch T.R., Gloor E., Baker T.R., Lloyd J., Lopez-Gonzalez G., Monteagudo-Mendoza A., Malhi Y., Lewis S.L. (2015). Long-term decline of the Amazon carbon sink. Nature.

[54] Phillips O.L., van der Heijden G., Lewis S.L., López-González G., Aragão L.E.O.C., Lloyd J., Malhi Y., Monteagudo A., Almeida S., Dávila E.A. (2010). Drought–mortality relationships for tropical forests. New Phytol..

[55] Bennett A.C., Rodrigues de Sousa T., Monteagudo-Mendoza A., Esquivel-Muelbert A., Morandi P.S., Coelho de Souza F., Castro W., Duque L.F., Flores Llampazo G., Manoel dos Santos R. (2023). Sensitivity of South American tropical forests to an extreme climate anomaly. Nat. Clim. Chang..

[56] Harmon M.E., Fasth B.G., Yatskov M., Kastendick D., Rock J., Woodall C.W. (2020). Release of coarse woody detritus-related carbon: a synthesis across forest biomes. Carbon Balance Manag..

[57] Dalagnol R., Wagner F.H., Galvão L.S., Braga D., Osborn F., Sagang L.B., da Conceição Bispo P., Payne M., Silva Junior C., Favrichon S. (2023). Mapping tropical forest degradation with deep learning and Planet NICFI data. Remote Sens. Environ..

[58] Chen Y., Hall J., van Wees D., Andela N., Hantson S., Giglio L., van der Werf G.R., Morton D.C., Randerson J.T. (2023). Multi-decadal trends and variability in burned area from the fifth version of the Global Fire Emissions Database (GFED5). Earth Syst. Sci. Data.

[59] Jones M.W., Andrew R.M., Peters G.P., Janssens-Maenhout G., De-Gol A.J., Ciais P., Patra P.K., Chevallier F., Le Quéré C. (2021). Gridded fossil CO2 emissions and related O2 combustion consistent with national inventories 1959–2018. Sci. Data.

[60] Dou X., Hong J., Ciais P., Chevallier F., Yan F., Yu Y., Hu Y., Huo D., Sun Y., Wang Y. (2023). Near-real-time global gridded daily CO2 emissions 2021. Sci. Data.

[61] Crippa M., Guizzardi D., Pagani F., Banja M., Muntean M., Schaaf E., Becker W., Monforti-Ferrario F., Quadrelli R., Risquez Martin A. (2024).

[62] Sitch S., O’Sullivan M., Robertson E., Friedlingstein P., Albergel C., Anthoni P., Arneth A., Arora V.K., Bastos A., Bastrikov V. (2024). Trends and Drivers of Terrestrial Sources and Sinks of Carbon Dioxide: An Overview of the TRENDY Project. Glob. Biogeochem. Cycles.

[63] Bastos A., Ciais P., Friedlingstein P., Sitch S., Pongratz J., Fan L., Wigneron J.P., Weber U., Reichstein M., Fu Z. (2020). Direct and seasonal legacy effects of the 2018 heat wave and drought on European ecosystem productivity. Sci. Adv..

[64] Lavers D.A., Simmons A., Vamborg F., Rodwell M.J. (2022). An evaluation of ERA5 precipitation for climate monitoring. Q. J. R. Meteorol. Soc..

[65] van Geffen J.H.G.M., Eskes H.J., Boersma K.F., Maasakkers J.D., Veefkind J.P. (2019). TROPOMI ATBD of the Total and Tropospheric NO2 Data Products (KNMI).

[66] Chau T.-T.-T., Gehlen M., Metzl N., Chevallier F. (2024). CMEMS-LSCE: a global, 0.25∖degree, monthly reconstruction of the surface ocean carbonate system. Earth Syst. Sci. Data.

