# Supplementary material for: Low latency global carbon budget reveals land sink decline during 2023 to 2024 El Niño
Source: iScience. 2026 Aug 10;29(8):117053. doi: 10.1016/j.isci.2026.117053 (PMC13486359; doi:10.1016/j.isci.2026.117053)
Supplement: Supplementary file 1 [file mmc1.pdf]

iScience, Volume *29*

Supplemental information

Low latency global carbon budget reveals land sink decline during 2023 to 2024 El Niño

Piyu Ke, Philippe Ciais, Yitong Yao, Stephen Sitch, Wei Li, Yidi Xu, Xiaomeng Du, Xiaofan Gui, Ana Bastos, Sönke Zaehle, Ben Poulter, Thomas Colligan, Auke M. van der Woude, Wouter Peters, Zhu Liu, Zhe Jin, Xiangjun Tian, Yilong Wang, Junjie Liu, Sudhanshu Pandey, Chris O'Dell, Jiang Bian, Chuanlong Zhou, John B. Miller, Xin Lan, Jefferson Goncalves De Souza, Michael O'Sullivan, Pierre Friedlingstein, Guido R. van der Werf, Glen P. Peters, Shilong Piao, and Frédéric Chevallier

Supplemental methods

1. Global July-July annual growth rates of atmospheric CO_2 from different atmospheric observations and bottom-up models

We compared the atmospheric CO_2 growth rate from July 2010 to July 2024 based on in-situ observations from 40 marine boundary layer background stations, calculated by NOAA GML[5,6] as the year on year difference between smoothed observations between May-August averaged across all the stations (blue bars), from the Mauna Loa station (dark blue squares), from the assimilation of global OCO-2 satellite observations of column-average CO_2 dry air mole fraction retrievals, about 300,000 10-second-averaged retrievals each year, by the inversions used in this study (brown curve), OCO-2 satellite data processed with the data-driven Growth Rate From Satellite Observations (GRESO) approach of ref. [12] (orange squares), and by the bottom-up approach, that is, not using atmospheric measurements and the growth rate is predicted from the difference between fossil CO_2 emissions minus the land sink from four DGVM models, minus the ocean sink from ocean model emulators (red curve) (Supplementary Fig. 1c).

The Growth Rate From Satellite Observations (GRESO) method processes OCO-2 satellite soundings to derive CO_2 growth rates. It involves two main stages: (a) sequential aggregation and (b) time series processing. First, individual soundings are aggregated into weighted 10-second averages, filtered for quality and outliers, and then gridded (5°x5°, 16-day). These gridded data are averaged spatially (first by longitude, then using latitude-weighting) to create a representative global CO_2 time series. Second, the CO_2 time series is interpolated, deseasonalized, and sampled at the edge of months. Then, consecutive month-edge values are different to calculate deseasonalized monthly and, subsequently, annual CO_2 growth rates. Additional 5-month smoothing is applied on monthly growth rates to reduce noise. Here we used only the LNLG (Land Nadir Land Glint) OCO-2 XCO_2 data.

2. Maps of monthly air-sea CO_2 fluxes from emulators of biogeochemical and data driven ocean models

We utilized a data-driven deep learning technique for near-real-time estimation of oceanic carbon monthly gridded fluxes [29]. This method integrates year, month, latitude, longitude, and nine environmental factors as predictors, targeting predictions for each GOBM model or ocean data product. We utilize monthly data up to the end of 2021 from 5 GOBMs and 8 data products in the Global Carbon Budget 2022, with fCO_2 output provided at a 1° × 1°

resolution. Our predictive variables include a range of biological, chemical, and physical factors typically linked to fluctuations in fCO_2. These variables are sea surface temperature (SST), sea ice fraction (ICE), sea surface salinity (SSS), atmospheric CO_2 mole fraction (xCO_2), mixed-layer depth (MLD), sea surface height (SSH), chlorophyll a (chl a), sea level pressure (SLP), and wind speed. All data are bilinearly interpolated to a 1° × 1° monthly resolution to align with our fCO_2 targets and updated to June 2024. Since the xCO_2 data is only available up to the end of 2023, and to meet the requirement for near-real-time data, we gather global average marine boundary layer surface monthly mean atmospheric CO_2 data updated to June 2024. We use a Linear Regression model (LR) model to establish a relationship among the year, month, latitude, longitude, mean atmospheric CO_2 data, and xCO_2. Additionally, we incorporated valuable features: xCO_2 data from the same month over the past six years. We used data from 2015-2022, and testing on 2023 data yielded a test RMSE of 0.40, reflecting roughly a 0.1% prediction error. This approach allows us to extend the xCO_2 data to near-real-time. All data are formatted into a 180x360 grid and subdivided into 18x18 patches for computational efficiency. During the training process, we incorporated predictors from the current month, as well as predictors and historical fCO_2 data from the past 12 months, as auxiliary information. Consequently, the input data is ultimately processed into a size of 18x18x14. Our experiments demonstrated that including auxiliary information from the previous 12 months significantly enhances the model's accuracy. This improvement is likely due to the lagged effects of some predictors and the corrective influence of historical fCO_2 data on the model's predictions.

After processing the data, we employed a Semi-supervised learning framework combined with Convolutional Neural Network (CNN) models for prediction. Our architecture integrates multi-layered CNNs and linear models to efficiently process the input data. The CNNs, inspired by human visual perception, consist of Convolutional Layers, Rectified Linear Unit (ReLU) Layers, and Fully Connected Layers, which collectively transform input data into predictions. The input layer has dimensions of 18x18x14, followed by two hidden layers of 18x18x64 that learn spatial hierarchies. Linear layers, also 18x18x64, perform linear transformations. The output layer, with dimensions of 18x18x1, outputs the predicted oceanic carbon fCO_2 value. To enhance stability in areas with missing values and at the land-sea interface, we used a semi-supervised model framework. Initially, the CNN was trained on labeled data, calculating the Root Mean Square Error (RMSE) between labels and predictions as the supervised loss (LsL_sLs). For prediction stability on unlabeled data, we used pseudo-labeling: predicting with 10% of input predictors removed as pseudo-labels, then predicting again with 30% of input predictors removed, calculating the RMSE as the unsupervised loss (LuL_uLu). The model was updated using the weighted sum of LsL_sLs

and LuL_uLu through backward propagation. This architecture enables efficient and accurate prediction of oceanic carbon flux.

3. Maps of monthly terrestrial CO_2 fluxes from emulators of Dynamic Global Vegetation Models

We applied a deep-learning emulator framework identical to that used for oceanic fluxes to estimate monthly terrestrial carbon fluxes. Specifically, we utilized monthly terrestrial CO_2 flux data from simulation 2 of 19 Dynamic Global Vegetation Models (DGVMs) included in the Global Carbon Budget 2024 [16], covering the period up to December 2023 at a standardized spatial resolution of 0.5° × 0.5°. Predictor variables comprised atmospheric CO_2 mole fraction and eight meteorological variables from the ERA5 [28]: surface pressure, surface downward short-wave radiation, surface downward long-wave radiation, 2-meter temperature, total precipitation, 10-meter wind components, and specific humidity. All predictors were bilinearly interpolated to the uniform monthly spatial resolution. The model was trained and validated using data spanning the period from 2000 to 2023, and subsequently employed to predict H1-2024. Additionally, we conducted performance testing on the emulators. Specifically, data from 2022 to 2023 was withheld from the training and validation datasets (2000–2021) to serve as an independent test set for evaluating model performance. Supplementary Fig. 3 provides a detailed comparison between emulator predictions and their corresponding DGVM outputs (their low latency versions were used in this study). The results demonstrate excellent agreement, highlighting the robustness and accuracy of the emulator framework.

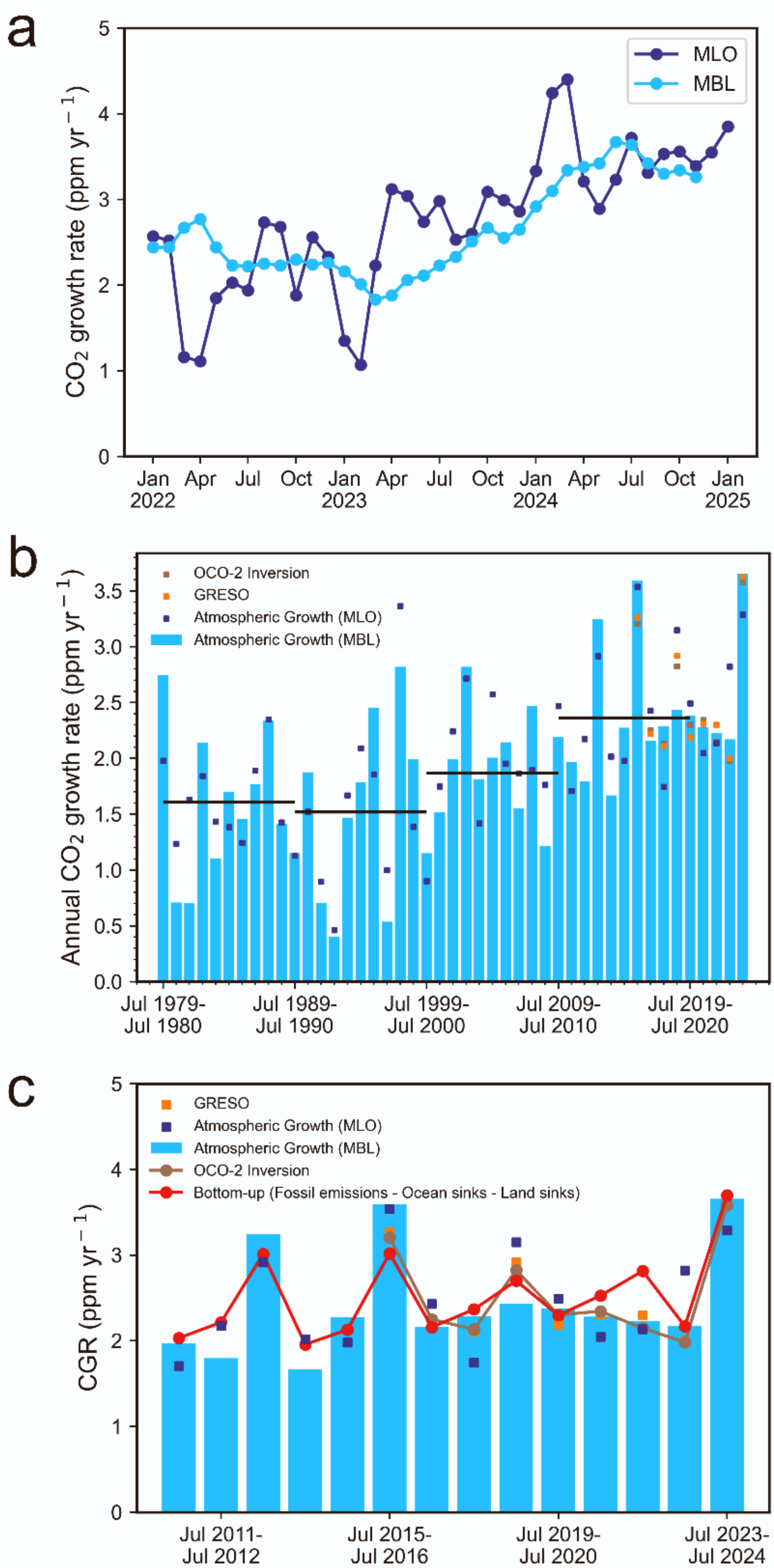

Supplementary Figure 1 (a) Monthly running mean of annual CO_2 growth rates with marine boundary layer surface stations in blue (MBL) and Mauna Loa (MLO) in dark blue (MLO) from January 2022 to January 2025. **(b)** Annual June-July to June-July growth rates from

MBL stations (blue bars), MLO (dark blue), OCO-2 column CO_2 data assimilated by inversions (brown squares), and OCO-2 satellite data processed with the data-driven GRESO approach of ref. [12] (orange squares). **(c)** June-July to June-July CO_2 growth rates from July 2010 to July 2024 from different approaches, including MBL stations (blue bars), MLO (dark blue squares), the assimilation of global OCO-2 column CO_2 concentration (brown curve), GRESO (orange squares), and the bottom-up approach where the growth rate is calculated from the difference between fossil fuel CO_2 emissions minus the sum of the net land CO_2 flux from four DGVM models and the ocean sink from ocean model emulators (red curve).

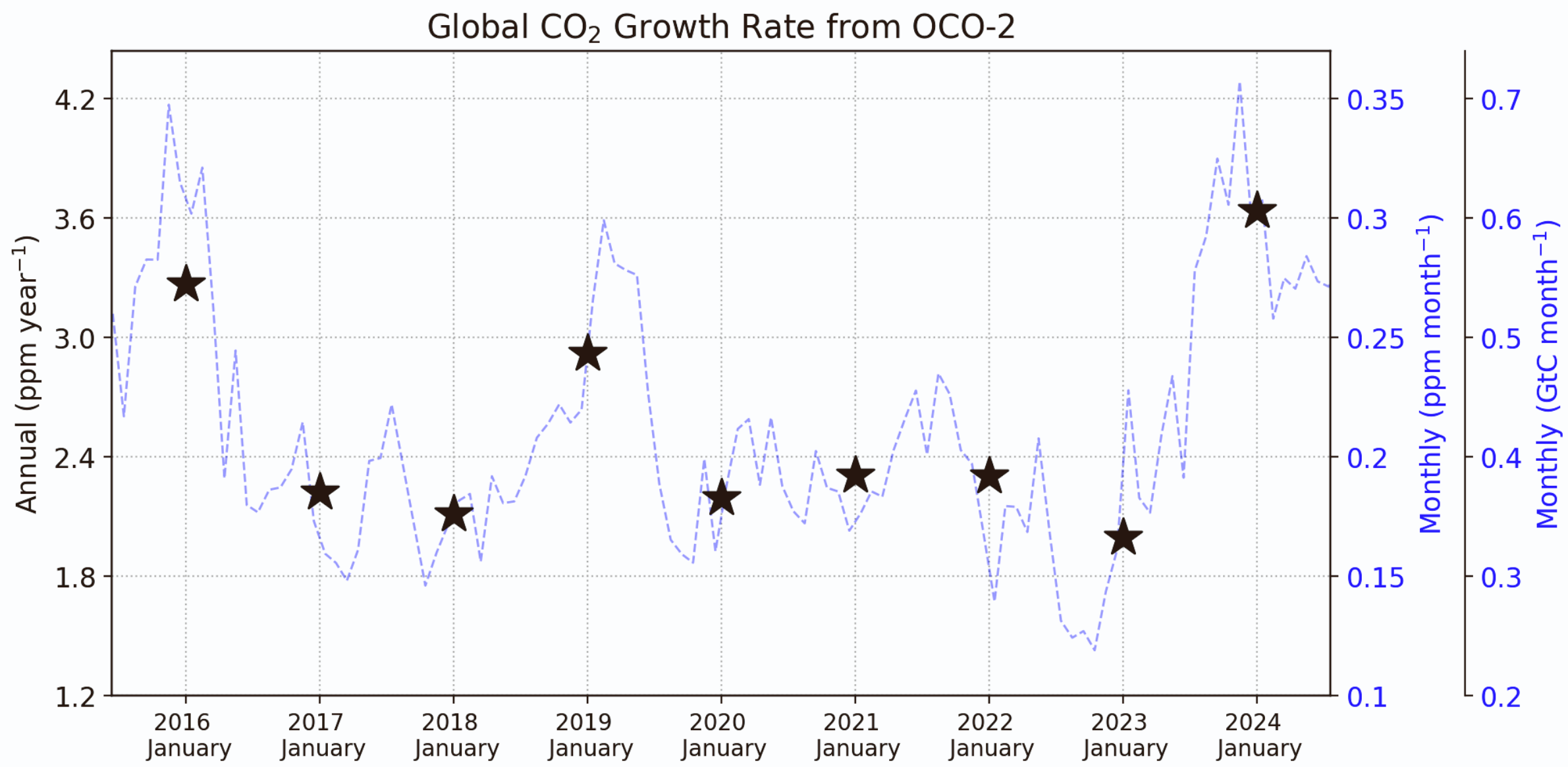

Supplementary Figure 2 Annual and monthly global CO_2 growth rates from OCO-2 data using the data-driven GRESO approach. Black stars indicate the annual growth rate measured from June–July to June–July of consecutive years (left y-axis, in ppm $year^{-1}$), while the blue dashed line represents the monthly growth rate (from the start to the end of each month; right y-axis, in ppm/month). A five-month running mean is applied to the monthly time series to reduce noise. Growth rate values were calculated using the Growth Rates Using Satellite Observations (GRESO) approach from ref. [12].

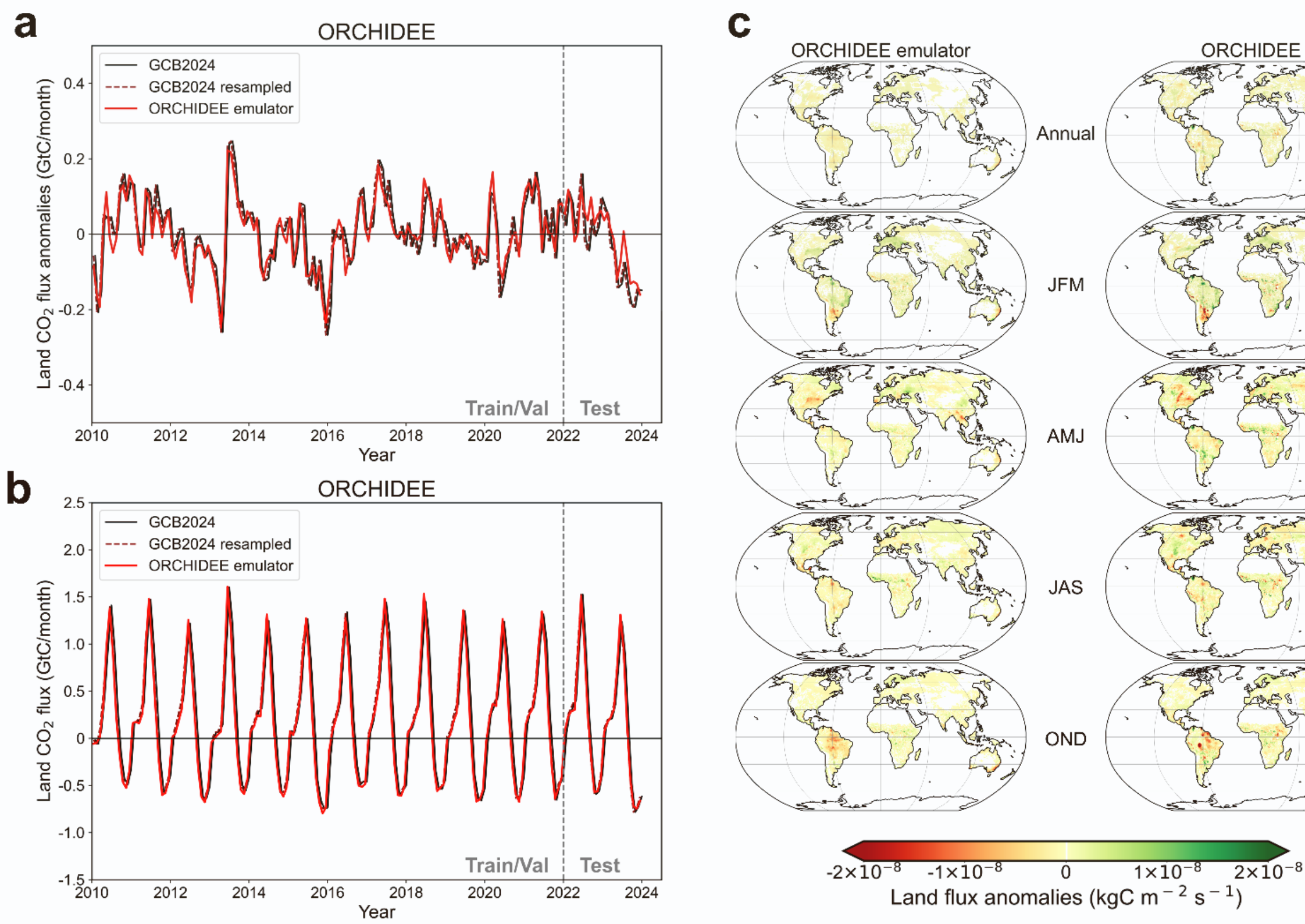

a
ORCHIDEE
GCB2024
GCB2024 resampled
ORCHIDEE emulator
Land CO_2 flux anomalies (GtC/month)
Train/Val
Test
Year
b
ORCHIDEE
Land CO_2 flux (GtC/month)
c
ORCHIDEE emulator
ORCHIDEE
Annual
JFM
AMJ
JAS
OND
Land flux anomalies (kgC m^{-2} s^{-1})

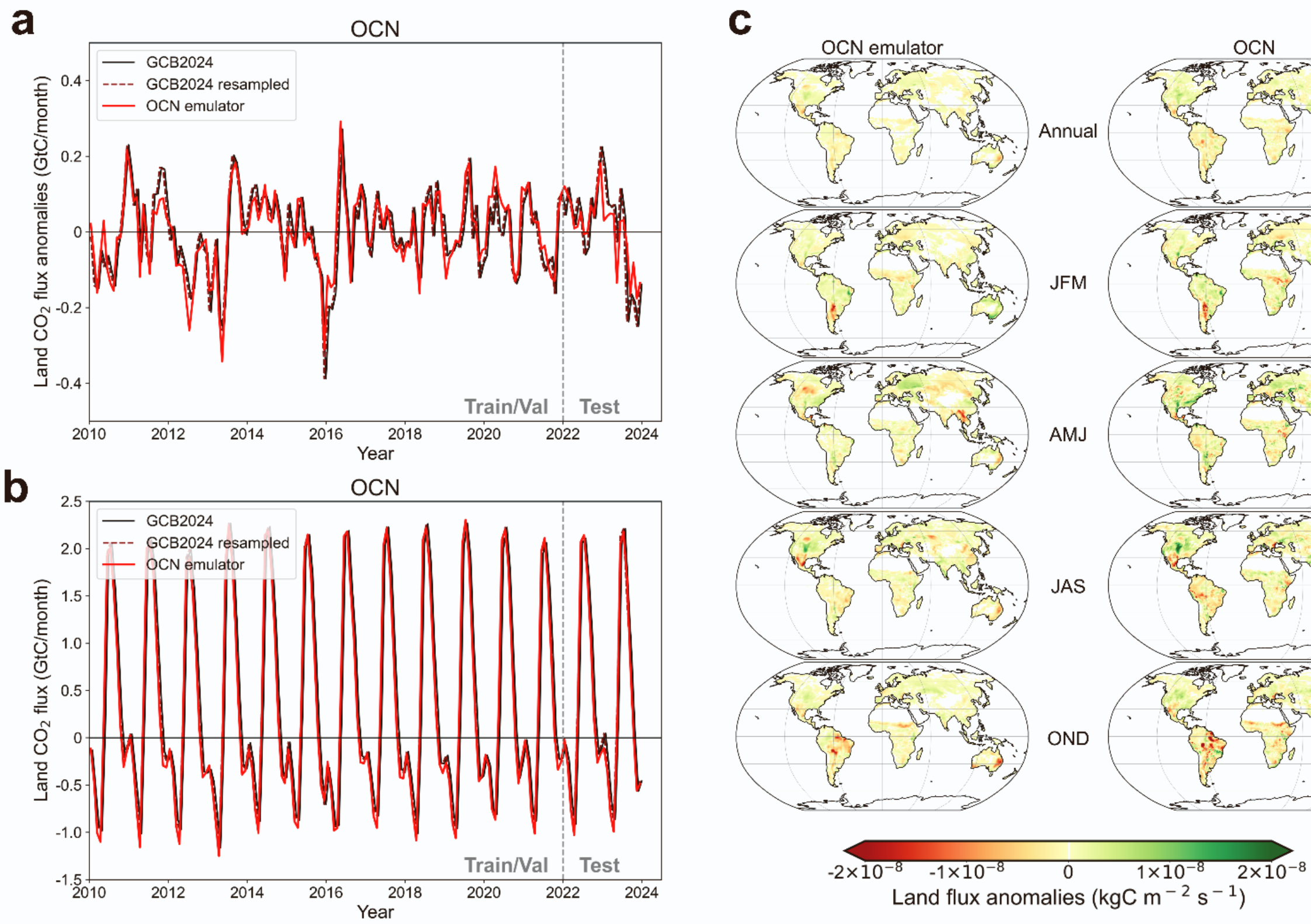

a
OCN
GCB2024
GCB2024 resampled
OCN emulator
Land CO_2 flux anomalies (GtC/month)
Train/Val
Test
Year
b
OCN
Land CO_2 flux (GtC/month)
c
OCN emulator
OCN
Annual
JFM
AMJ
JAS
OND
Land flux anomalies (kgC m^{-2} s^{-1})

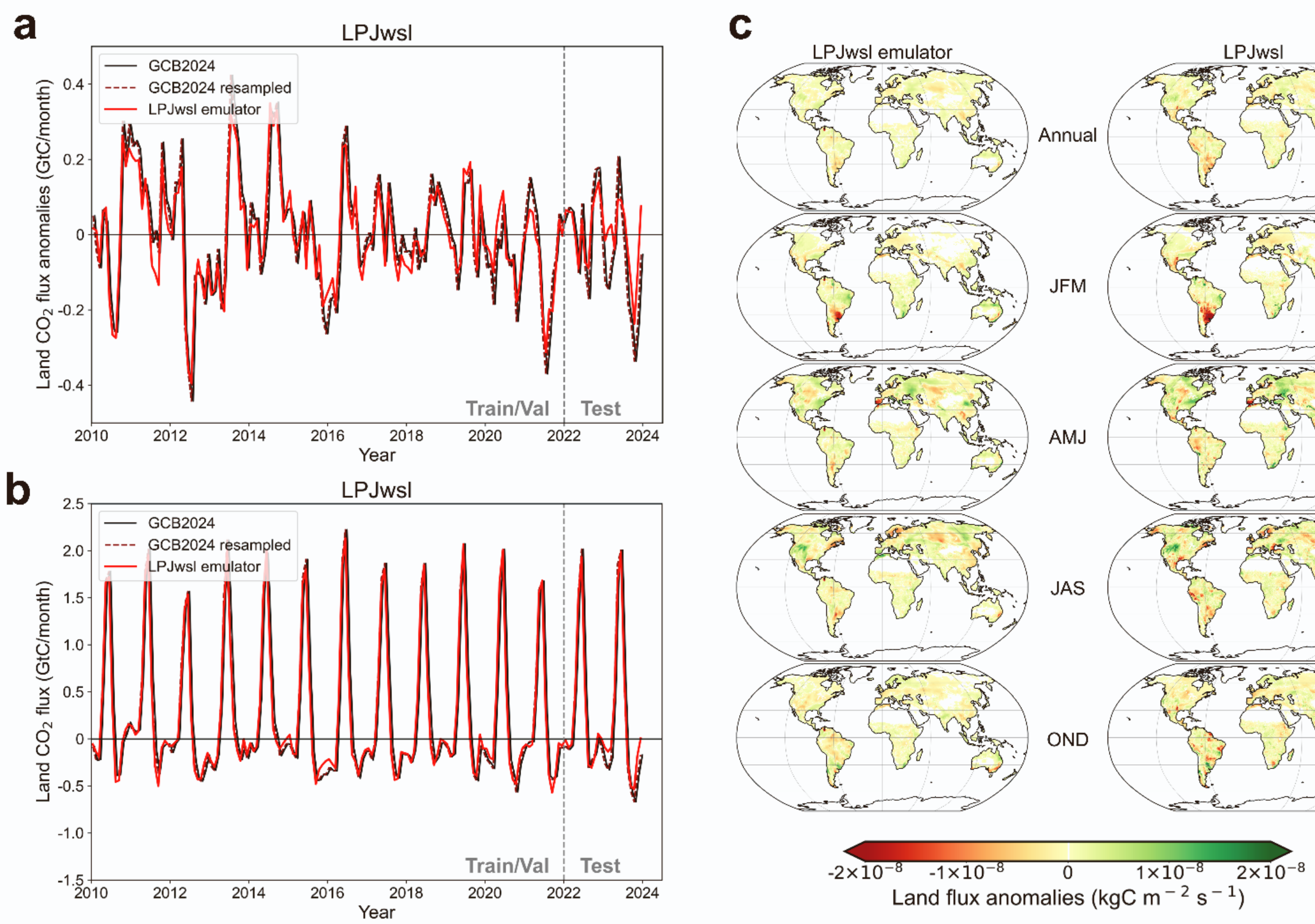

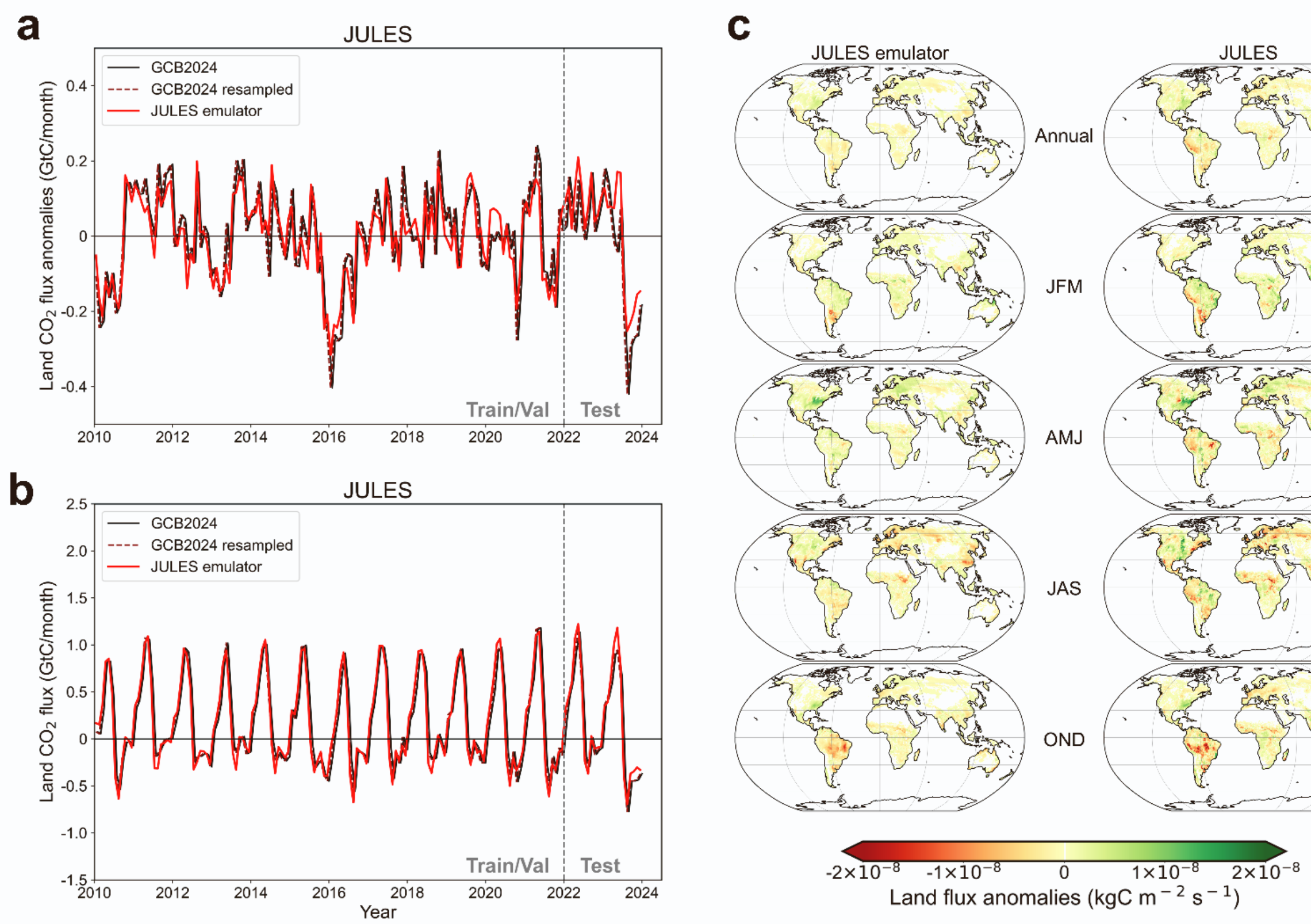

Supplementary Figure 3 Comparison of DGVM emulators with their original models for ORCHIDEE, OCN, LPJ-wsl, and JULES. The emulators were trained and validated using data from 2000–2021, and tested in 2022 and 2023. **(a)** Time series of deseasonalized monthly anomalies from 2010-2023 with original models in black and emulator results in red. The red dashed line represents the original model outputs resampled to 0.5° resolution, which served as the training target for all emulators (top left). **(b)** Time series of net land CO_2

fluxes from Jan. 1st 2010 to Dec. 31st 2023 (bottom left), and **(c)** spatial distribution of net land CO_2 fluxes anomalies for each quarter in 2023 with emulator on the left and native model on the right.

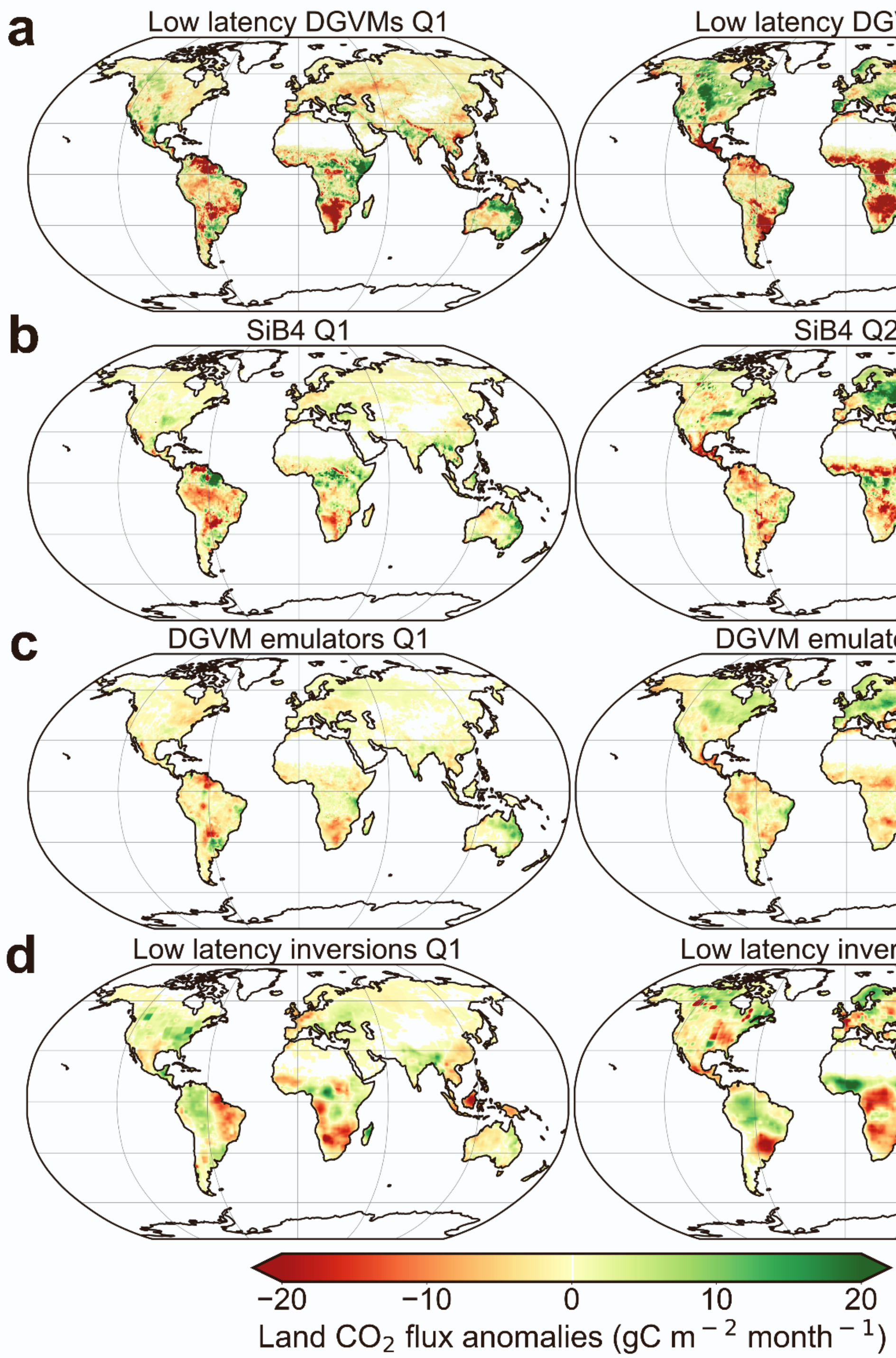

Supplementary Figure 4 Land CO_2 flux anomalies for the first and second quarters in 2024 (Q1 = JFM and Q2 = AMJ). Anomalies are calculated from the 2015-2022 average of each quarter. **(a)** mean of the four low latency DGVMs used in this study, **(b)** SiB4 flux model, **(c)** mean of the 19 DGVM emulators and **(d)** mean of the three OCO-2 inversions. Positive values in green represent increased flux from the atmosphere to the land, that is anomalous carbon sinks.

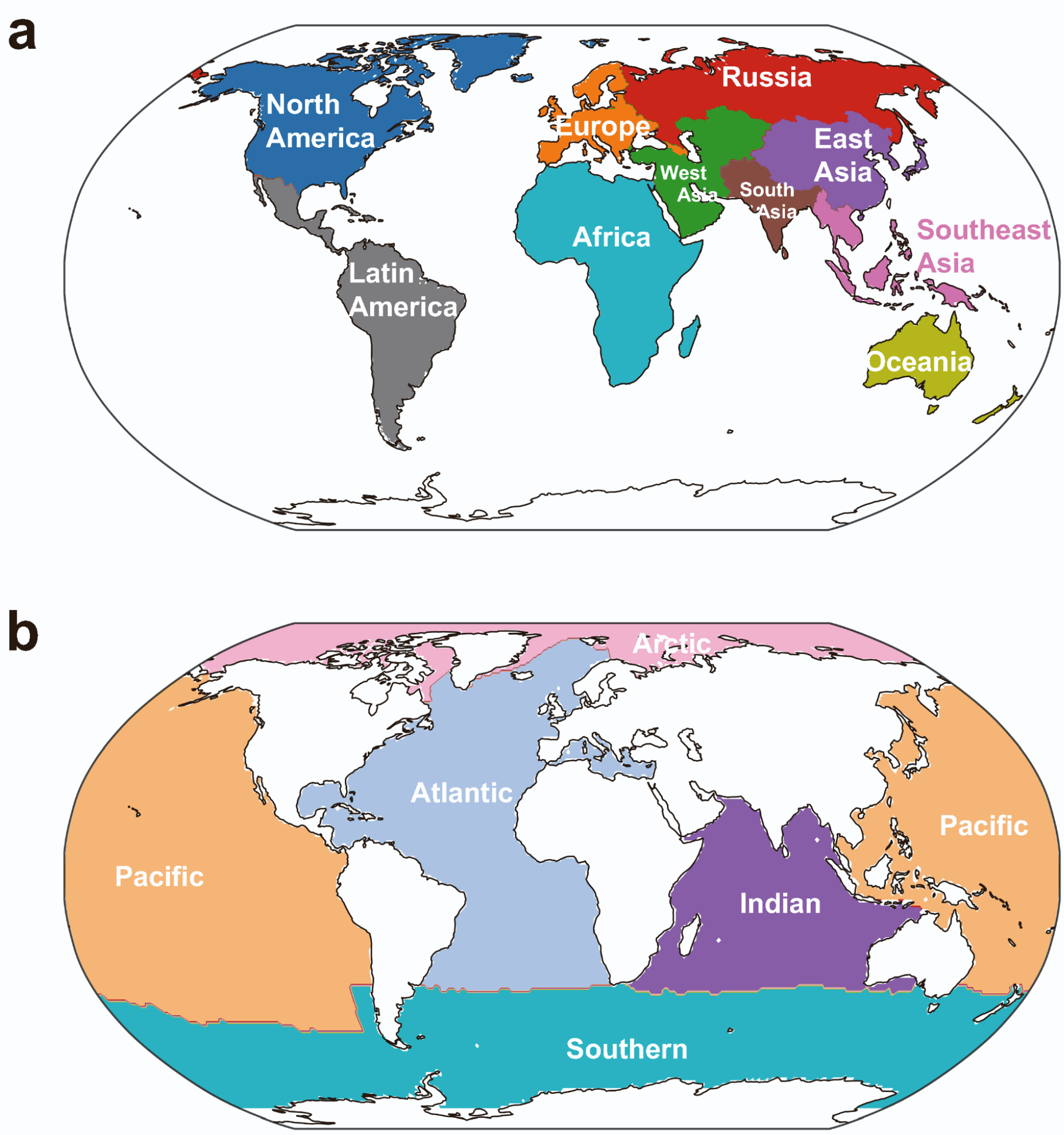

Supplementary Figure 5 Maps of RECCAP2 land (a) and ocean (b) regions.

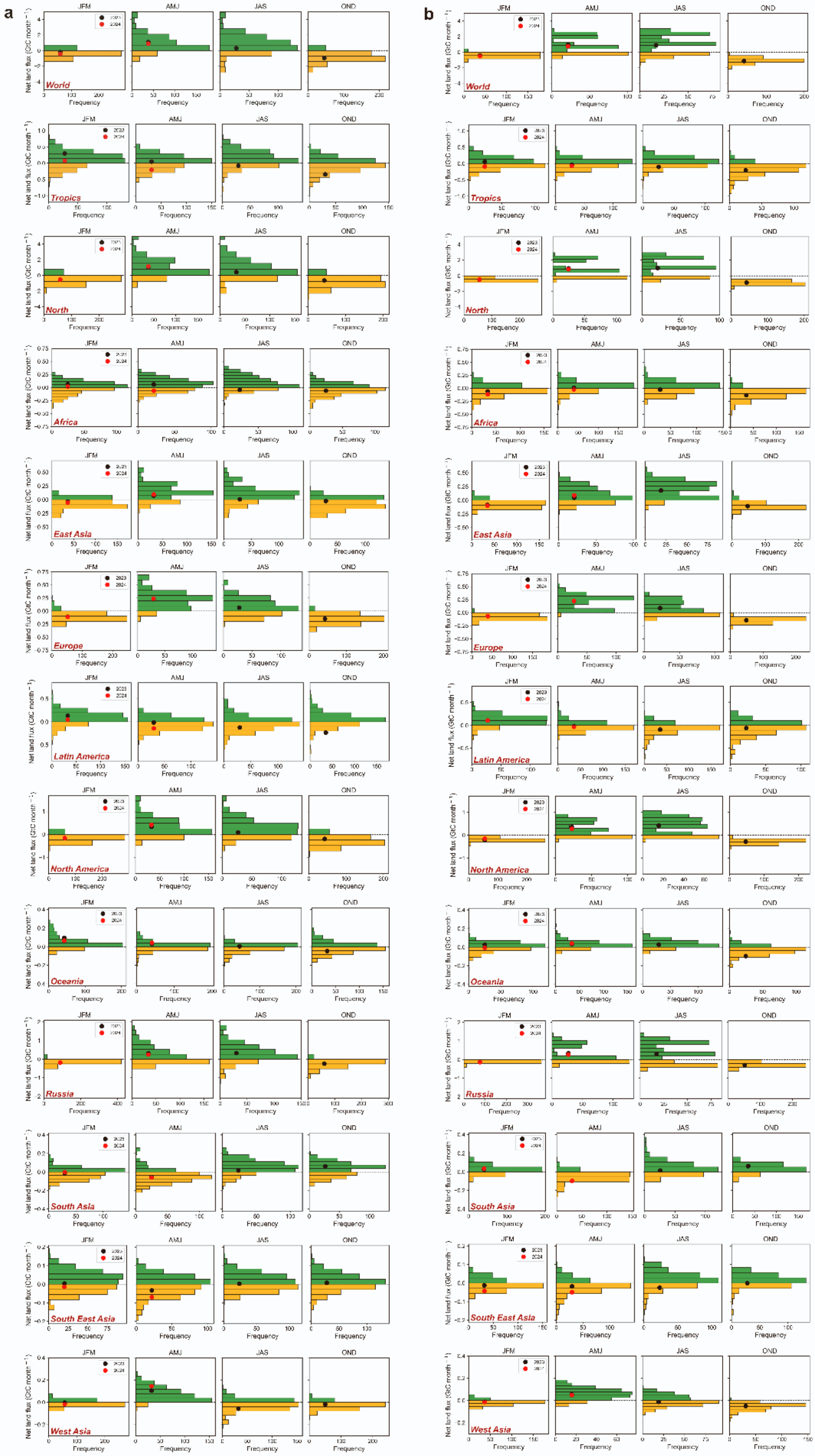

a
b
JFM
AMJ
JAS
OND
2023
2024
Net land flux (GtC month⁻¹)
Frequency
World
Tropics
North
Africa
East Asia
Europe
Latin America
North America
Oceania
Russia
South Asia
South East Asia
West Asia

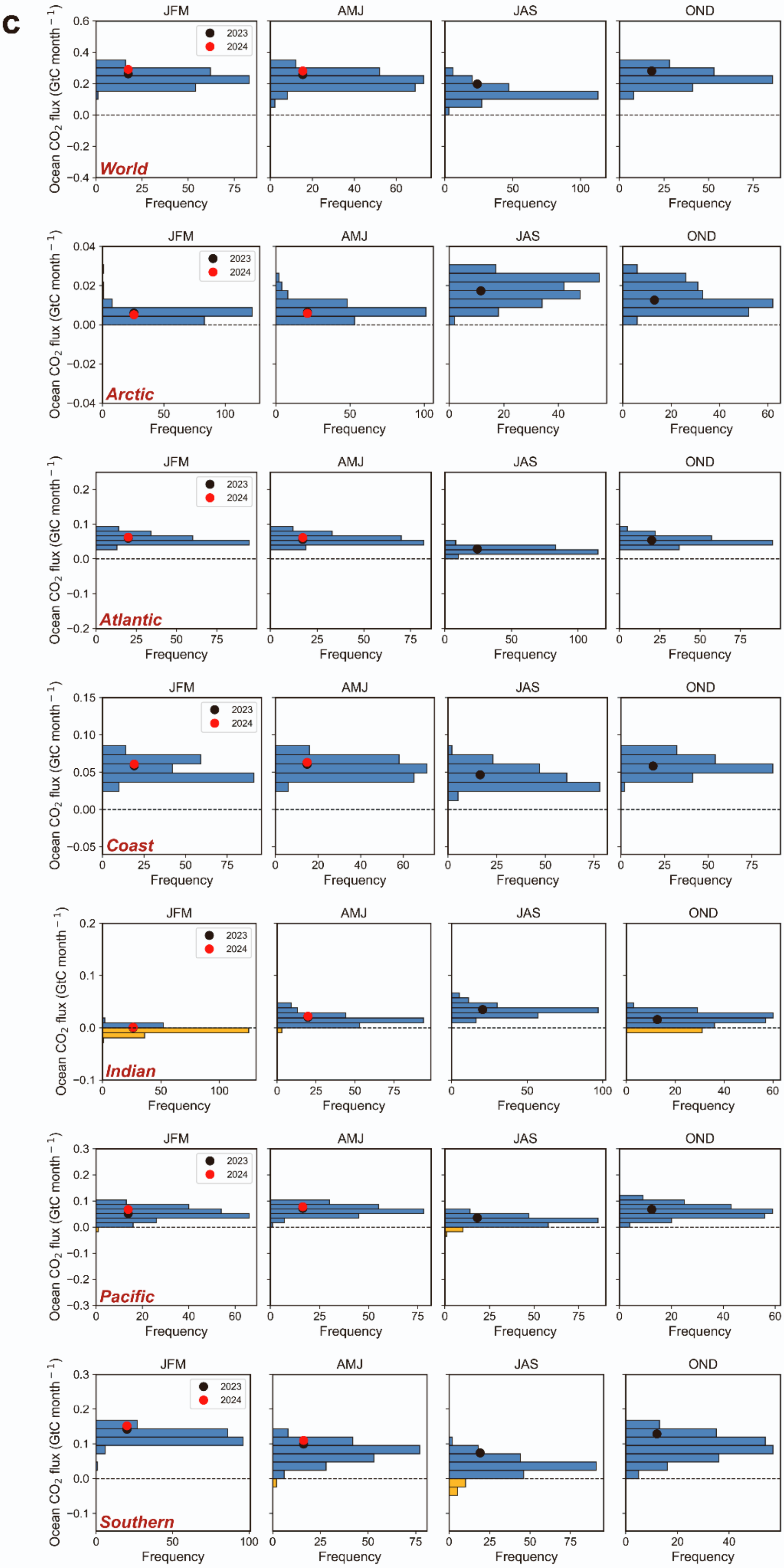

c
JFM
AMJ
JAS
OND
2023
2024
Ocean CO2 flux (GtC month−1)
Frequency
World
Arctic
Atlantic
Coast
Indian
Pacific
Southern

d

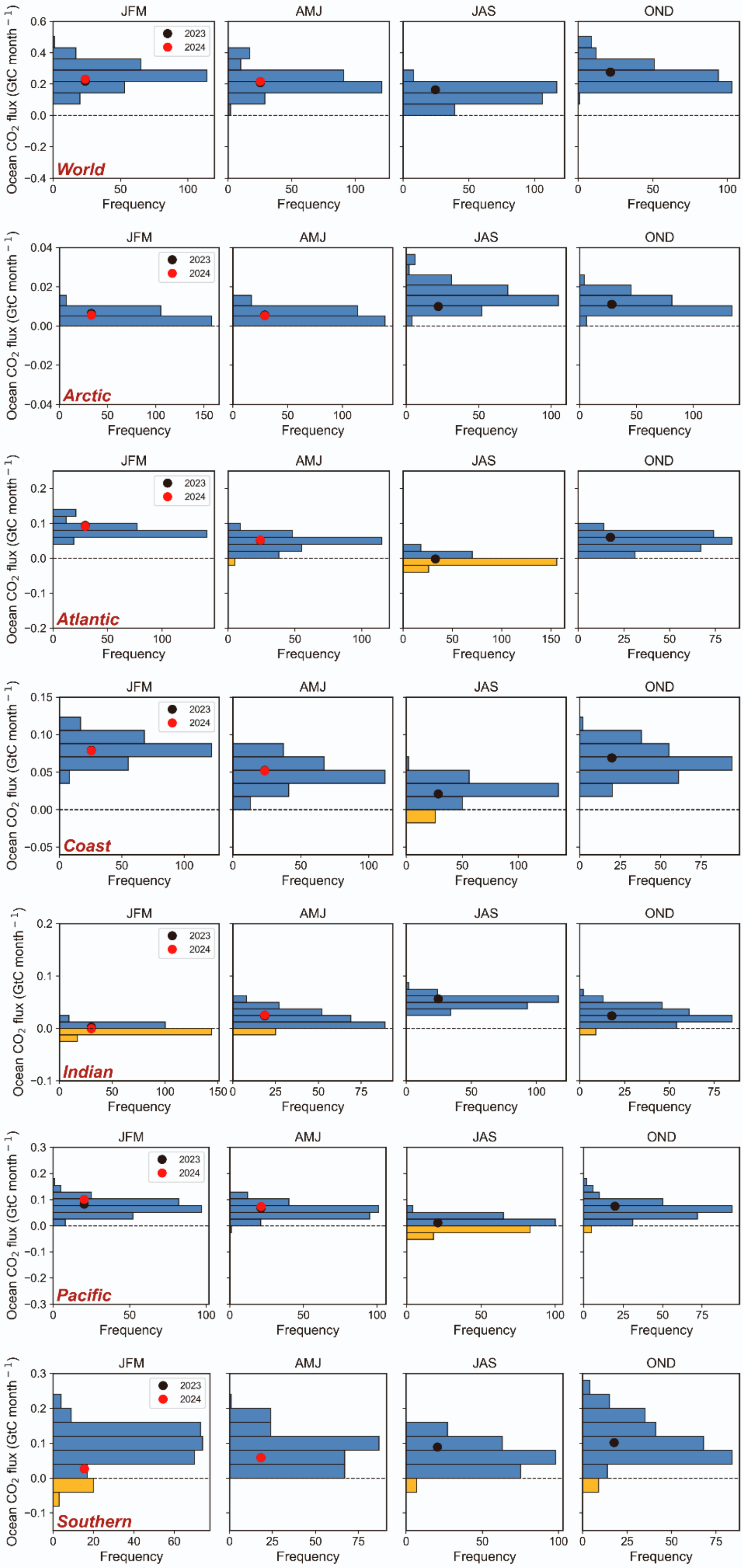
JFM
AMJ
JAS
OND
2023
2024
Ocean CO2 flux (GtC month−1)
Frequency
World
Arctic
Atlantic
Coast
Indian
Pacific
Southern

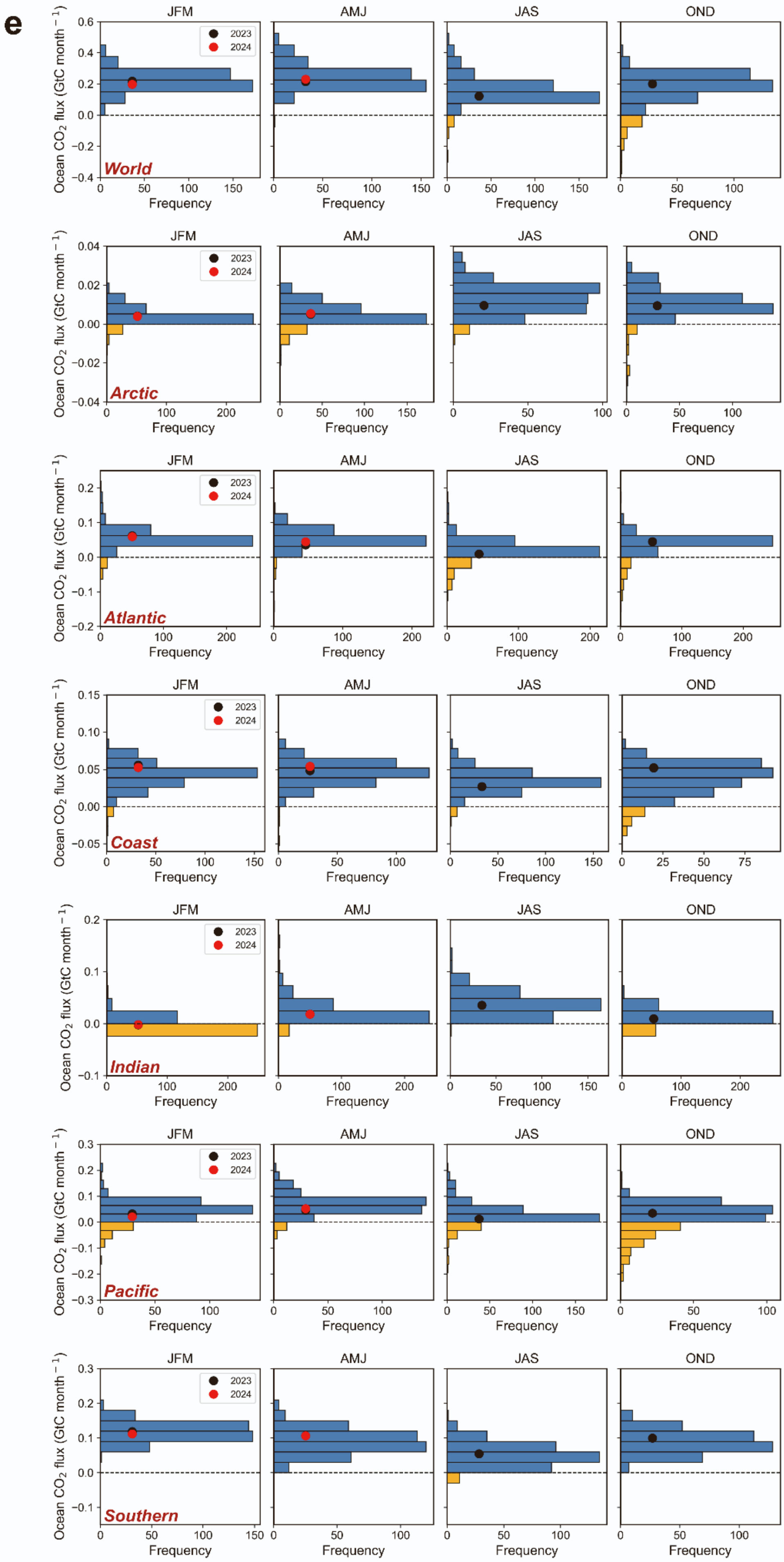
e
JFM
AMJ
JAS
OND
2023
2024
World
Arctic
Atlantic
Coast
Indian
Pacific
Southern
Ocean CO2 flux (GtC month−1)
Frequency

Supplementary Figure 6 Quarterly land and ocean fluxes for the RECCAP2 regions in 2023 (black dots) and in the first six months of 2024 (red dots). Fluxes are shown with the distributions of these fluxes during 2015-2023 with blue / green bars being positive values (sinks) and yellow ones negative values (sources). (a) Quarterly land flux for each RECCAP2 land region in the distribution of the fluxes from the DGVM models. **(b)** Quarterly land flux from the OCO-2 inversions for each RECCAP2 land region in the distribution of the flux from the inversion models. **(c)** Quarterly ocean flux from the ocean model emulators trained on GCB2024 ocean data-driven flux products for each RECCAP2 ocean region in the distribution of the flux from the data products models. (d) Quarterly ocean flux from the ocean model emulators trained on GCB2024 Global Ocean Biogeochemical Models (GOBM) for each RECCAP2 ocean region, in the distribution of the flux from the GOBMs. **(e)** Quarterly ocean fluxes from the OCO-2 inversions for each RECCAP2 ocean region in the distribution of the flux from the inversion models. See Supplementary Figure 5 for the maps of the RECCAP2 regions.

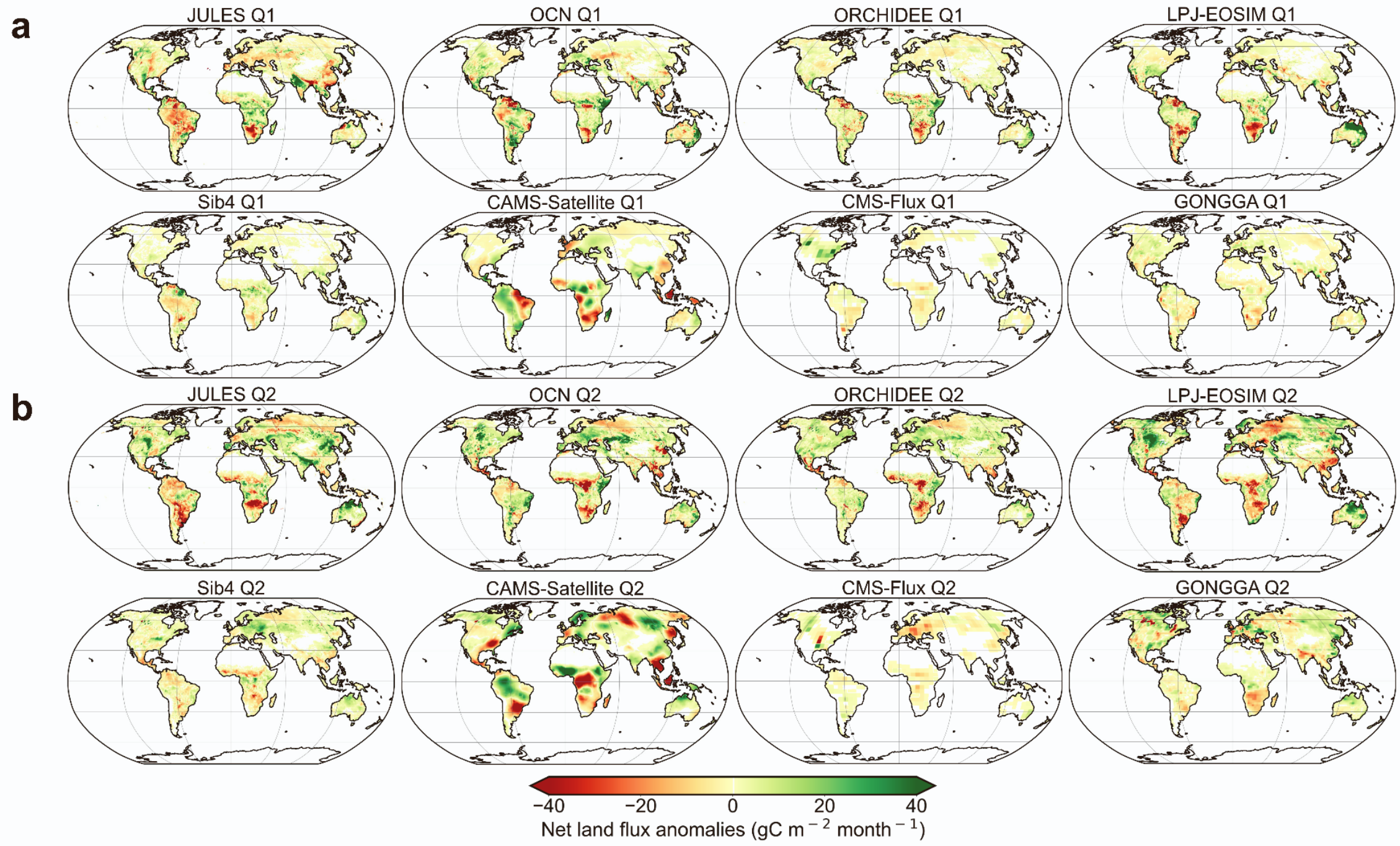

Supplementary Figure 7 Net land CO_2 flux anomalies for the first and second quarters in 2024 from each model. The top two rows show fluxes during JFM 2024 (Q1) and the bottom two during AMJ 2024 (Q2).

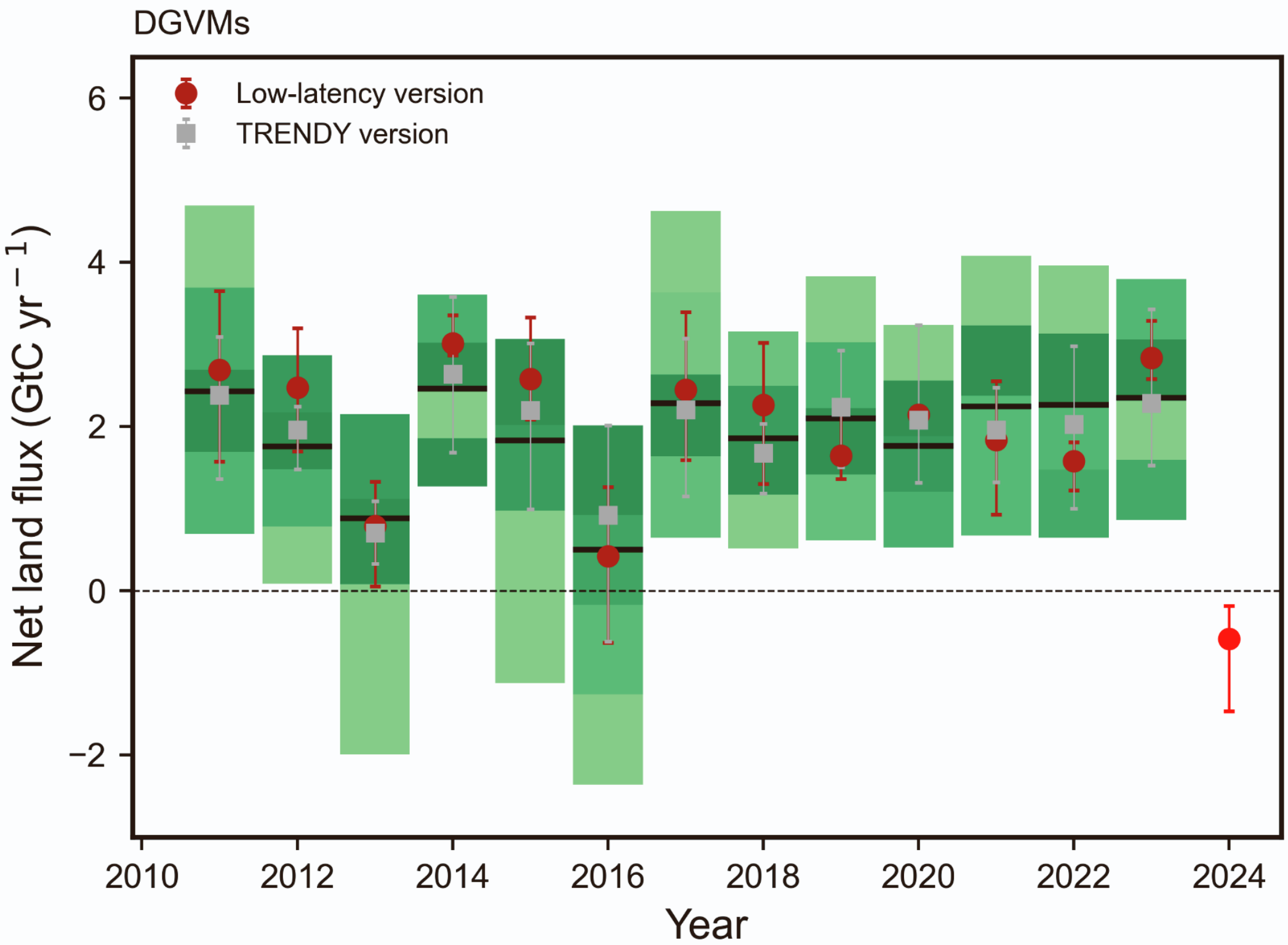

Supplementary Figure 8 Comparison of global net land CO_2 fluxes between the four DGVMs used in this study (red dots for Low-latency version, gray squares for TRENDY version) and the distribution of 22 TRENDY models (green bars, with color intensity representing the number of DGVMs falling into each interval) used in the Global Carbon Budget 2024. Positive values indicate flux from the atmosphere to the land (carbon sink).

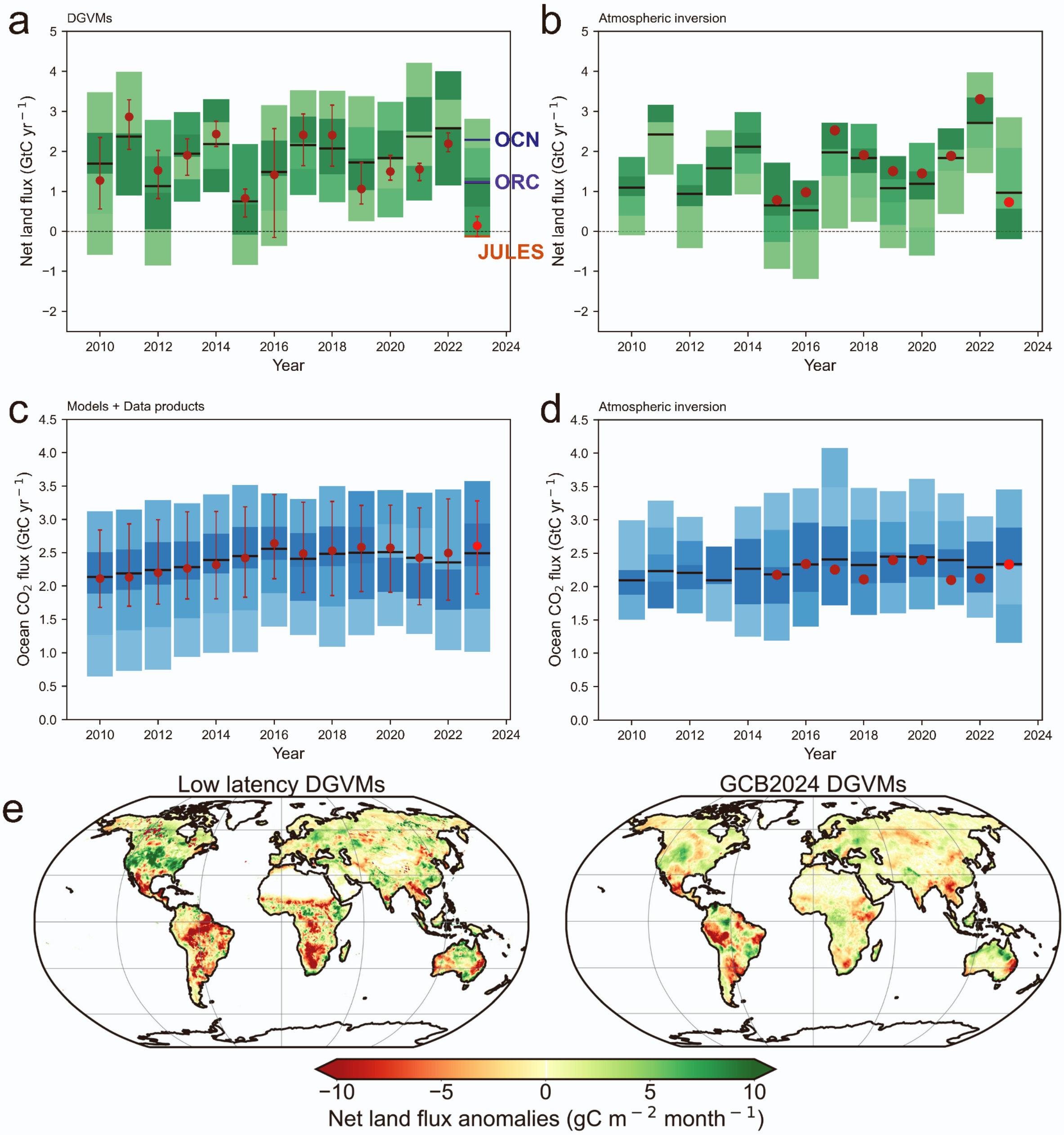

Supplementary Figure 9 Low latency budgets 2023 compared with the GCB 2024 model results. (a-d) The red dot shows the mean of the low latency models used in Ref. [9] and lines indicate the position of each DGVM model included in Ref. [9], as derived from the GCB24 results. Green bars (blue for ocean fluxes) indicate the range of the GCB2024 models with darker colors indicating that more models are in this interval. **(e)** Net land CO_2 flux anomalies in 2023 from the 2015-2022 period for the low latency DGVM models used by Ref. [9] for the CO_2 budget of the year 2023 and the mean of the TRENDY DGVMs used in GCB2024. Positive values (in green) represent increased flux from the atmosphere to the land or ocean (anomalous carbon sink).

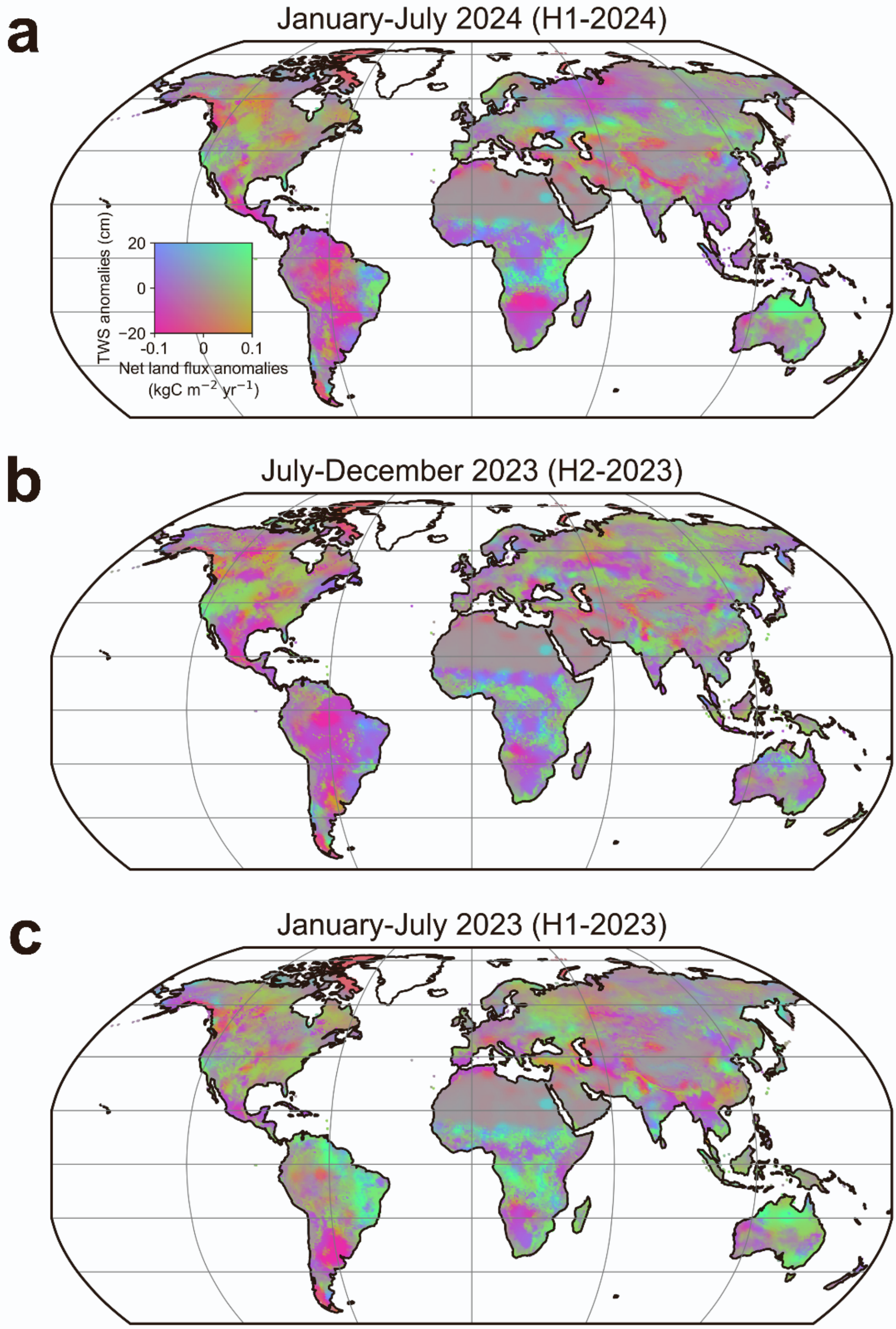

Supplementary Figure 10 Bivariate plots showing co-variations between net land flux anomalies from DGVMs and total water storage anomalies from the GRACE satellites in H1-2024, H2-2023 and H1-2023. Green areas show wetter anomalies coincident with more CO_2 uptake, and magenta areas show dryer anomalies that are coincident with reduced CO_2 uptake.

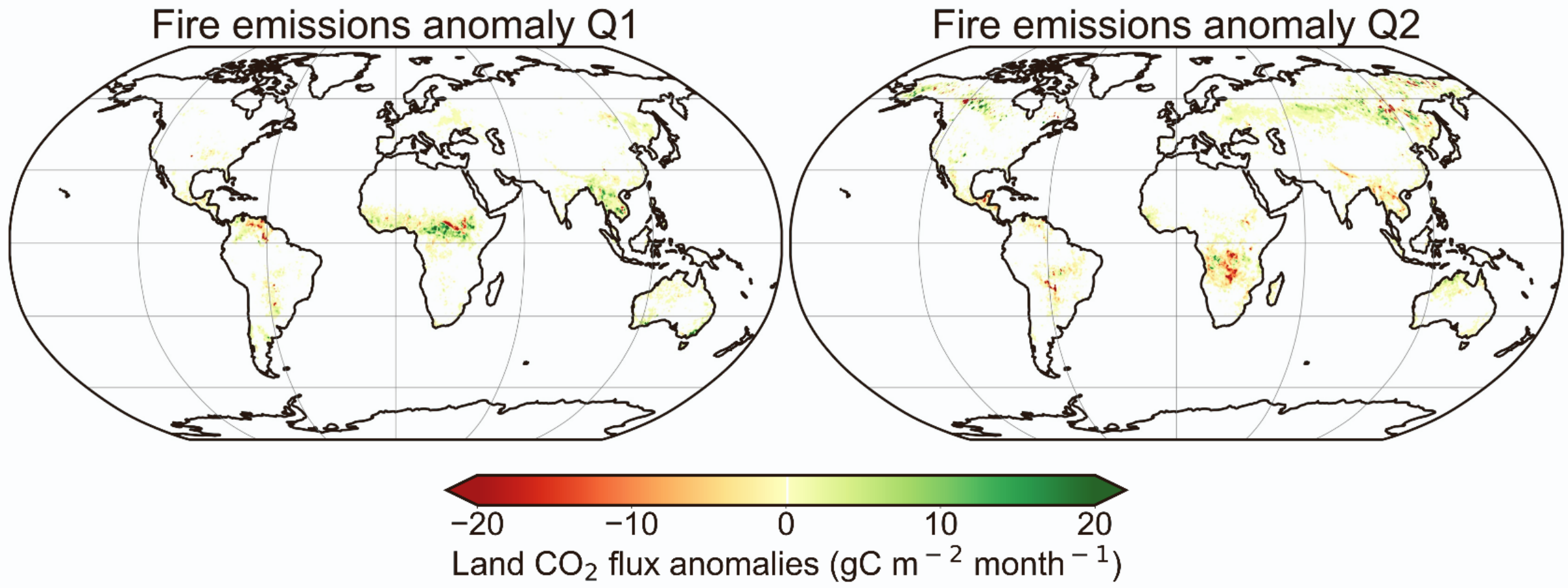

Supplementary Figure 11 Fire emission anomalies for the first and second quarters in 2024 (Q1 = JFM and Q2 = AMJ). Anomalies are calculated from the 2015-2022 average of each quarter. Positive values (in green) indicate decreased emissions, while negative values (in red) indicate increased emissions.

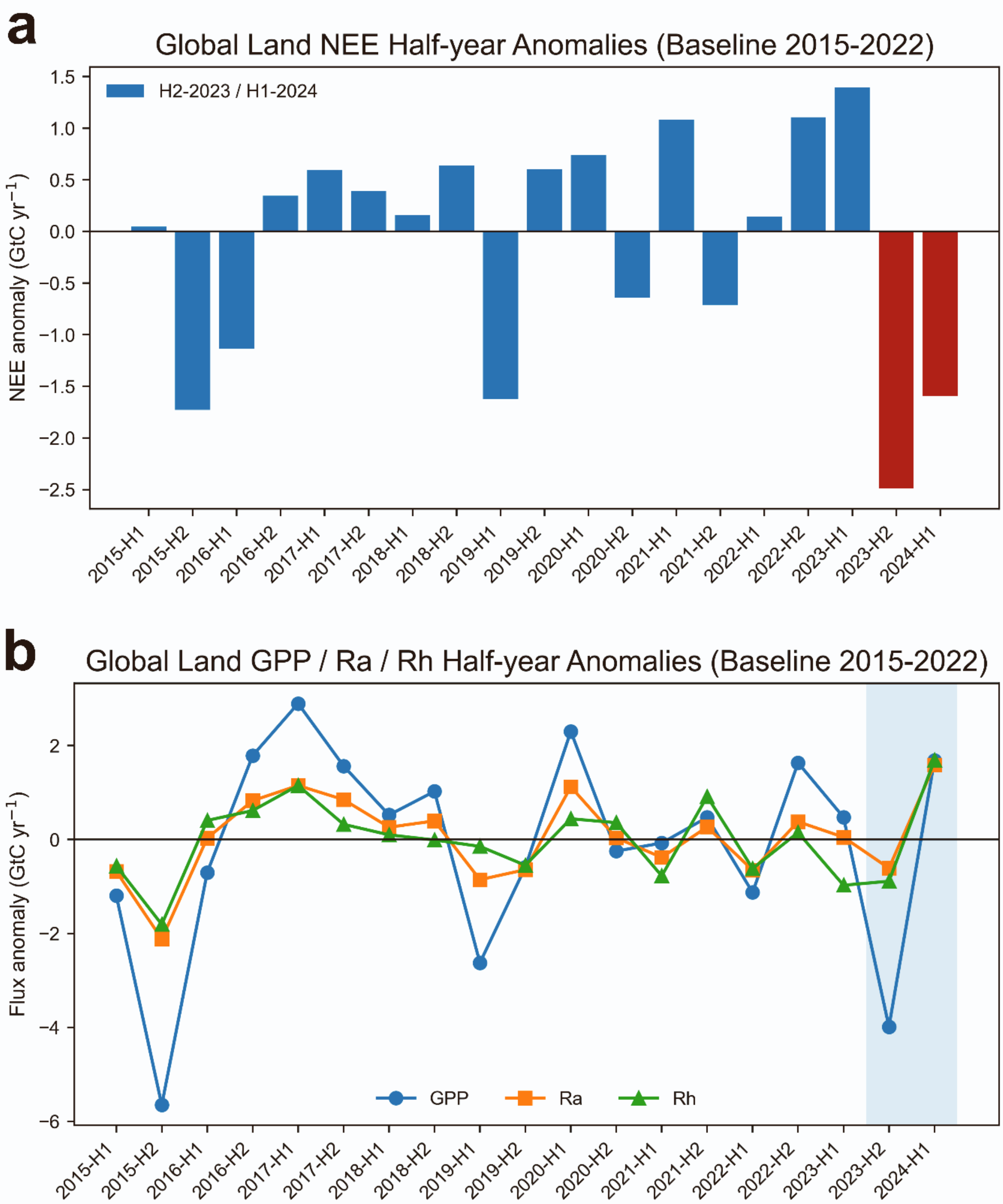

Supplementary Figure 12 Half-year anomalies of global land carbon flux components (Baseline: 2015–2022). (a) Anomalies of net ecosystem exchange (NEE) for each half-year period from the first half of 2015 (H1-2015) through the first half of 2024 (H1-2024). Negative values indicate reduced carbon uptake or enhanced carbon emissions by terrestrial ecosystems, while positive values represent increased carbon uptake. The red bars highlight the anomalously low carbon uptake periods coinciding with the 2023/24 El Niño event. (b) Corresponding half-year anomalies of gross primary production (GPP; blue), autotrophic respiration (Ra; orange), and heterotrophic respiration (Rh; green). The shaded area highlights the 2023/24 El Niño event period (H2-2023 to H1-2024).

Supplementary Table 1 CO_2 fluxes (2015–2022 average, 2023 and H1-2024) for each land RECCAP2 region from DGVMs and inversion methods (2015–2022 from GCB 2024; 2023-2024 from NRT methods; Unit: GtC yr^{-1}).

Period	Methods	World	Tropics	North	Africa	East Asia	Europe	Latin America	North America	Oceania	Russia	South Asia	Southeast Asia	West Asia
2015 -2022 average	DGVMs	1.86 ± 0.28	0.51 ± 0.11	1.21 ± 0.18	0.38 ± 0.06	0.14 ± 0.08	0.12 ± 0.01	0.18 ± 0.09	0.39 ± 0.07	0.13 ± 0.02	0.45 ± 0.05	0.03 ± 0.03	-0.06 ± 0.01	0.06 ± 0.04
	Inversions	1.47 ± 0.16	−0.39±0.11	1.80 ± 0.14	−0.38 ± 0.08	0.39 ± 0.11	0.18 ± 0.05	−0.14 ± 0.12	0.58 ± 0.09	0.08 ± 0.04	0.56 ± 0.07	0.13 ± 0.06	0.01 ± 0.06	−0.05 ± 0.04
2023	DGVMs	0.70 ± 0.24	-0.22 ± 0.54	0.94 ± 0.36	0.05 ± 0.17	0.07 ± 0.07	0.09 ± 0.11	-0.73 ± 0.25	0.17 ± 0.32	0.25 ± 0.27	0.65 ± 0.19	0.03 ± 0.07	-0.06 ± 0.10	0.09 ± 0.12
	Inversions	0.76 ± 0.59	-0.86 ± 0.61	1.63 ± 0.16	-0.62 ± 0.36	0.10 ± 0.13	0.28 ± 0.14	-0.17 ± 0.24	0.87 ± 0.67	-0.03 ± 0.14	0.61 ± 0.14	0.03 ± 0.12	-0.16 ± 0.06	-0.06 ± 0.14
H1-2024	DGVMs	2.73 ± 4.13	-0.78 ± 0.63	3.69 ± 4.00	-0.29 ± 0.13	0.24 ± 0.55	0.75 ± 0.35	-0.58 ± 1.17	1.57 ± 1.42	0.65 ± 0.51	0.35 ± 1.68	-0.37 ± 0.68	-0.49 ± 0.33	0.78 ± 0.21
	Inversions	1.42 ± 1.04	-0.97 ± 0.08	2.17 ± 1.02	-0.83 ± 0.73	-0.06 ± 0.30	0.94 ± 0.52	0.45 ± 1.27	0.72 ± 0.73	0.20 ± 0.19	0.79 ± 0.28	-0.37 ± 0.14	-0.55 ± 0.51	0.21 ± 0.12

Supplementary Table 2 CO_2 fluxes (2015–2022 average, 2023 and H1-2024) for each ocean RECCAP2 region from bottom-up and inversion methods (2010–2022 from GCB 2024; 2023-2024 from NRT methods; Unit: GtC yr^{-1}).

Period	Methods	World	Arctic	Atlantic	Coast	Indian	Pacific	Southern
2015-2022 average	GOBMs	2.44 ± 0.38	0.12 ± 0.03	0.51 ± 0.08	0.61 ± 0.11	0.27 ± 0.05	0.57 ± 0.12	1.06 ± 0.34
	Data products	2.52 ± 0.34	0.13 ± 0.04	0.56 ± 0.09	0.64 ± 0.12	0.18 ± 0.07	0.61 ± 0.13	0.99 ± 0.14
	Inversions	2.34 ± 0.45	0.09 ± 0.05	0.50 ± 0.13	0.48 ± 0.11	0.21 ± 0.09	0.50 ± 0.21	1.03 ± 0.17
2023	GOBMs	2.66 ± 0.50	0.10 ± 0.02	0.60 ± 0.13	0.66 ± 0.12	0.33 ± 0.11	0.73 ± 0.32	0.89 ± 0.40
	Data products	2.88 ± 0.28	0.11 ± 0.04	0.59 ± 0.08	0.64 ± 0.09	0.21 ± 0.05	0.69 ± 0.28	1.32 ± 0.09
	Inversions	2.26 ± 0.48	0.09 ± 0.02	0.46 ± 0.04	0.55 ± 0.09	0.18 ± 0.06	0.38 ± 0.15	1.14 ± 0.43
H1-2024	GOBMs	2.82 ± 0.56	0.06 ± 0.01	0.88 ± 0.12	0.75 ± 0.14	0.14 ± 0.12	1.00 ± 0.37	0.54 ± 0.58
	Data products	3.32 ± 0.53	0.06 ± 0.03	0.73 ± 0.11	0.71 ± 0.13	0.14 ± 0.06	0.94 ± 0.38	1.58 ± 0.12
	Inversions	2.57 ± 0.31	0.06 ± 0.01	0.63 ± 0.14	0.64 ± 0.14	0.10 ± 0.08	0.44 ± 0.39	1.31 ± 0.46

Supplementary Table 3 Cross-dataset comparison of H1-2024 land CO_2 flux anomalies. Land CO_2 flux anomalies are shown for the primary ERA5-driven DGVM ensemble, the OCO-2 inversion ensemble, and two independent cross-check products, the 19-DGVM emulator ensemble and SiB4. Values are H1-2024 integrated carbon anomalies in GtC relative to the 2015–2022 mean for the same January–June period. Positive anomalies indicate stronger uptake or a weaker source; negative anomalies indicate weaker uptake or a stronger source. Values for DGVMs, inversions, and emulators are ensemble mean ±1σ across models or products; SiB4 is reported as a single-product estimate.

Region	ERA5-driven DGVMs	OCO-2 inversions	19-DGVM emulators	SiB4
World land	-0.90 ± 0.40	-0.27 ± 0.06	-0.17 ± 0.31	-0.21
Tropics	-0.92 ± 0.17	-0.26 ± 0.04	-0.34 ± 0.25	-0.38
Northern land >30°N	0.20 ± 0.21	0.08 ± 0.06	0.20 ± 0.14	0.22
Latin America north of 30°S	-0.53 ± 0.26	-0.03 ± 0.12	-0.21 ± 0.21	-0.34
Tropical Africa	-0.41 ± 0.16	-0.17 ± 0.02	-0.15 ± 0.10	-0.13
Southeast Asia	-0.16 ± 0.04	-0.15 ± 0.20	-0.08 ± 0.06	0.00

Supplementary Table 4 Comparison of ERA5 and CRU-JRA precipitation anomalies in key tropical regions during JFM and AMJ 2024. Precipitation anomalies are calculated relative to the 2015–2022 mean for the same calendar period and are expressed in mm $month^{-1}$ and percent. ERA5 total precipitation was converted to monthly totals according to its monthly averaged daily accumulation convention, whereas CRU-JRA precipitation was summed from 6-hourly accumulated precipitation fields with units of mm per 6 hours. Positive values indicate wetter-than-baseline conditions and negative values indicate drier-than-baseline conditions.

Region	Period	ERA5 anomaly (mm $month^{-1}$)	CRU-JRA anomaly (mm $month^{-1}$)	ERA5 − CRU-JRA (mm $month^{-1}$)	ERA5 anomaly (%)	CRU-JRA anomaly (%)
Tropics	JFM-2024	-5.5	-1.1	-4.4	-4.6	-3.9
Tropics	AMJ-2024	-6.8	0.0	-6.7	-6.6	-0.1
Latin America north of 30°S	JFM-2024	-23.3	-2.5	-20.8	-13.0	-6.3
Latin America north of 30°S	AMJ-2024	-9.1	0.5	-9.7	-6.3	1.7
Tropical Africa	JFM-2024	-7.5	-0.8	-6.7	-10.8	-4.8
Tropical Africa	AMJ-2024	-11.9	-0.6	-11.3	-21.6	-4.0
Southeast Asia	JFM-2024	2.7	-1.1	3.9	1.4	-2.6
Southeast Asia	AMJ-2024	18.8	1.5	17.4	7.8	2.9
